# A morphometrics-informed reconstruction of the Early Devonian zosterophyll *Nowenia matsunagae* gen. et sp. nov. as a template for building detailed empirically supported whole-plant concepts of early tracheophytes with simple body plans

**DOI:** 10.1093/aob/mcag040

**Published:** 2026-02-25

**Authors:** Samar R El-Abdallah, Pénélope Claisse, Candela Blanco-Moreno, Alexandru M F Tomescu

**Affiliations:** Department of Biological Sciences, California State Polytechnic University, Humboldt, Arcata, CA 95521, USA; Evo-Eco-Paléo, École Doctorale Sciences de la Matière, du Rayonnement et de L'environnement, Université de Lille, Lille 59000, France; Departamento de Biología, Facultad de Ciencias, Universidad Autónoma de Madrid, Madrid 28049, Spain; Department of Biological Sciences, California State Polytechnic University, Humboldt, Arcata, CA 95521, USA

**Keywords:** Devonian, fossil, growth habit, Lycophytina, morphometrics, *Nowenia matsunagae*, whole-plant concept, Wyoming, zosterophyll

## Abstract

**Background and Aims:**

The fragmentary state of plant fossils and the modular organization of plants make whole-plant reconstructions of fossil species desirable and feasible. Such reconstructions are key for integrating fossil species in systematic studies. The ∼410 Ma Beartooth Butte Formation of Wyoming (USA) hosts the only rich Early Devonian flora in western North America, which fills a major gap in the phytogeography of this interval. We construct a whole-plant concept for a new zosterophyll from the Beartooth Butte Formation based on detailed morphometric study of a large number of specimens.

**Methods:**

More than 600 fragments of the new zosterophyll were observed and 200 of these were measured. Epidermal features were observed in cuticular material recovered on cellulose acetate. Resulting data were used to characterize variability in axis thickness and taper, branching density and sporangial orientation, based on descriptive statistics, correlations and principal component analysis.

**Key Results:**

The new zosterophyll, *Nowenia matsunagae* gen. et sp. nov., consisted of decumbent axes with long internodes that exhibited K-branching and bore more densely branched erect axes, as well as branches with delayed development (dormant lateral meristems and circinate protrusions). Axes had circinate apices and bore isolated bivalvate sporangia in small numbers, primarily on upright portions. Phylogenetic analyses recover *Nowenia* sister to *Forania*, from which it differs primarily in the absence of spinescent protrusions.

**Conclusions:**

*Nowenia* is the first zosterophyll for which an empirically based whole-plant concept that makes explicit and extensive use of quantitative data is available. The approach used to reconstruct the *Nowenia* plant introduces a method for integrating morphometric data in constructing whole-plant concepts of early tracheophytes with simple body plans. Application of this and similar methods to additional fossil species could produce reconstructions at similar levels of detail and accuracy, crucial for reaching well-supported resolution of early vascular plant relationships.

## INTRODUCTION

Tracheophyte diversity and morphological complexity increased tremendously during the Early Devonian (419–393 Ma), when most plant communities occupied environments in the vicinity of water bodies, in coastal and fluvial settings, and were dominated by zosterophylls (Gensel, 1992; Hotton *et al.*, 2001). Present in the fossil record since the late Silurian (Kotyk, 1998), zosterophylls peaked in diversity during the Early Devonian (Cascales-Miñana and Meyer-Berthaud, 2014) and have been documented in strata all around the world. Throughout the Middle Devonian, zosterophyll diversity decreased and the group is absent from rocks younger than the Late Devonian (Gensel and Andrews, 1984; Cascales-Miñana and Meyer-Berthaud, 2014). As is the case for most early tracheophytes, zosterophyll diversity is known primarily from fossils preserved as adpressions, with cellular permineralization comparatively infrequent (Edwards *et al.*, 1982; Gensel and Andrews, 1984; Nibbelink and Tomescu, 2022).

Plant fossils often occur as fragments, which may render taxonomic decisions difficult (e.g. Tanner, 1982), hinders their inclusion in morphological phylogenetic datasets or lowers the resolution of phylogenetic trees (Gensel, 1992; Kenrick and Crane, 1997; Crepet and Niklas, 2019; Nibbelink and Tomescu, 2022). In the absence of complete fossils, these shortcomings are mitigated by reconstructing fossil plants as whole organisms, which is achieved at highest accuracy by morphometric analyses that rely on quantitative data. In turn, the conclusiveness of such analyses increases with the number of specimens available. The resulting empirically supported organismal concepts not only provide detailed insights on the morphology and growth habit of the plants, but can provide valuable data for phylogenetic analyses. For example, the reconstruction of *Sengelia radicans* into a whole-plant concept based on morphometric data from hundreds of fragmented specimens, led to changes in drepanophycalean lycopsid taxonomy and filled key gaps in our understanding of root evolution (Matsunaga and Tomescu, 2016, 2017).

In North America, most of the rock units containing Early Devonian plant fossils are concentrated in the eastern side of the continent. The Beartooth Butte Formation is the only Early Devonian unit on the western side of the continent that hosts abundant plant fossils (Tanner, 1983). Consequently, the plant fossil assemblages of the Beartooth Butte Formation hold a key position in the floristics and biogeography of the Early Devonian. To date, 14 potential taxonomic types (most of them not formally named, to date) have been recognized in the Beartooth Butte Formation (Bippus and Tomescu, 2017). However, the only comprehensive treatment of the Beartooth Butte Formation flora, as represented in the collections available at the time, was described in the unpublished PhD dissertation of Tanner (1983). Since then, *Sengelia* has been the only plant of the Beartooth Butte Formation to be re-described and formally published (Matsunaga and Tomescu, 2017). Within the formation, the Cottonwood Canyon locality assemblages are much more densely sampled than those at the Beartooth Butte locality. Additional collecting at Cottonwood Canyon by several groups since Tanner’s studies allows in-depth re-assessment of the taxonomic diversity in the fossil assemblages. Studies employing morphometric plant reconstruction methods pioneered in the *Sengelia* study can provide further insights into early tracheophyte morphology, floristics, systematics and evolution.

Here, we introduce an empirically supported whole-plant reconstruction of a new zosterophyll genus and species informed by morphometric analyses of one of the most abundant fossil plant types in the Cottonwood Canyon locality.

## MATERIALS AND METHODS

### Geological setting

The Early Devonian Beartooth Butte Formation, with exposures in northern Wyoming and southern Montana, hosts fossil plant assemblages at two localities: Beartooth Butte and Cottonwood Canyon (Dorf, 1933, 1934; Blackstone and McGrew, 1954; Sandberg, 1961; Tanner, 1983). Our study is based on material collected from Cottonwood Canyon (Big Horn County, Wyoming; 44°51′51″N, 108°02′46″W). At Cottonwood Canyon, the Beartooth Butte Formation consists of interlayered shale, siltstone and silty or sandy dolomite (Sandberg, 1967; Matsunaga and Tomescu, 2017). These layers contain terrestrial plant material as well as fish and eurypterid fossils (Elliott and Johnson, 1997; Lamsdell and Legg, 2010; Lamsdell and Selden, 2013) and are thought to represent fluvial or estuarine deposits (Sandberg, 1961; Fiorillo, 2000; Matsunaga and Tomescu, 2017). A recent palynological study (Noetinger *et al.*, 2021) supports a late Lochkovian to Pragian age (∼415–411 million years) for the plant-fossiliferous layers of the Beartooth Butte Formation at Cottonwood Canyon, consistent with previous age estimates based on palynomorph and fish biostratigraphy (D.C. McGregor, in Elliott and Ilyes, 1996; Elliott and Johnson, 1997).

### Plant material and observations

This study is based on observations of more than 600 plant axes representing the new species described here. Morphometric analysis of a subset of the specimens allowed the characterization of the branching architecture and growth habit of the new species, understanding its growth dynamics and preparing a whole-plant reconstruction.

The fossil plant axes included in this study come from several levels of a ∼2-m thick heterolithic sequence that consists of an alternation of dark, finely laminated shales that preserve dense *in situ* populations of the early lycopsid *Sengelia*, and massive beds of hard cemented siltstones rich in transported plant material and other organic detritus. This alternation has been interpreted as a series of periodic flood events that buried multiple successive *Sengelia* populations (Matsunaga and Tomescu, 2017; Noetinger *et al.*, 2021). In this heterolithic sequence, the material included in this study comes from the beds interpreted as flood deposits.

**Fig. 1.**
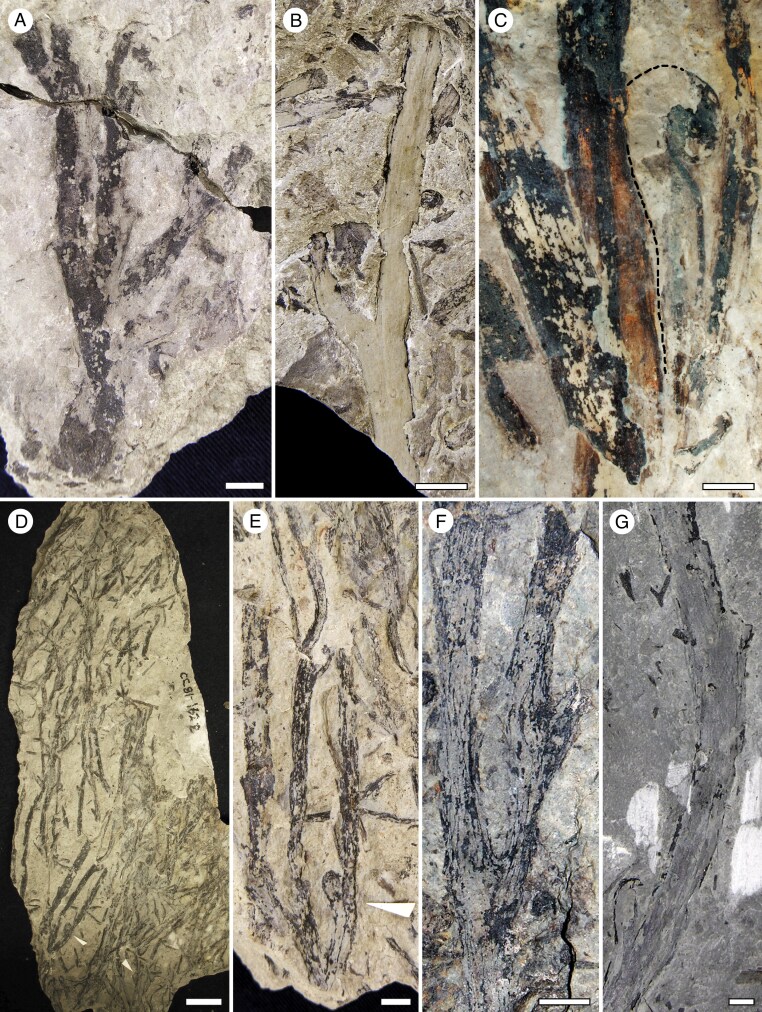
*Nowenia matsunagae* gen. et sp. nov. (A–C) Different preservation modes. (A) Coalified compression. Scale bar = 5 mm. HPH386 (see also Figs 4C and 5C). (B) Impression with rare fragments of carbonaceous material; note characteristic U-shaped branching. Scale bar = 5 mm. HPH317. (C) Several axes showing different preservation modes: coalified compression (left; note fine longitudinal lines, a common feature used to identify fragments of *Nowenia*); oxidized, with coaly vascular strand (centre); impression with coaly fragments and conspicuous coalified vascular strand (right; note circinate apex partially preserved as impression only, with outline traced for clarity). Scale bar = 2 mm. HPH541. (D) Mat of intertangled axes. Scale bar = 2 cm. KU D1588b. (E) Detail of (D); note variable axis thickness and sinuous longitudinal creases interpreted as the result of drying-induced shrinking, and dormant branch bud (opposite the arrowhead on axis at right). Scale bar = 5 mm. (F) Axis showing characteristic U-shaped branching pattern and many fine sinuous longitudinal lines. Scale bar = 3 mm. HPH792. (G) Axis bearing subtle, fine longitudinal lines and dormant branch bud (bottom left). Scale bar = 3 mm. FM PP15956 (see also Figs 4N and 5N).

**Fig. 2.**
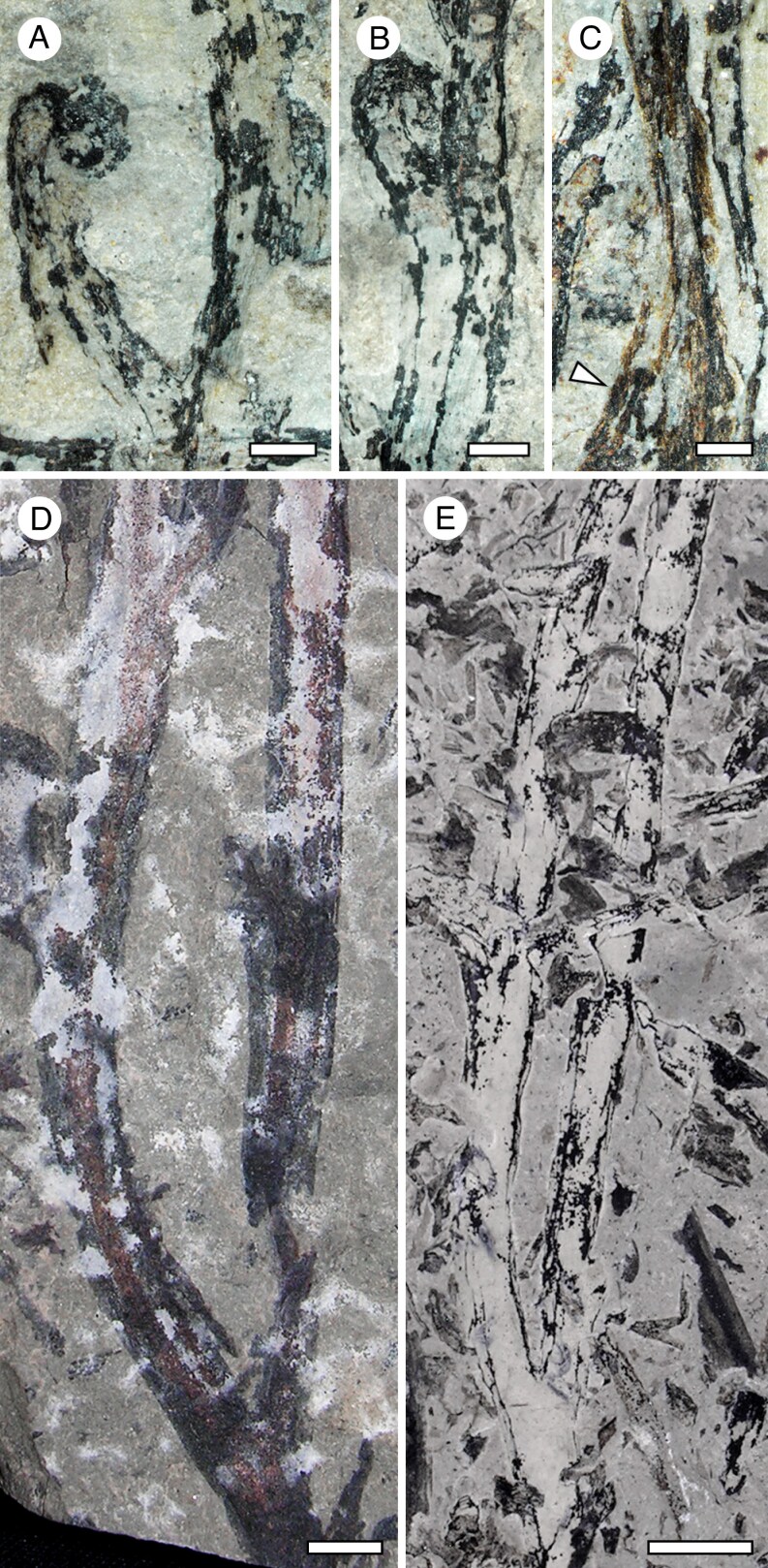
*Nowenia matsunagae* gen. et sp. nov. (A–C) Characteristic fine sinuous lines along axes; note lateral branch with circinate tip (A), crozier-shaped branch bud (B) and faint outline of a sporangium (arrowhead) attached to axis with oxidation zones (C). Scale bars = 2 mm. KU D1588b. (D, E) Characteristic U-shaped branching with lateral branches diverging at narrow angles and curving close to their base to become more or less parallel to the subtending axis; note oxidized vascular strand in (D). (D) Scale bar = 5 mm. FM PP49074 (see also Figs 4P and 5P). (E) Scale bar = 10 mm. HPH359 (see also Figs 4L and 5L).

The plant axes are preserved primarily as coalified compressions, some with cuticular material, but some are preserved as impressions with rare fragments of coalified material present (Fig. 1A, B). Some of the axes exhibit light traces of oxidation (Fig. 1C). The axes were recognized as conspecific based on several lines of evidence. The axes are smooth, lack surface projections, and often bear fine sinuous longitudinal creases (Fig. 2A–C; see also Fig. 1C, E–G); some axes have a sinuous habit. Branches diverge at acute angles and curve apically close to their base, to run roughly parallel to their subtending axes, forming a more or less U-shaped pattern (as opposed to the typical overall V-shape of branching in most plants), e.g. the U pattern of Gensel (1982) (Fig. 2D, E); subaxillary tubercles are absent. Branches in early developmental stages occur as knob-like meristems lateral on axes; when more developed, they have circinate tips (Fig. 2A, B).

Axes of the new species often occur in monotypic or monodominant associations (Fig. 1C; Supplementary Data Fig. S1). In some cases the monotypic associations form mats (Fig. 1D; Supplementary Data Fig. S1), some of which show evidence of shrinking of the axes prior to preservation: irregular width and fine sinuous longitudinal folds or ridges (Fig. 1E–G; Supplementary Data Fig. S1). Although specimens within the mats are relatively large, their extensive intertangling renders measurements difficult and they were not used in the morphometric analyses. A significant proportion of the axes are preserved as isolated specimens, which provided most of the morphometric data. Their size is highly variable, from short fragments with a single branching point (or none) to more extensive specimens that provide information on the branching architecture.

Part of the fossil material was collected between 2007 and 2017 by different Humboldt State University field crews and is held in the Humboldt Paleobotanical Herbarium (HPH) at California State Polytechnic University, Humboldt (Cal Poly Humboldt; Arcata, CA, USA). Specimens collected by previous workers (Charles Sandberg (1960s), Francis Hueber (1970s), William Tanner (1970–1980s) and Kirk Johnson (1990s)) housed in the Biodiversity Institute & Natural History Museum of the University of Kansas (KU; Lawrence, KS, USA), the Field Museum (FM; Chicago, IL, USA) and the US National Museum of Natural History, the Smithsonian Institution (USNM; Washington, DC, USA) were examined on pictures or directly on loaned specimens.

The fossils were imaged using a Nikon Coolpix 8800 VR camera (Tokyo, Japan), by itself or mounted on a Nikon Eclipse E400 compound microscope (Tokyo, Japan), or a CellSens camera mounted on an Olympus SZX 16 microscope (Tokyo, Japan). Some specimens were imaged immersed in 100 % isopropyl alcohol, while others were imaged without alcohol immersion. Macroscopic measurements were taken either directly on the specimens, using a Mitutoyo 530-115N 12-inch vernier calliper (Sakado, Japan), or on scaled photographs, using ImageJ (US National Institutes of Health).

Because fossil preservation sometimes rendered observation of their morphology difficult, the most extensive specimens were traced in Adobe Illustrator to accurately document their overall morphology.

Cuticles were collected by dissolving pieces of cellulose acetate onto plant specimens, using acetone; upon drying, the acetate was peeled off, retrieving the plant cuticle that was embedded in it, and mounted on microscope slides using Eukitt (O. Kindler GmbH, Freiburg, Germany).

The whole-plant reconstruction is supported by quantitative data obtained from morphometric analyses and reflects ranges of variation of as many of the measured and calculated morphological features as possible.

### Collection and processing of morphometric data

**Fig. 3.**
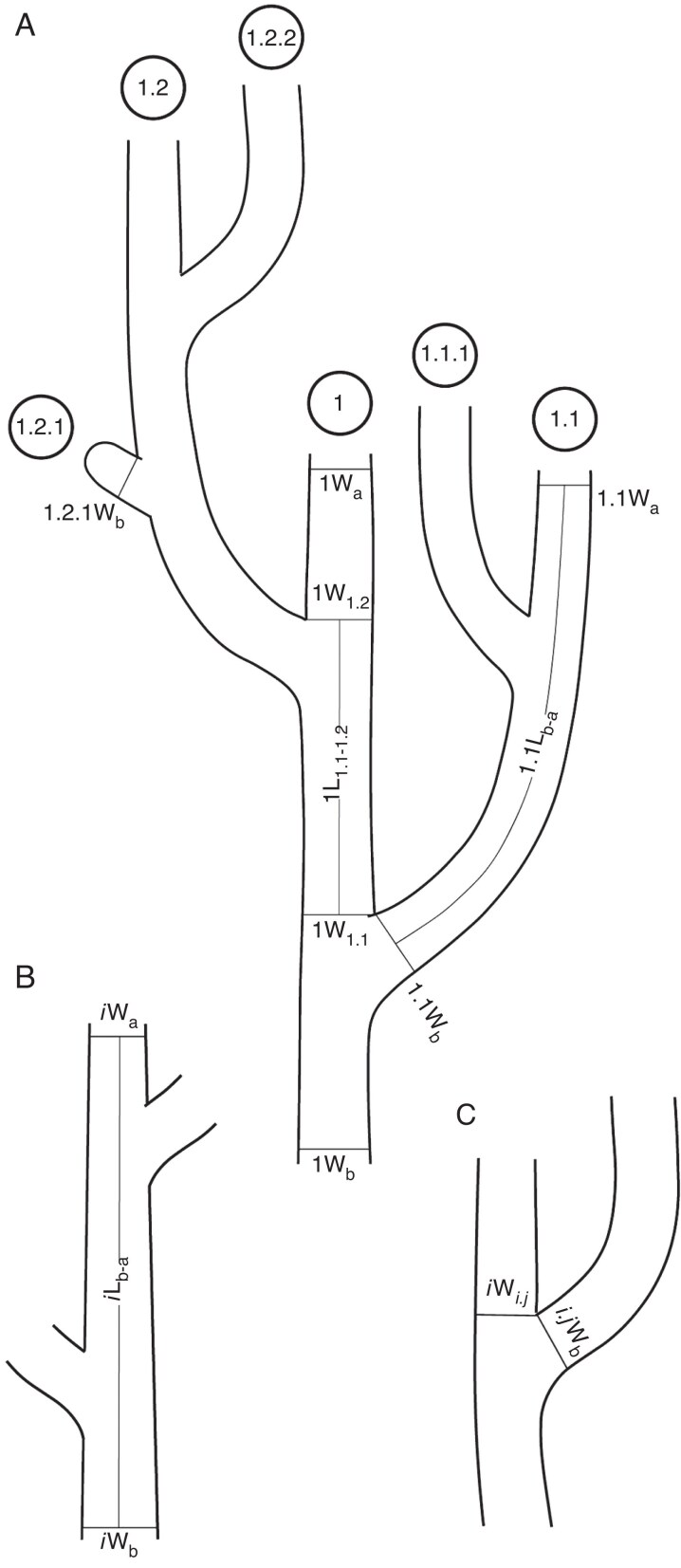
Locations of reference points and notations used for measurements of axis morphological variables. (A) Notations used for branching orders and individual branches (exemplified for an axis bearing two orders of branching). 1, main axis of specimen; 1.1, 1.2, etc., first-order branches from base to top of main axis; 1.2.1, 1.2.2, etc., second-order branches of the second branch of first order (1.2); axis width measurements are denoted as the axis number, followed by ‘W’ and the position of the measurement point indicated in subscript (‘b’ for basal-most measurement on that axis subscript, ‘a’ for apical-most measurement, or number designating the branch above which the measurement is taken, e.g. 1W_1.1_); length measurements are denoted as the axis number followed by ‘L’ and a subscript indicating the two successive branching points between which the length is measured (internode) (e.g. 1L_1.1–1.2_); total lengths from base to top are denoted by the subscript ‘b-a’ (e.g. 1L_b–a_). (B) The taper coefficient is calculated by subtracting the apical-most width of an axis *i* from its basal-most width and dividing the difference by the length between them. (C) The coefficient of branch subordination is calculated by dividing the width an axis *i* at the position of a branch *j* by the basal-most width of *j*.

*Measurements*. The branching hierarchy of individual specimens is referenced using the following notation system: 1 (main axis of the specimen); 1.1, 1.2, …, 1.*n* (first-order branches, with the basal-most numbered 1.1); 1.1.1, 1.1.2, …, 1.1.*n* (second-order branches of branch 1.1, with the basal-most numbered 1.1.1); 1.2.1, 1.2.2, …, 1.2.*n* (second order branches of branch 1.2, with the basal-most numbered 1.2.1); 1.1.1.1, 1.1.1.2, …, 1.1.1.*n* (third-order branches of branch 1.1.1, with the basal-most numbered 1.1.1.1); etc. (Fig. 3A).

Measured lengths are designated by the position of the measured axis in the branching hierarchy (1, 1.1, 1.2, … 1.1.1, 1.1.2, …, 1.1.1.1, 1.1.1.2, …) followed by the letter ‘L’ and by either ‘b–a’ for the entire length from base to apex (1L_b–a_), or the notation for a specific axis segment between two successive branching points (e.g. 1L_b–1.1_ for the segment between the base of the axis fragment and the lowermost branching point; 1.1L_b–1.1.1_ for the segment of branch 1.1 between its base and its basal-most branch; 1.2L_1.2.1–1.2.2_ for the segment of branch 1.2 between its two basal-most branches) (Fig. 3A, B).

The width of each axis (assumed to be representative of the original axis diameter; Rex and Chaloner, 1983) was measured at the basal-most point where it preserved the entire width, immediately above the base of each branching point, and at the apical-most point where it preserved the entire width. The widths are designated by the position of the measured axis in the branching hierarchy (1, 1.1, 1.2, … 2.1, 2.2, …, 1.1.1, 1.1.2, …), followed by the letter ‘W’ and by the position along the axis where the measurement was taken (‘b’ for basal, ‘a’ for apical, 1.1, 1.2, … for the branch above which the width was measured; e.g. 1W_b_, 1W_a_, 1W_1.1_ …) (Fig. 3).

The widths of branches at the base were measured from the axil of the branch along a line perpendicular to the long axis of that branch (Fig. 3A, C). The distribution of axis width frequencies was calculated based on measurements of all branching orders in each branching system, using PAST software and the optimal number of bins determined based on the zero-stage rule (Wand, 1997; Hammer, 2001). Specimens that do not bear branches were only measured for their basal width.

For axes bearing branches, distances between successive nodes (referred to as internodes hereafter) were grouped in four categories depending on the types of branches found at the nodes: (1) between developed branches; (2) between a developed branch and a dormant branch meristem; (3) between dormant branch meristems; and (4) between the base of a branch and a dormant branch meristem representing the lowermost branching point on that branch; the four categories of internodes were compared in terms of their length ranges.

For axes bearing sporangia, we measured the width at the level of sporangium attachment. For each sporangium we recorded the height and width, as well as its orientation relative to the subtending axis: longitudinal (sporangium width parallel to the subtending axis), longitudinal-oblique, transverse-oblique and transverse (sporangium width perpendicular to the length of the axis).

*Calculations*. The coefficient of branch subordination (CBS) quantifies the relative thickness of a branch (*i.j*) of an axis *i* with respect to the thickness of axis *i* at the branching point of *i.j*, where *j* denotes the position (node) of branch *i.j*. along axis *i*. CBS is thus calculated as the ratio between the basal width of branch *i.j* (*i.j*W_b_) and the width of the axis that subtends it at the branching point of *j* (*i*W*_i.j_*, Table 1); CBS = *i.j*W_b_/*i*W*_i.j_*. A CBS approaching 1 indicates isotomous branching, and the lower the CBS the more unequal (anisotomous) the branching is. CBS was calculated for branching points of developed branches, dormant branch meristems and underdeveloped branches with coiled tips (circinate branches).

**Table 1.** Descriptions and equations of the coefficients calculated for morphometric analyses of *Nowenia matsunagae*.

Coefficient	Description	Equation
Taper coefficient	The difference between the basal width of an axis *i* and its apical width (*i*W_b_ − *i*W_a_), divided by the total length between them (*i*L_b–a_)	(*i*W_b_ − *i*W_a_)/*i*L_b-a_
Coefficient of branch subordination (CBS)	The ratio between the basal width of branch *i.j* (*i.j*W_b_) and the width of the axis that subtends it at the branching point of *j* (*i*W*_i.j_*)	*i.j*W_b_/*i*W*_i.j_*
Tuft coefficient (TC)	The number of branching points in that branching system (*Ψ*) divided by the sum of the lengths of all axes in that branching system that bear branches (*ΣL*)	*Ψ*/*ΣL*

Branching density was characterized for each of the 16 most extensive specimens by calculating a tuft coefficient (TC), as the ratio of the total number of branching points in a specimen (*Ψ*) to the sum of the lengths of axes of all branching orders in that specimen that bear branches (*ΣL*): TC = *Ψ*/*ΣL* (Table 1). Higher TC values indicate denser branching and lower values correspond to sparser branching.

A taper coefficient was calculated for individual axes by subtracting the apical-most measurable width of an axis (*i*W_a_) from the basal-most measurable width of that axis (*i*W_b_) and dividing the difference by the length of axis between the two measurement points; taper = (*i*W_b_ − *i*W_a_)/*i*L_b–a_ (Table 1). Higher values of the taper coefficient correspond to axes with more pronounced taper.

*Material measured*. A total of 248 axis widths (the most basal complete width of each axis of all branching orders) were measured. Of these, the 16 specimens with the most extensive branching were measured for the cumulative length of all orders of branching (*ΣL*). The total number of axes (of all branching orders) measured for length is 88. The taper coefficient was calculated for 27 axes for which accurate apical width measurements could be taken >6.5 mm apart from the basal-most width measurement (because many specimens have incompletely preserved widths for significant portions of their length, and in order to minimize noise introduced by measuring error). A total of 45 branching points preserved complete basal widths of both main axis and branch and were measured for calculating the CBS. The length of 61 internodes between successive branching points was measured. A total of 32 sporangia were measured for height and/or width, depending on their state of preservation.

*Correlations*. To characterize the shape (proportions) of sporangia and to determine whether their shape changes as they grow to maturity, we checked for correlations between sporangium width and height. Additionally, to understand the growth dynamics of sporangia and axes together, we explored the correlations between axis width and sporangium size (width and height; the height and width of sporangia were considered separately because incomplete preservation allowed only one of the dimensions to be measured in some of the sporangia). This addresses whether more mature (larger) sporangia occur on older (thicker) axes. We also explored correlations between sporangium size (width and height) and orientation, which lets us know whether sporangia change orientation as they reach maturity; and between sporangium orientation and axis width, informative of whether sporangia change orientation as their subtending axes grow thicker.

**Fig. 4.**
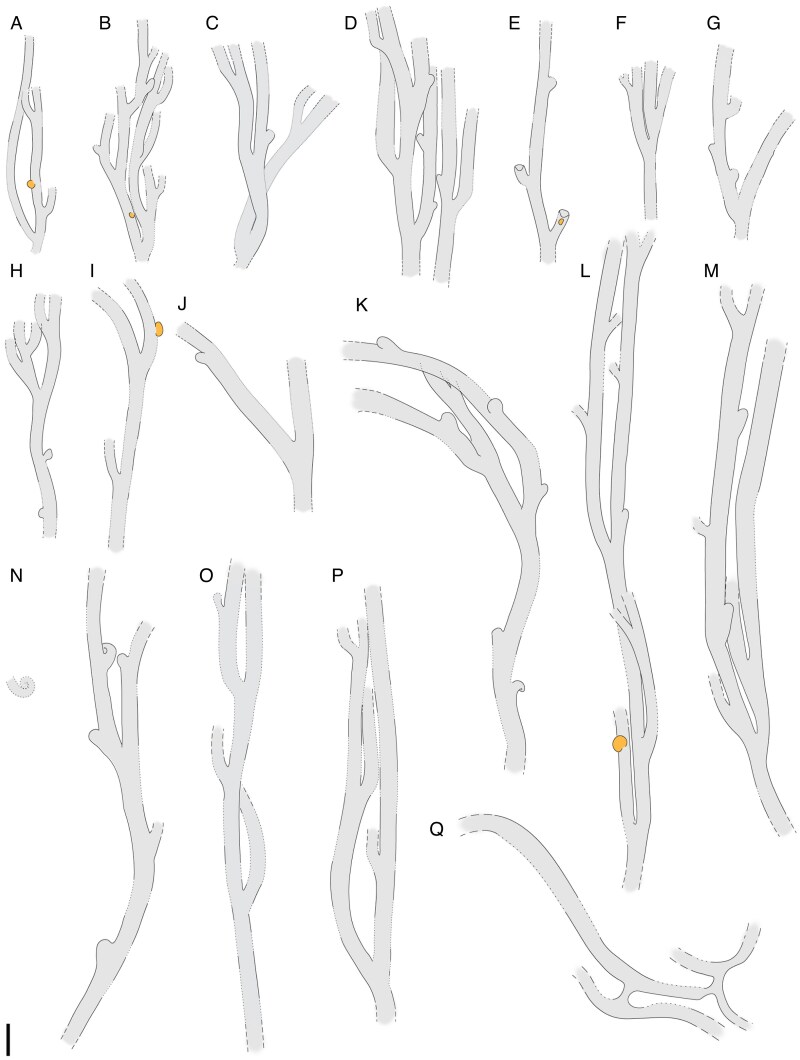
*Nowenia matsunagae* gen. et sp. nov. Range of morphological variation as reflected in tracings of the most extensive specimens (see also Fig. 5); ‘Tu’ in the descriptions below means tufted morphotype (PC1 score >0; see also Fig. 8); ‘De’ means decumbent morphotype (PC1 score <0); sporangia are shown in orange; specimens are arranged in decreasing order of PC1 scores from A to P; Q is the only specimen showing K-branching; solid lines represent preserved margins of the specimens, dotted lines mark missing portions. (A) HPH366 and counterpart HPH362. Tu: PC1 = 2.62. (B) HPH328 part and counterpart. Tu: PC1 = 2.50. (C) HPH386. Tu: PC1 = 1.12. (D) USNM598348 part and counterpart. Tu: PC1 = 1.09. (E) HPH662 and counterpart HPH671. Tu: PC1 = 1.08. (F) FM PP49078 part and counterpart. Tu: PC1 = 0.80. (G) HPH388. Tu: PC1 = 0.46. (H) KU D1526 and counterpart KU D1546. Tu: PC1 = 0.19. (I) KU D1515 part and counterpart. De: PC1 = −0.46. (J) FM PP16097. De: PC1 = −0.75. (K) FM PP15966. De: PC1 = −0.91. (L) HPH359 part and counterpart. De: PC1 = −1.10. (M) FM PP49079. De: PC1 = −1.13. (N) FM PP15956. De: PC1 = −1.19. (O) HPH361. De: PC1 = −1.67. (P) FM PP 49074. De: PC1 = −2.64. (Q) HPH705 and counterpart HPH707. Scale bar = 1 cm.

**Fig. 5.**
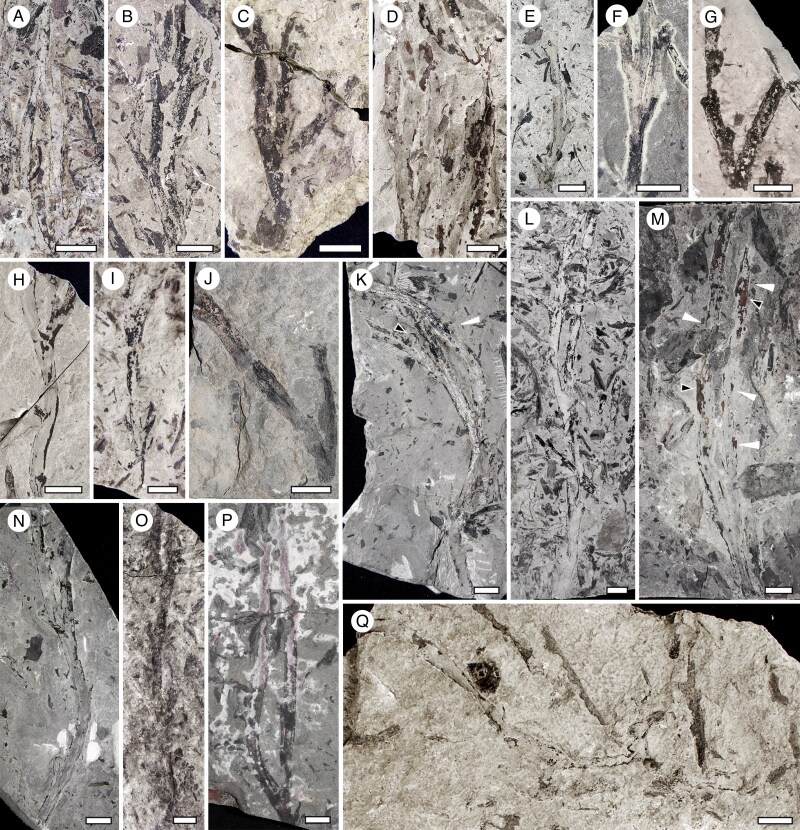
*Nowenia matsunagae* gen. et sp. nov. Specimens on which the tracings in Fig. 4 were produced, arranged in the same order as in Fig. 4 (white arrowheads were placed on collection specimens by previous researchers). (A) HPH366 (counterpart to HPH362). (B) HPH328. (C) HPH386 (see also Fig. 1A). (D) USNM598348. (E) HPH662 (counterpart to HPH671). (F) FM PP49078. (G) HPH388. (H) KU D1526 (counterpart to KU D1546). (I) KU D1515. (J) FM PP16097. (K) FM PP15966, note dormant branch bud (black arrowhead). (L) HPH359 (see also Fig. 2E). (M) FM PP49079, with large fragments of cuticle (black arrowheads). (N) FM PP15956 (see also Fig. 1G). (O) HPH361. (P) FM PP 49074 (see also Fig. 2D). (Q) HPH705 (counterpart to HPH707). Scale bars = 1 cm.

*Ordination*. Morphological variation amongst the 16 most extensive specimens (Figs 4 and 5) was analysed using a principal component analysis (PCA) based on a correlation matrix including the following variables: presence/absence of sporangia, the minimum distance between two successive developed branches, tuft coefficient and taper coefficient. The PCA was computed using the PAST software (Hammer, 2001).

### Phylogenetic analysis

The phylogenetic analysis was based on a matrix of 45 discrete morphological and anatomical characters developed for a broader zosterophyll phylogeny (Claisse *et al.*, 2025) (Supplementary Data Note). These characters were scored for the new zosterophyll described here and 13 other zosterophylls selected because they shared with the new zosterophyll a significant number of characters and based on the grouping of such taxa in the broader analysis by Claisse *et al.* (2025), which did not include the new zosterophyll (Supplementary Data Table S1). The taxa included in the analysis are *Crenaticaulis* (Banks and Davis, 1969), *Deheubarthia* (Edwards *et al.*, 1989), *Forania* (Jensen and Gensel, 2013), *Gosslingia* (Edwards, 1970), *Konioria* (Zdebska, 1982), *Odonax* (Gerrienne, 1996), *Oricilla* (Gensel, 1982), *Sawdonia* (Hueber, 1971), *Serrulacaulis* (Hueber and Banks, 1979), *Tarella* (Edwards and Kenrick, 1986), *Thrinkophyton* (Kenrick and Edwards, 1988), *Trichopherophyton* (Lyon and Edwards, 1991), which was used to root the tree, and *Zosterophyllum myretonianum* (Penhallow, 1892). Parsimony analyses were performed using TNT software, v. 1.5 (Goloboff and Catalano, 2016), using an exhaustive search with the command *ienum*.

## RESULTS

### Measurements (Supplementary Data Sheet)

The longest continuous specimen measures 19.1 cm along its main axis. Of the specimens with significant branching, the largest (Figs 4L and 5L) spans 16.5 cm. The most extensively branched specimen has four orders of branching (Figs 4B and 5B). The cumulative length of axes studied is 343.2 cm. Of this total, 226.5 cm corresponds to specimens assigned to a known morphotype: 159.6 cm to morphotype 1 and 66.9 cm to morphotype 2 (see below for morphotype descriptions).

**Fig. 6.**
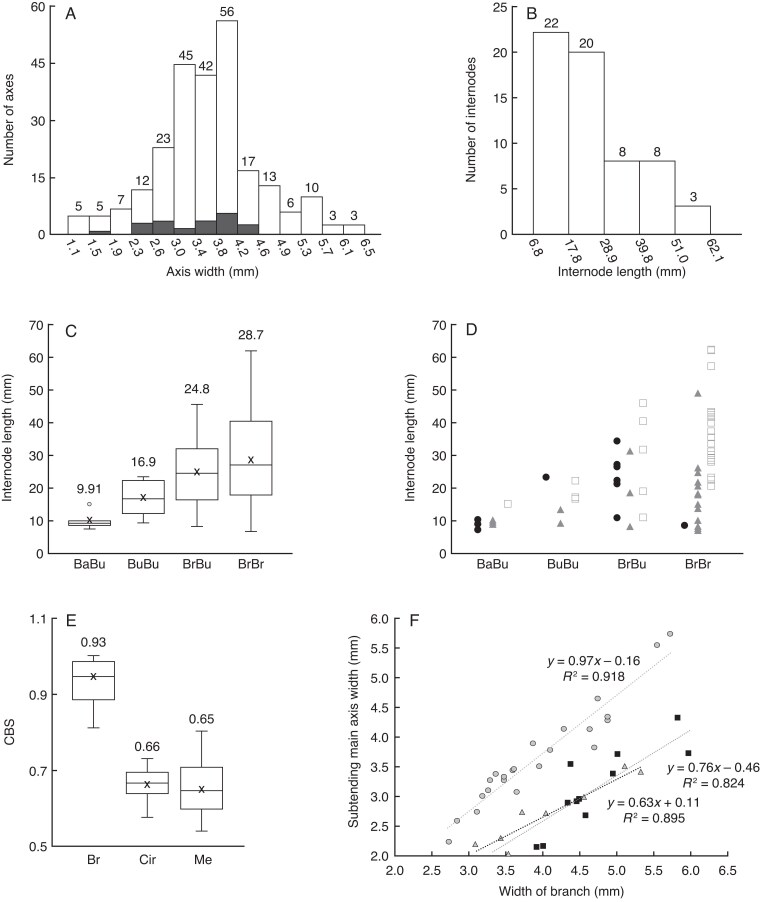
Morphometric data of *Nowenia matsunagae* gen. et sp. nov. (A) Axis width frequency distribution. More than half of axes are within the 2.6–4.2 mm range. Dark segments represent numbers of axes bearing sporangia in each width bin between 1.5 and 4.6 mm. (B) Internode length frequency distribution. More than two-thirds of internodes are shorter than 28.9 mm. (C) Internode length by branch category: BaBu, branch base to bud immediately distal to branch base, on that branch; BuBu, between two branch buds positioned at successive nodes; BrBu, developed branch to branch bud at successive nodes; BrBr, between two developed branches at successive nodes. Note that minima for all categories are similar. Horizontal lines in the boxes are medians, means are marked by x (and listed as the numbers above boxes). (D) Internode length by branch category separated by specimen morphotype (acronyms of internode types by branch category same as in C): grey triangles represent the tufted morphotype; grey squares represent the decumbent morphotype; black dots represent specimens not assigned to either of the two morphotypes; note that internodes of the decumbent morphotype range longer than those of the tufted morphotype. (E) Coefficient of branch subordination (CBS) by branch development category: Br, fully developed branches; Cir, circinate branches; Me, dormant branch meristems. Horizontal lines in the boxes are medians, means are marked by x (and listed as the numbers above boxes). (F) Correlations between basal width of branches and width of their subtending axes at the branching point, by branch development category. Underdeveloped branches are thinner than subtending axes: dormant branch meristems (black squares), *y* = 0.63*x* + 0.11, *r*^2^ = 0.895; circinate branches (grey triangles), *y* = 0.76*x* − 0.46, *r*^2^ = 0.824. Developed branches (black dots) are nearly equal in width to subtending axes, *y* = 0.97*x* − 0.16; *r*^2^ = 0.918.

Basal axis width (*i*W_b_) ranges from 1.2 to 6.5 mm (*x̅* = 3.7 mm) and has unimodal distribution, with a frequency maximum between widths of 1.2 and 6.5 mm (Fig. 6A). Axes that bear sporangia (*n* = 23) range in width from 1.8 to 4.4 mm (Fig. 6A).

The distance between successive branches (internode length) has a wide range of 6.9–62.0 mm (*x̅* = 24.5 mm, *n* = 61; Fig. 6B), but ∼65 % of internodes are <28.9 mm long. The minimum distances between successive branching points (internode lengths) are similar across all four branch categories: 7.5 mm for the distance between branch bases and dormant branch meristems that represent the lowermost branching points on those branches; 9.2 mm for the distance between successive branching points that are both dormant branch meristems; 8.2 mm for the distance between successive branching points that represent a developed branch and a dormant branch meristem; and 6.9 mm for the distance between successive branching points that each bear developed branches (Fig. 6C). The maximum distances between successive branching points vary widely between the four categories, as do the mean distances: 15.0 mm maximum for branch base to dormant branch meristems representing the lowermost branching point on that branch (*x̅* = 9.9 mm, *n* = 7), 23.2 mm maximum for dormant branch meristem to dormant branch meristem (*x̅* = 16.9 mm, *n* = 6), 40.2 mm maximum for developed branch to dormant branch meristem (*x̅* = 24.8 mm, *n* = 14), and 62.1 mm maximum for developed branch to developed branch (*x̅* = 28.7 mm, *n* = 34). Thus, the longest internodes are found between successive developed branches, and the smallest internodes are those between the base of a branch and the dormant branch meristem that is the lowest branching point on that branch.

We also used the internode length data (*n* = 61) to compare patterns of internode length by branch category in the two morphotypes distinguished by the PCA analysis (see below) (Fig. 6D). In general, for each branch category, internode lengths range into larger values with higher frequencies in specimens of morphotype 1 compared with specimens of morphotype 2. For developed branches, internode lengths range from 10.9 to 62.1 mm but most of them are >25 mm in morphotype 1, whereas in morphotype 2 they are generally <25 mm, even though they range from 6.8 to 48.6 mm. The larger internode lengths of morphotype 1 are consistent with their lower tuft coefficient (see Ordination section below).

Sporangia (*n* = 34; Supplementary Data Table S2), observed on 33 axis fragments, are slightly reniform or only lightly elliptical in shape, 1.5–4.2 mm wide (*x̅* = 2.7 mm, *n* = 31) and 0.9–3.7 mm tall (*x̅* = 1.9 mm, *n* = 31).

### Calculations

The CBS (Supplementary Data Table S3) ranges between 0.54 and 0.73 (*x̅* = 0.65; *n* = 13) for dormant branch meristems, between 0.57 and 0.73 (*x̅* = 0.66; *n* = 8) for underdeveloped circinate branches, and between 0.81 and 1.00 (*x̅* = 0.93, *n* = 24) for developed branches (Fig. 6E). Thus, developed branches are close to equal in width to their subtending axes (*y* = 0.9717*x −* 0.1552; *r*^2^ = 0.918), whereas dormant branch meristems (*y* = 0.7617*x −* 0.4635; *r*^2^ = 0.824) and circinate branches (*y* = 0.6347*x −* 0.1111; *r*^2^ = 0.895) are smaller in basal width than their subtending axes (Fig. 6F).

**Fig. 7.**
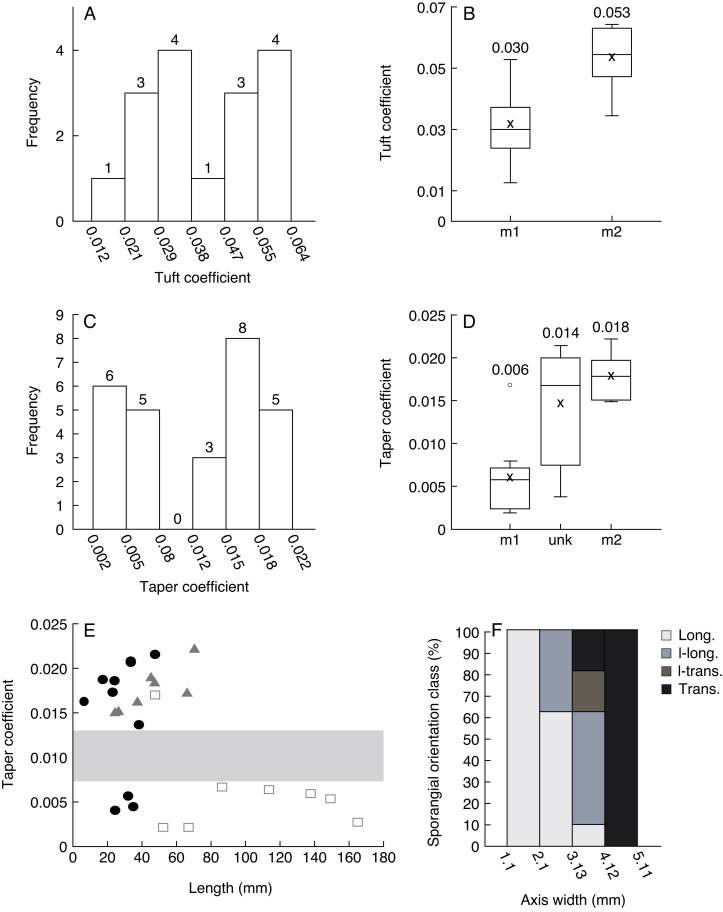
Morphometric data of *Nowenia matsunagae* gen. et sp. nov. (A, B) Tuft coefficients calculated from the 16 most extensive specimens (see Figs 4 and 5): frequency distribution is bimodal (A) and the ranges of decumbent specimens (m1) overlap only partially with those of tufted specimens (m2) (B). Horizontal lines in the boxes are medians, means are marked by x (and listed as the numbers above boxes). (C–E) Taper coefficients calculated from the 16 most extensive specimens and additional specimens that provided relevant data (see Figs 4 and 5). Frequency distribution is bimodal, with a gap (C); there is no overlap in taper coefficients between decumbent (m1) and tufted specimens (m2), while specimens not assignable to either morphotype (unk) overlap in range with both morphotypes (D); horizontal lines in the boxes are medians; means are marked by x (and listed as the numbers above boxes); decumbent specimens (white squares) are preserved as longer fragments and have taper <0.008 (except for one specimen), whereas tufted specimens (grey triangles) are preserved as smaller fragments and have taper coefficients >0.012, and no unassignable specimens (black dots) fall within the 0.008–0.012 taper coefficient gap (grey band in E). (F) Frequency distribution of sporangium orientation classes by axis width; sporangia are oriented exclusively longitudinally (Long) on the thinner axes, exclusively transversely (Trans) on the thicker axes, and sporangia on axes of intermediate sizes have intermediate orientations (I-long and I-trans).

The tuft coefficient, calculated for the 16 most extensive specimens (Supplementary Data Table S4), ranges from 0.012 to 0.064 mm^−1^ (*x̅* = 0.040 mm^−1^; *n* = 16) and has a bimodal distribution, with maxima in the 0.030–0.038 mm^−1^ and 0.056–0.064 mm^−1^ intervals (Fig. 7A, B).

The taper coefficient ranges from 0.002 to 0.022 (*x̅* = 0.013, *n* = 27) and has markedly bimodal distribution, with peaks at 0.002–0.005 and 0.015–0.019, respectively. No specimens have taper coefficients between 0.008 and 0.012 (Fig. 7C). When compared for the two morphotypes distinguished by the PCA analysis (see Ordination below), taper coefficients (Fig. 7D; Supplementary Data Table S4) show a clear dichotomy between morphotype 2, with higher taper coefficients (0.015–0.022; *x̅* = 0.018, *n* = 6), and morphotype 1 (0.002–0.016; *x̅* = 0.006, *n* = 9). The 12 specimens that were not included in the PCA analysis (because of their smaller size and number of branching points) and, as a result, were not assigned a priori to a morphotype, can be tentatively assigned to one of the two morphotypes based on their taper coefficients (Fig. 7D). The disjunct ranges of taper coefficients in the two morphotypes are also apparent when taper is plotted against axis length for the same specimens (Fig. 7E). Morphotype 1, with lower taper coefficients, also includes the largest preserved specimens, and the separation of specimens not assigned to either of the two morphotypes is also clear in this representation.

### Correlations

An analysis of the relationship between internode length and axis width at the base of that internode, measured for all internodes between two preserved branching points (*n* = 35) in 17 specimens, showed absence of linear correlation (*r*^2^ = 0.096).

The relationship between sporangium width and height (Supplementary Data Table S2) shows weak linear correlation (*r*^2^ = 0.577; *n* = 12), which is not surprising since sporangium shape approaches a circular shape. We found no correlation between either the width or height of sporangia and the width of the axes that subtend them: *r*^2^ = 0.004 (*n* = 14) for sporangium width and *r*^2^ = 0.110 (*n* = 17) for sporangium height. We also found no consistent pattern of relationship between sporangium orientation and size. Nevertheless, a pattern emerges in the correlation between sporangium orientation and axis size (Supplementary Data Table S2): whereas thicker axes generally bear more transversely oriented sporangia, thinner axes bear more longitudinally oriented sporangia (Fig. 7F).

### Ordination

**Fig. 8.**
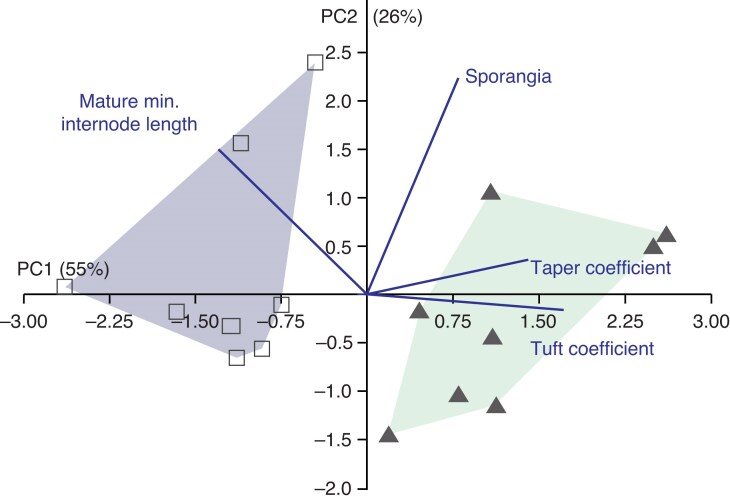
Principal component analysis of *Nowenia matsunagae* gen. et sp. nov. morphology based on four characters (Supplementary Data Table S4) and including the 16 most extensive specimens (see Figs 4 and 5). PC1 scores separate specimens of the decumbent morphotype (squares) from those of the tufted morphotype (triangles). The analysis shows positive correlation between taper coefficient and tuft coefficient and between tuft coefficient and presence of sporangia, and negative correlation between both taper coefficient and tuft coefficient, on one hand, and the minimum internode length between two developed branches, on the other hand.

The PCA (Fig. 8, Supplementary Data Tables S4–S6) performed on the 16 most extensive specimens (Figs 4 and 5) using four variables, identified two principal component (PC) axes that together describe 73.44 of the variation in the dataset (PC1, 49.36 %; PC2, 24.08 %). The PCA shows (1) positive correlation between taper coefficient and tuft coefficient; (2) weak positive correlation between tuft coefficient and presence of sporangia; and (3) negative correlation between both taper coefficient and tuft coefficient, on one hand, and minimum internode length between two successive developed branches, on the other hand (Fig. 8). Additionally, specimens with lower tuft coefficients have negative PC1 values and are less likely to have sporangia than those with positive PC1 values, which have higher tuft coefficients and bear the majority of sporangia identified (Fig. 8). Because PC 1 records a strong distinction between these two types of morphology, the specimens with a negative PC1 value will be referred to hereafter as ‘decumbent morphotype’ (morphotype 1), while those with a positive PC1 value will be referred to as ‘tufted morphotype’ (morphotype 2) (Fig. 8); see also Description below.

### Systematics

**Division**: Tracheophyta Cavalier-Smith (1998)

**Subdivision**: Lycophytina Kenrick and Crane (1997)

**Genus**: *Nowenia* El-Abdallah et Tomescu gen. nov.

**Generic diagnosis**: Plant with smooth axes branching at low angles, branches curved apically close to their base and parallel to subtending axis. Axis tips circinate. Branching system comprising two distinct morphologies: densely branched axes, probably upright, and more sparsely branched axes, probably creeping. K-branching present, subaxillary tubercles or branches absent. Sporangia solitary, attached laterally on axes, bivalvate, dehiscing along distal and lateral margin. Spores trilete.

**Etymology**: ‘nowen’, the English pronunciation of the Standard Arabic word ‘نوعين’ meaning ‘two types’ or ‘two kinds’, recognizes the two different morphologies that make up the plant, as well as the Middle Eastern heritage passed on to the first author by her father, who supported her throughout her studies but passed away during completion of this work.

**Type species**: *Nowenia matsunagae* El-Abdallah et Tomescu sp. nov.

**Specific diagnosis**: As in generic diagnosis. Axes up to 6.5 mm wide, including at least four orders of branching and often imprinted with fine, long-sinuous lines. Branches in alternate arrangement, some of them immature at different developmental stages, from dormant meristems forming small lateral protrusions to short branches adaxially circinate to various degrees. Fully developed branches comparable in thickness to the subtending axes. Densely branched portions with successive branches 6–48 mm apart, taper >1/10 000, probably upright. Sparsely branched portions with successive branches 10–62 mm apart, taper <1/10 000, probably creeping. K-branching portions ∼5 mm thick. Epidermal cells polygonal, isodiametric to slightly elongated longitudinally, up to 115 µm, some bearing cuticular thickenings in a small central area and radiating ridges. Epidermal cells occasionally forming rosettes around circular cells ∼50 µm in diameter. Stomatal guard cells reniform, 95 × 30 µm. Sporangia reniform to elliptical, up to 3.7 × 4.2 mm at maturity, dorsiventrally flattened, equivalvate with smooth valves and lacking marked thickening along dehiscence line. Immature sporangia in apical croziers oriented with mediolateral plane parallel to the subtending axis. Mature sporangia on very short stout stalks, pointing apically, with mediolateral plane perpendicular to the subtending axis. Spores trilete, unornamented, 36.3–44.5 µm diameter.

**Fig. 9.**
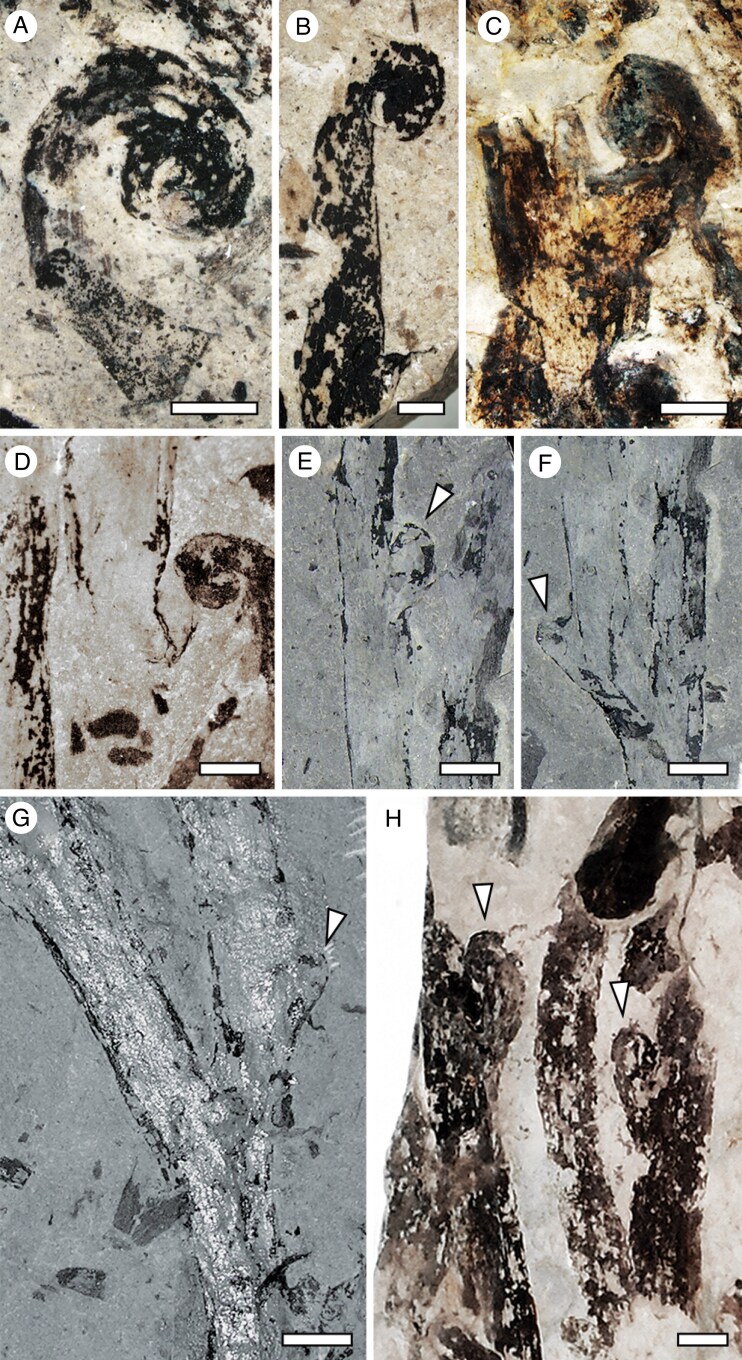
*Nowenia matsunagae* gen. et sp. nov. (A, B) Circinate apices of axes of varying morphologies. (A) Circinate apex forming two or three coils. HPH334. (B) Hook-like circinate apex. HPH665. Scale bars = 2 mm. (C, D) Underdeveloped branches with circinate apices. (C) HPH638. (D). HPH359. Scale bars = 2 mm. (E, F) Knob-like dormant branch buds of varying positions (arrowheads). (E) Dormant branch buds are present in the position of branches. (F) Dormant branch buds are found on branches, sometimes very close to the base of the branch, in which case they may represent a dormant side of a K-branch. FM PP15956. Scale bars = 5 mm. (G) Dormant branch bud with knob-like morphology (arrowhead), on first order branch, very close to base (possibly a dormant side of a K-branch). FM PP49075. Scale bar = 5 mm. (H) Elongated branch buds (arrowheads); note bud at left beginning to show a coiled tip. HPH541. Scale bar = 5 mm.

**Holotype**: HPH359 (Figs 2E, 4L, 5L and 9D).

**Fig. 10.**
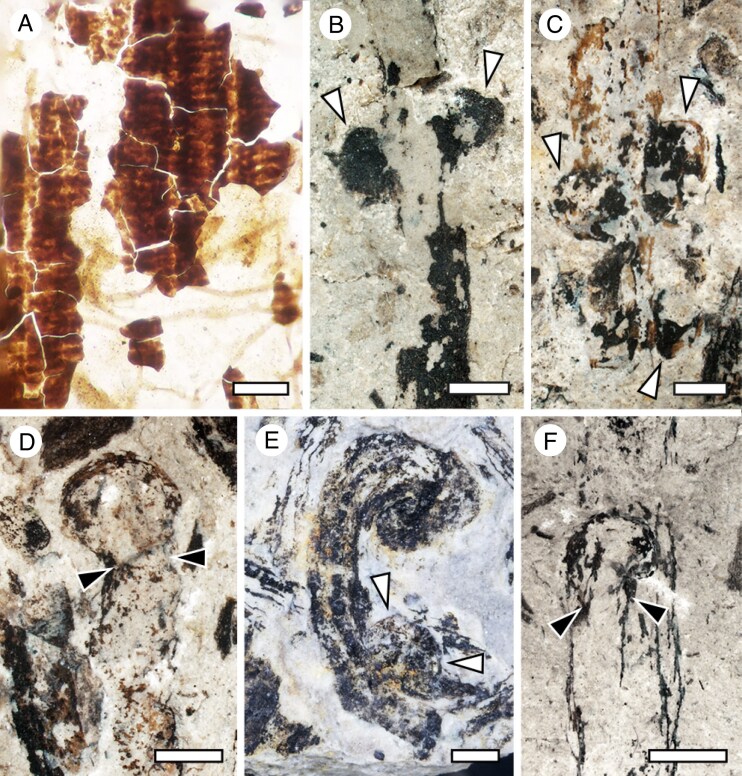
*Nowenia matsunagae* gen. et sp. nov. (A) Tracheids recovered on an acetate peel. Secondary wall thickenings form a scalariform pattern but detailed tracheid structure (possible *Gosslingia*-type thickenings) cannot be resolved. Scale bar = 30 µm. HPH541. (B) Axis bearing two sub-opposite sporangia (arrowheads); sporangial stalks not visible. Scale bar = 2 mm. KS D1515a. (C) Axis bearing three sporangia (arrowheads), one of which (upper right) is preserved with a distal line of dehiscence (see details of two top sporangia in Fig. 11A–D). Scale bar = 2 mm. HPH334. (D) Sporangium oriented transversely with respect to subtending axis; axis section above sporangium is covered by rock matrix. Note fine cuticle preserved along the distal line of dehiscence at top; arrowheads point to the base of the short sporangial stalk. Scale bar = 2 mm. HPH369. (E) Sporangium with longitudinal orientation (between arrowheads) borne immediately beneath a circinate axis tip. Scale bar = 1 mm. HPH465. (F) Reniform sporangium oriented transversely with respect to subtending axis; arrowheads point to the base of the short sporangial stalk; axis section above sporangium is covered by rock matrix. Scale bar = 3 mm. HPH407.

**Fig. 11.**
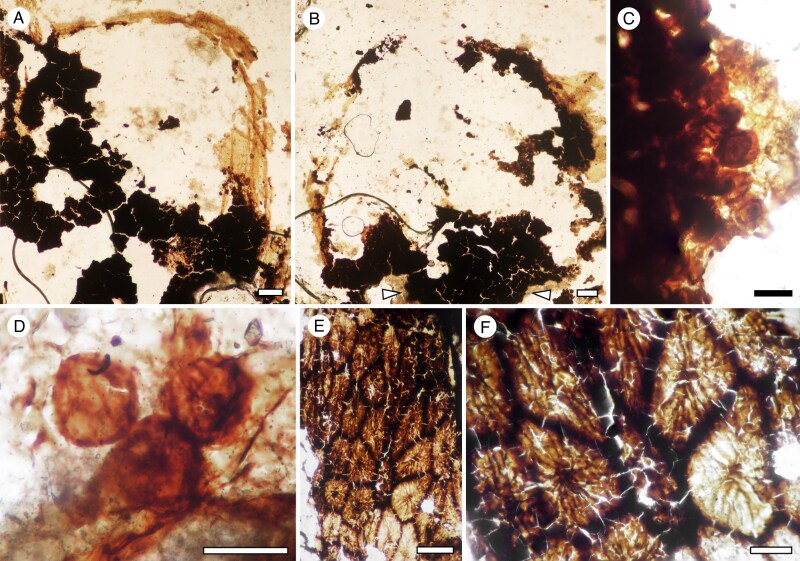
*Nowenia matsunagae* gen. et sp. nov. (A–D) Sporangia and spores recovered on acetate peels (see also Fig. 10C). Slide HPH334-1. (A, B) Sporangial wall is thick (dark material with cracked pattern; bottom and left in (A), bottom and around sporangium edge in (B)) but without additional thickening in the dehiscence area; note light-coloured fine cuticle marking the dehiscence area in (A) and the semicircular–reniform shape of the sporangium and wide sporangial stalk (between arrowheads) in (B). Scale bars = 200 µm. (C, D) Unornamented spores preserved in the dehiscence area of the sporangia. Scale bars = 40 µm. (E, F) Epidermal cells of axis recovered on acetate peel. Note central dark area (papilla) on cuticle of each cell and ridges radiating from it. Slide HPH360-3. Scale bar = 100 µm (E), 40 µm (F).

**Fig. 12.**
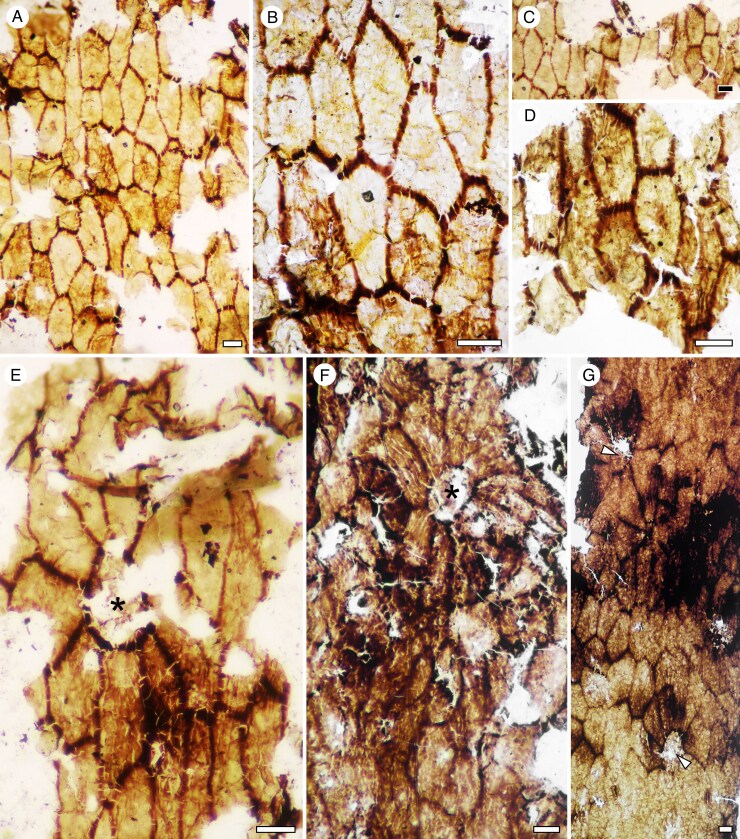
*Nowenia matsunagae* gen. et sp. nov. epidermal cell patterns on cuticle recovered in acetate peels. (A–D) Epidermal cells are angular and slightly elongated. The amount of sculpturing (central papillae and radiating ridges; see Fig. 11E, F) varies from absent to weakly expressed. Slide HPH360-3. Scale bars = 40 µm. (E–G) Epidermal cells are often arranged in rosettes. Slightly elongated angular cells form rosettes around oval central cells, marked by asterisk or arrowheads (E, slide HPH360-3; F, slide HPH774-2; G, slide USNM598348-1). Note central cells may lack parts of the cuticle and the outlines of epidermal cells may be more or less marked, even across small areas of the epidermis (compare central vs top and bottom parts in (G)). Scale bars = 40 µm.

**Fig. 13.**
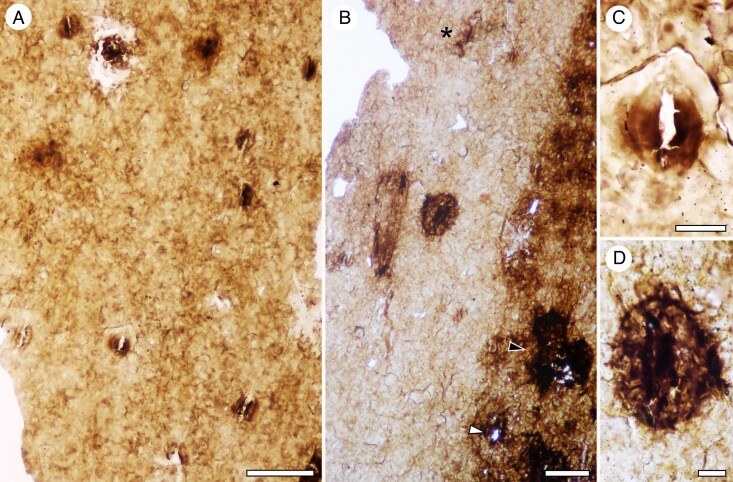
*Nowenia matsunagae* gen. et sp. nov. Epidermal patterns on cuticle recovered in acetate peels. (A) Numerous stomata on cuticle fragment; note indistinct cellular pattern of the cuticle. Slide USNM598348-13. Scale bar = 100 µm. (B) Stoma, at centre, with conspicuous guard cells in a cuticle fragment with indistinct cellular pattern; note closed stoma (at top, to the right of asterisk), open stoma (white arrowhead) and group of darkened cells (black arrowhead) that may represent necrosis in response to herbivory. Slide USNM598348-5. Scale bar = 100 µm. (C) Detail of (A). Stoma bordered by darker areas of thicker material similar to the structures referred to as cuticular rims or inner poral thickenings by Sun *et al.* (2005). Scale bar = 20 µm. (D) Detail of (B). Scale bar = 20 µm.

**Fig. 14.**
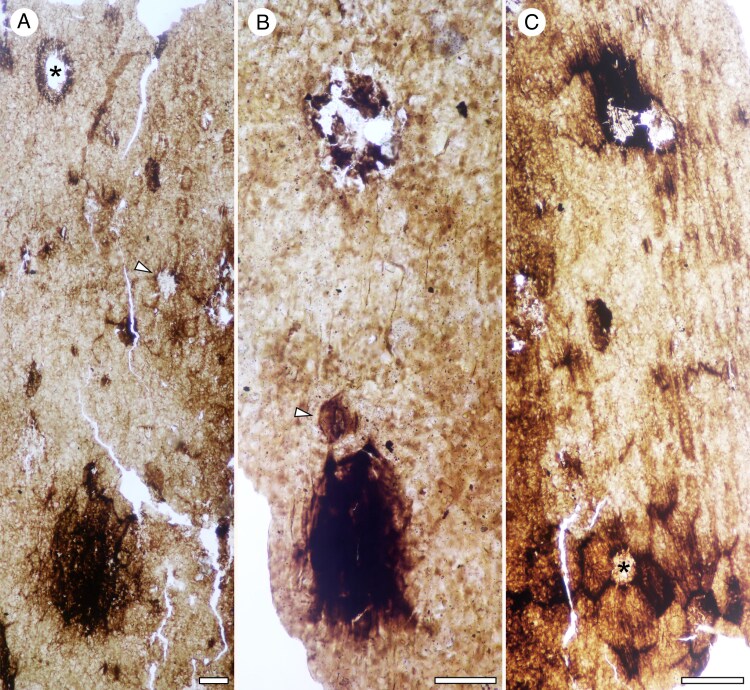
*Nowenia matsunagae* gen. et sp. nov. Putative traces of herbivory on cuticle recovered in acetate peels. Areas representing putative traces of herbivory consist of groups of darkened cells (at bottom in (A) and (B)), areas of missing cuticle with darkened edges (asterisk in (A)) or areas of missing cuticle with partial darkened fragments or cells (at top in (B) and (C)). Note rosette patterns in (A) (arrowhead) and (C) (asterisk), and stoma in (B) (arrowhead). (A) Slide USNM598348-3, scale bar = 100 µm; (B) slide USNM598348-16, scale bar = 50 µm; (C) slide USNM598348-6, scale bar = 150 µm.

**Paratypes**: HPH317 (Fig. 1B, Supplementary Data Fig. S2C); HPH 328 (Figs 4B and 5B); HPH334 (Figs 9A, 10C and 11A, C, D); HPH360 (Figs 11E, F and 12A–E); HPH361 (Figs 4O and 5O); HPH 362 (counterpart to HPH366) (Figs 4A and 5A); HPH 369 (Fig. 10D); HPH386 (Figs 1A, 4C and 5C); HPH388 (Figs 4G and 5G); HPH407 (Fig. 10F); HPH465 (Fig. 10E); HPH541 (Figs 1C, 9H and 10A, Supplementary Data Fig. S1A); HPH581 (Supplementary Data Fig. S1B); HPH638 (Fig. 9C); HPH662 (counterpart to HPH671) (Figs 4E and 5E); HPH665 (Fig. 9B); HPH705 (counterpart to HPH707) (Figs 4Q and 5Q); HPH774 (Fig. 12F); HPH792 (Fig. 1F); KS D1515 (Figs 4I and 5I); KS D1541b (Supplementary Data Fig. S2A, C); KS D1546 (counterpart to KS D1526) (Figs 4H and 5H); KS D1588a (Figs 1D, E and 2A–C, Supplementary Data Fig. S1E); KS D1588b (Supplementary Data Fig. S1D); KS D1588d (Supplementary Data Fig. S1C); FM PP15956 (Figs 1G, 4N, 5N and 9E, F); FM PP15966 (Figs 4K and 5K); FM PP16097 (Figs 4J and 5J); FM PP49079 (Figs 4M and 5M); FM PP49074 (Figs 2E, 4P and 5P); FM PP49075 (Fig. 9G); FM PP49078 (Figs 4F and 5F); USNM 598348 (Figs 4D, 5D, 12G, 13 and 14).

**Locality**: South wall of Cottonwood Canyon (Big Horn County, Wyoming), 44°51′51″N, 108°02′46″W.

**Stratigraphic position and age**: Beartooth Butte Formation, Early Devonian: late Lochkovian to early Pragian.

**Etymology**: In recognition of Kelly K. S. Matsunaga’s contributions to developing methods for morphometrics-informed whole-plant reconstructions of Early Devonian lycophytes.

### Description

*Types of preservation and taphonomy.* Branching systems of *Nowenia matsunagae* (consisting of up to four orders of branching and up to ten total branches) are preserved as adpressions that fall into three categories. The majority of specimens are preserved as solid black coalified compressions (Fig. 1A). Some compressions of this type have lost variable amounts of the coaly material, leaving behind impressions mottled with patches of coaly material (Fig. 1B). A second category consists of compressions that exhibit differentiation of a dark central strand flanked by lighter-coloured coaly material that often has a yellow iridescent aspect. The light-coloured material is cuticle that can preserve cellular patterns and may show evidence of oxidation. The dark central strand, representing the conducting tissues (Fig. 1C), is conspicuous in ∼12 % of specimens. The third preservation category includes axes preserved as coalified compressions of uneven width that bear longitudinal sinuous creases (Fig. 2A, B). Specimens with this mode of preservation occasionally show oxidation (Fig. 2C) and do not have a visible vascular strand. They are mostly found in monotypic associations on bedding planes, as intertangled mats with axes sometimes exhibiting preferential orientation (Fig. 2D).

Most *Nowenia matsunagae* specimens are not found preserved alongside other plant taxa: only 34 (39 %) out of 87 rock samples containing *Nowenia* axes also hold other plants (Supplementary Data Sheet). In 19 of these 34 samples (∼56 %) *Nowenia* co-occurs with *Sengelia radicans* (Matsunaga and Tomescu, 2017). *Nowenia* axes are found alongside fish fossils in 24 (∼28 %) of the total rock specimens. Microconchids (encrusting lophophorates; Taylor and Vinn, 2006; Vinn, 2006; Caruso and Tomescu, 2012) are found on 80 *Nowenia* axes (Supplementary Data Sheet; Supplementary Data Fig. S3A, B).

*General morphology of axes.* The axes of *Nowenia* are naked. They range from 1.15 to 6.52 mm in width and taper varies from 0.2 to 2.2 mm of width per 10 cm of length. Axis apices are circinate, varying from a few coils to barely hook-like tips (Fig. 9A, B). The thickness of axes in the coiled apices ranges from 1.2 to 3.8 mm.

Branches diverge at relatively narrow angles and curve apically close to the branching point, becoming more or less parallel to their subtending axes and producing a characteristic morphology (referred to as U-shaped or U-patterned branching hereafter; scored as ‘Branch laterals run parallel to main axis’ in Nibbelink and Tomescu, 2022; Figs 2D, 2E, 4 and 5). Branch primordia, possibly dormant meristems, appear knob-like or are circinate to different degrees (Fig. 9C–H), similar in structure to the circinate axis apices. Other branches are preserved mid-development, as the circinate apex uncoils as the branch increases in length and thickness (Fig. 9C–E). Branch primordia occur on all branching orders and can sometimes be found close to the base of branches (∼1–2 cm from the base; Fig. 9F, G). Fully developed branches are equal or nearly equal in thickness to their subtending axes (Fig. 6F).

Internode length varies widely (Fig. 6B–E): internodes between adjacent developed branches are 6.9–62.1 mm (Fig. 6B). Across this wide range, some portions of the plant appear more densely branched and tuft-like, while others branch more sparsely with longer internodes (Fig. 7A), consistent with the separation of two branching habits revealed by the ordination analysis – the decumbent vs tufted morphotypes (Fig. 8). Specimens exhibiting K-branching are present (Figs 4Q and 5Q).

When separated from the rock, the thin (∼1 mm) vascular strand visible in some of the specimens is sub-optimally preserved but occasionally exhibits an overall scalariform pattern (Fig. 10A) that is consistent with *Gosslingia*-type (G-type) thickenings (Kenrick and Crane, 1991) characteristic of zosterophylls.

*Variation in axis morphology encompasses three conspecific morphotypes.* Two distinct morphotypes have been separated based on features of the 16 most extensive specimens: specimens that taper less and branch more sparsely are less likely to have sporangia, whereas specimens that taper to a greater degree and branch more densely are more likely to possess sporangia (see results in the Ordination section above; Fig. 8, Supplementary Data Tables S4–6). The other 232 specimens observed are too fragmentary to be assigned to one of the two morphotypes, although measurements taken on them contribute to documenting variation in other aspects of the morphology of *Nowenia*. A third morphotype is based on a single specimen.

(1) Decumbent morphotype. Axes of this morphotype have sparse branching with long internodes (10.9–62.1 mm, *x̅* = 33.1 mm; Fig. 6D) and an overall lanky appearance (Figs 4I–P and 5I–P). These axes can be thicker, do not taper as much as axes of the tufted morphotype (taper 0.2–0.8 mm per 10 cm of length; Fig. 7D), and tend to be preserved as longer specimens (Fig. 7E) that are not very spread out laterally. The most extensive specimen (16.5 cm in a straight line between its two farthest extremities) has two orders of branching with a total of nine branches (Figs 4L and 5L). This specimen is also the only one of its morphotype to possess a sporangium, borne on one of its first order branches.

(2) Tufted morphotype. Axes of this morphotype have internodes ranging from 6.9 to 48.6 mm in length (*x̅* = 17.3 mm; Fig. 6D). The most extensive specimen in this morphotype has ten branches in four orders of branching, within a total extent (between its two farthest extremities) of only 6.7 cm (Figs 4B and 5B). The axes have higher taper values (1.5–2.2 mm per 10 cm of length; Fig. 7D). The overall denser branching makes for a bushy appearance that fans out from the base into the successive orders of branching (Figs 4A–H and 5A–H). Three branching systems of this morphotype bear sporangia.

(3) K-branching. One specimen exhibits H- or K-branching typically associated with a rhizomatous habit in some plants (e.g. Matsunaga and Tomescu, 2017; Poschmann *et al.*, 2020). The specimen (Figs 4Q and 5Q) has two K-branching points, where short (3–6 mm) branches diverge perpendicularly to the main axis and dichotomize to form two branches that diverge in opposite directions from one another and approximately parallel to the subtending axis. The segments of this specimen have similar widths, ∼5.2 mm. The K-branching of this specimen is reiterative, as the second K-branching is formed on one of the branches produced by the first K-branching. This specimen is considered conspecific with the other remains of *Nowenia* based on its similar preservation mode, texture of the coaly material and fine, long-sinuous creases, as well as its distinctiveness from K-branching specimens assigned to other taxa in the Cottonwood Canyon assemblages (see last heading under Remarks below).

*Sporangia and spores.* Sporangia (Figs 10B–F and 11A, B) are laterally attached to the axes by wide (∼1.7 mm) and very short (<0.5 mm) stalks (Fig. 10F) that are not always visible (Fig. 10B–E). They typically occur isolated, but one axis preserves two sporangia in close proximity to each other (Fig. 10B) and another one preserves three closely spaced sporangia (Fig. 10C). The sporangia are reniform to elliptical, as wide as 4.2 mm and as tall as 3.7 mm. They were probably equivalvate and lack a markedly thickened line of dehiscence. A few of them reveal, instead, a fine line of dehiscence that runs along the distal and lateral margins (Figs 10D and 11A). The sporangia are usually found on thinner axes (1.75–4.40 mm in thickness) and are also found in early developmental stages in apical coils (Fig. 10E). On the thicker axes, sporangia are attached transversely (Fig. 10D, F), but thinner axes and coiled apices tend to subtend longitudinally attached sporangia (Fig. 10E; see also Fig. 7F).

Cuticle recovered from sporangia suggests that the sporangium wall is thinner at the edges of the two valves bordering the dehiscence line and thicker away from the edges (Fig. 11A, B). Cellular patterns of the sporangium wall are not preserved, but numerous spores can be seen inside and around the dehiscence areas of two sporangia (Fig. 11C, D). The spores are trilete, 36.3–44.5 µm in size, and their walls are devoid of sculpturing (Fig. 11D).

*Epidermal features observed in cuticular material.* Cells of the epidermis vary in size, shape and surface features (Figs 11E, F and 12). Cell shape varies from approximately isodiametric (Figs 11E, F and 12) to more often elongated longitudinally (Fig. 12). Cell size varies from 46.6–75.9 µm for the more isodiametric cells to 34.9 × 116.5 to 53.7 × 146.3 µm for the elongated ones. Some epidermal cells show sculpturing (Fig. 11E, F), while others seem to lack sculpturing (Fig. 12A–C) and some present intermediate forms (Fig. 12C, D). The sculpturing, probably representing areas of thickened cuticle, consists of a small, darker central area and ridges that radiate from it in all directions to the edges of the cell (Fig. 11E, F), similar to the cellular sculpturing documented in *Sawdonia ornata* by Edwards *et al.* (1982) and Rayner (1983) and in *Forania plegiospinosa* by Jensen and Gensel (2013). Some epidermal cells are grouped in rosette patterns formed by larger angular and slightly elongated cells that surround a round to oval central cell (Fig. 12E–G). These patterns are similar to those described in the epidermis of other zosterophylls (see last heading under Remarks below).

Stomata are numerous, oriented longitudinally (Fig. 13A). Most of the stomata are represented on cuticular material only by slit-shaped openings bordered by two narrower or broader darker areas of thicker material (Fig. 13A–C). These are similar to the stomatal features referred to as cuticular rims or inner poral thickenings by Sun *et al.* (2005). In rare cases, the entire guard cells can be seen (Fig. 13B, D); they are ∼95 µm long and 29.0–32.5 µm wide.

*Putative evidence of herbivory.* Some of the cuticular fragments exhibit features interpreted as traces of herbivory (Fig. 14). Some of these consist of relatively small areas typically oval in shape where the cuticular material is very dark, with no cellular detail visible (Fig. 14A, B). Other such areas are suggestive of lesions; they have a dark border around an area where the cuticular material is missing (Fig. 14A). In other cases, the cuticle in those areas has amorphous, patchy dark material (Fig. 14B) or light-coloured material with unclear cellular features (Fig. 14C).

### Phylogeny

**Fig. 15.**
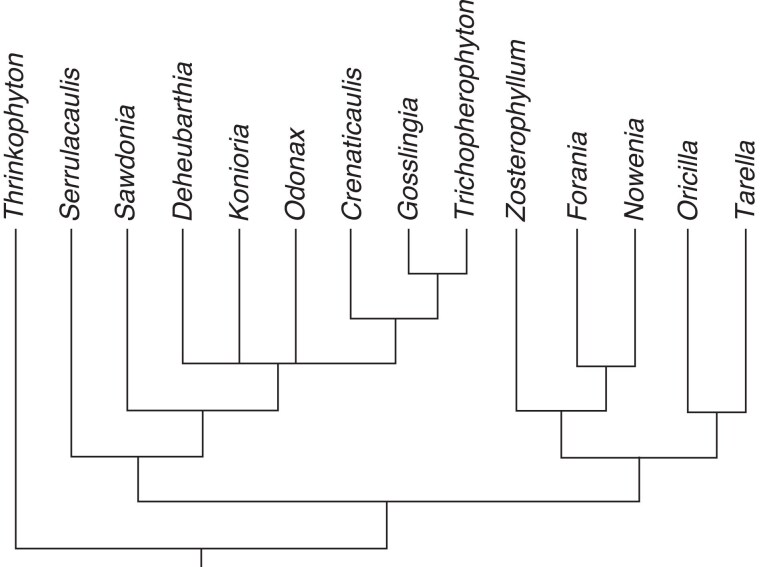
Phylogenetic placement of *Nowenia* based on a parsimony-constrained analysis (data from Claisse *et al.*, 2025; Supplementary Data Table S1). *Zosterophyllum* = *Z. myretonianum*.

The parsimony-constrained phylogenetic analysis found three most parsimonious trees (Length = 54; Consistency Index = 0.630; Retention Index = 0.623) whose consensus placed *Nowenia* sister to *Forania plegiospinosa* (Fig. 15) in a clade supported by shared sporangium shape and orientation, and by absence of emergences on the sporangia; *Zosterophyllum myretonianum* is sister to the clade formed by *Nowenia* and *Forania*. A clade formed by *Oricilla* and *Tarella* is sister to the *Zosterophyllum–Forania–Nowenia* clade; the two clades share dorsiventrally flattened sporangia as a synapomorphy. The other eight zosterophyll genera included in the analysis form a separate clade.

### Remarks

*Taxonomic placement – comparison with similar taxa and justification of a new genus (see Supplementary Data Note for extended version).* Taken together, the phylogenetic position of the new plant and the morphological comparisons discussed below justify the erection of a new zosterophyll genus.

An exarch protostele, a diagnostic character of zosterophylls (Banks, 1968), cannot be ascertained in *Nowenia*, due to its preservation as adpressions. However, *Nowenia* possesses reniform sporangia attached laterally on axes and dehiscing along their distal margin, two other diagnostic features of zosterophylls (Banks, 1968), which support its placement in class Zosterophyllopsida (*sensu*Kenrick and Crane, 1997). *Nowenia* possesses additional features documented in zosterophylls and not usually seen in other groups of early tracheophytes: K- or H-branching and rosette cellular patterns in the epidermis (Edwards *et al.*, 1982; Gensel and Andrews, 1984; Gensel, 1992; Kenrick and Crane, 1997).

Phylogenetic analyses place *Nowenia* in a clade with 13 other zosterophyll genera (Fig. 15, Supplementary Data Table S1). Of these, *Trichopherophyton* (Lyon and Edwards, 1991), *Crenaticaulis* (Banks and Davis, 1969), *Deheubarthia* (Edwards *et al.*, 1989), *Gosslingia* (Edwards, 1970), *Odonax* (Gerrienne, 1996), *Thrinkophyton* (Kenrick and Edwards, 1988), *Sawdonia* (Hueber, 1971; Gensel *et al.*, 1975; Rayner, 1983; Gensel and Berry, 2016), *Serrulacaulis* (Hueber and Banks, 1979; Berry and Edwards, 1994; Xu, 2011) and *Konioria* (Zdebska, 1982) either lack morphological information relevant to detailed comparisons or possess diagnostic features that are absent in *Nowenia* and can be excluded from further taxonomic discussions (Supplementary Data Table S1; Supplementary Data Note). The close relationships supported by the phylogenetic analysis between *Nowenia* and *Oricilla*, *Tarella*, *Zosterophyllum* and *Forania* (Fig. 15) require closer scrutiny.

*Oricilla* (Gensel, 1982) and *Tarella* (Edwards and Kenrick, 1986) share many characters with *Nowenia* but also possess several characters that clearly differentiate them from *Nowenia* (Table 2, Supplementary Data Note). Interestingly, *Oricilla* is similar to *Nowenia* in the patterning of epidermal cells, which form rosettes (Gensel, 1982), but this feature is also shared by *Sawdonia* (Edwards *et al.*, 1982). The genus *Zosterophyllum* includes many species, all of which differ from *Nowenia* in having sporangia grouped into fertile zones (Table 2), in one or more rows (e.g. Edwards, 1969b; Gensel, 1982) and some forming strobili (Edwards, 1969*a*, 1975).

**Table 2.** Morphological comparisons of *Nowenia* and closely related taxa (as recovered in the phylogeny of Claisse *et al.*, 2025).

	*Nowenia*	*Forania*	*Zosterophyllum*	*Oricilla*	*Tarella*
Branching angle	Branches parallel to subtending axis	Branches parallel to subtending axis	Branches parallel to subtending axis	Branches parallel to subtending axis	Branches parallel to subtending axis?
Subaxillary branching	Absent	Absent	Absent	Absent	Absent
Circinate axis tips	Present	Present	Absent	Present	Present
Sporangium grouping	Absent	Absent	Strobili	Present	Present
Sporangium orientation	± Apically	± Apically	± Apically	Lateral (outwards)	Lateral (outwards)
Sporangium shape	± Reniform	± Reniform	± Reniform	± Reniform	± Reniform
Sporangium dorsiventral flattening	Present	Present	Present	Present	Present
Sporangium valves relative size	Isovalvate	Isovalvate	Isovalvate	Isovalvate	Isovalvate
Sporangium dehiscence line thickened	Absent	Present	Absent	Present	Absent
Sporangium stalk size	Short	Short	Short	Short	Short
Sporangium stalk length/width	<1	?	Varies with species	<1	<1
Reference	This study	Jensen and Gensel (2013)	Edwards (1975)	Gensel (1982)	Edwards and Kenrick (1986)

*Forania* (Jensen and Gensel, 2013) does not possess grouped sporangia and is placed as sister to *Nowenia* in the phylogeny (Fig. 15). *Forania* shares many features with *Nowenia* (Table 2), including dormant branch meristems and U-patterned branching and absence of subaxillary tubercles; like *Nowenia*, *Forania* may have had K-branching, and its architecture is interpreted to include both decumbent and upright axes. The sporangial dehiscence line is described as thickened in *Forania* (and scored as such in the phylogenetic matrix). The line of dehiscence of *Nowenia* is very similar to that illustrated in *Forania* cuticular material. However, based on our examination of both the cuticular material of *Nowenia* sporangia (Fig. 11A, B) and the sporangia exposed on bedding planes (Fig. 10D, F), we exclude the possibility of a thickened dehiscence line being present. A more significant difference is that unlike *Nowenia*, *Forania* has anisotomous branching. Additionally, in *Forania* the undeveloped dormant branches are coiled abaxially rather than adaxially and *Forania* axes bear two rows of large multicellular spinescent projections with putative secretory function (Jensen and Gensel, 2013). These differences, and especially the presence in *Forania* of the spinescent projections that are diagnostic at the generic level in zosterophylls (Jensen and Gensel, 2013), exclude *Forania* from consideration as a possible taxonomic placement of the new zosterophyll.

**Fig. 16.**
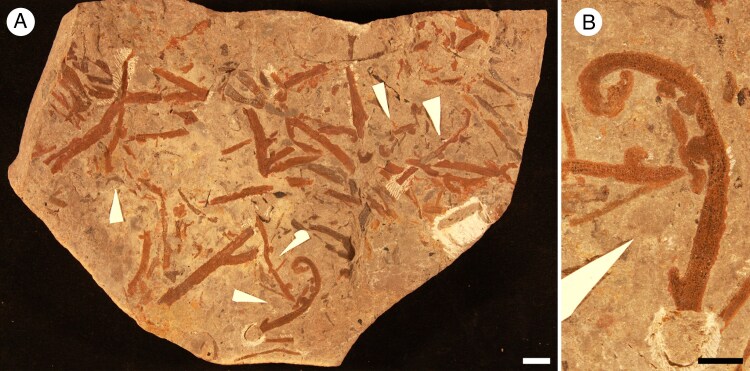
*Gosslingia americana* Tanner (1982) (white arrowheads were placed on the specimen by previous researchers). (A) Rock slab containing all known specimens of *G. americana* (arrowheads by Tanner). FMNH PP15950. Scale bar = 1 cm. (B) Detail of (A). Holotype of *G. americana* showing sporangia within circinate coiled apex, similar to *Nowenia matsunagae*. Scale bar = 5 mm.

*The conundrum of* Gosslingia americana *Tanner. Gosslingia americana* is a species described by Tanner (1982) in the Cottonwood Canyon assemblage from six oxidized, fragmentary fertile specimens of small size and a few associated sterile axis fragments (Fig. 16). This zosterophyll comes from the same locality as *Nowenia*, with which it shares some features. One of the features is the circinate apices that bear sporangia; the holotype of *G. americana* is one such circinate axis portion with four laterally attached sporangia, of which one is in the still-coiled tip (Fig. 16B; compare with Fig. 10E). Another feature may be the U-patterned branching morphology, which is present in a few sterile axis fragments preserved in the vicinity of the fertile *G. americana* fragments, on the same hand specimen (Fig. 16). These shared features raise the question of whether *G. americana* and *Nowenia* may represent the same taxon, in which case the former name would take chronological precedence. Three considerations discussed below are relevant to addressing this question. They concern (1) whether the specimens assigned to *G. americana* belong in the genus *Gosslingia*; (2) whether there are enough points of similarity between the *G. americana* specimens and the material assigned to *Nowenia*; and (3) whether the *G. americana* specimens could be conspecific with any other taxon present in the same fossil assemblage.

First, the assignment of Tanner’s specimens to the genus *Gosslingia* is questionable, both because the combination of characters Tanner used to justify this placement (circinate tips, smooth axes and laterally positioned elliptical sporangia) are present in several other zosterophyll genera, and because subaxillary tubercles, a feature diagnostic of the genus *Gosslingia* (Edwards, 1970), cannot be demonstrated in Tanner’s material. Second, the points of similarity between *Nowenia* and the specimens assigned to *G. americana* are few and equivocal. The two cannot be compared in terms of branching pattern because none of the *G. americana* specimens are branched. The similarity in the coiled axis apices is inconclusive, as this feature is present in several other zosterophylls. Furthermore, the *Nowenia* material, despite comprising several hundred specimens, does not include any specimens comparable to *G. americana* in the high number of sporangia present in an apical crozier. In fact, the distribution of sporangia on the fertile specimens of *Nowenia* suggests that they were not produced in closely spaced series along the axes. Additionally, at 2.20 × 3.50 mm maximum size, the sporangia of *G. americana* are significantly smaller than those of *Nowenia* (3.70 × 4.15 mm maximum size). Third, our investigation of the Cottonwood Canyon material has produced evidence for the presence of two other previously unrecognized zosterophyll morphotypes (see second paragraph below). This broadens the range of taxa with which the *G. americana* specimens could be conspecific. Because these two types are known only from vegetative specimens, their conspecificity with *G. americana* cannot be tested but it also cannot be rejected a priori.

Together, the above considerations imply that (1) the assignment of *G. americana* to the genus *Gosslingia* is debatable (as pointed out by Tanner himself; Tanner, 1982); (2) similarity between *Nowenia* and the specimens assigned to *Gosslingia* is superficial and there are, instead, several differences between the two; and (3) the range of taxa with which the specimens assigned to *G. americana* could be conspecific now includes two other zosterophylls, but such conspecificity cannot be tested due to insufficient data. Consequently, conspecificity between the specimens assigned by Tanner to *G. americana* and the material we assign to *Nowenia matsunagae* is rejected, warranting erection of the latter new taxon. For now, Tanner’s (1982) specimens are best considered zosterophylls *incertae sedis* and not assigned to any genus.

*Other zosterophylls present in the Cottonwood Canyon assemblage.* Aside from *Gosslingia americana*, two other Cottonwood Canyon fossil morphotypes assignable to the zosterophyll group share with *Nowenia* the same distinctive branching pattern, where branches run more or less parallel to the subtending axes. Comparative evidence allows us to distinguish these morphotypes from *Nowenia* and from each other. One of the morphotypes (Zosterophyll 1) has overall thicker axes (Supplementary Data Fig. S3A–C) that bear dense, fine spine-like projections. A specimen that bears this type of projection and exhibits K-branching (of a peculiar type, forming an X-shaped pattern; Supplementary Data Fig. S3C), known only among zosterophylls and lycopsids, supports zosterophyll affinities for this plant. The second morphotype (Zosterophyll 2) exhibits the same fine sinuous longitudinal creases along the axes as *Nowenia*, with dormant branch meristems (Supplementary Data Figs S3D and S2E), but in contrast to the latter it has subaxillary tubercles (Supplementary Data Figs S3D and S2E), which also supports its zosterophyll affinities. Also distinguishing Zosterophyll 2 from *Nowenia* are its unequal branching and its much lower taper coefficient (∼0).

## DISCUSSION

### Epidermis, sporangia and spores

*Cuticular anatomy comparisons (see Supplementary Data Note for extended version).* Three types of epidermal features documented in cuticular material of *Nowenia* merit discussion: the surface sculpturing of epidermal cells consisting of a central papilla and radiating ridges; the groups of cells forming rosette patterns; and the stomata.

The central papilla and radiating ridges seen in some of the epidermal cells of *Nowenia* are strongly similar to those of *Sawdonia ornata* and best interpreted, like the latter, as features of the living plant (Edwards *et al.*, 1982; Rayner, 1983). An alternative interpretation, as taphonomic features – specifically, resulting from shrivelling of the epidermal cells that originally had convex outer periclinal walls (Rayner, 1983) – requires testing by accurate measurement of the radiating ridges, which is beyond the scope of this work. If they are indeed features of the living plants, the papilla and radiating ridges could have had functions in light direction within the underlying cell (Rayner, 1983), in herbivore deterrence (e.g. Surapaneni *et al.*, 2020), or in water capture (by enhancing condensation on the surface of the epidermis) and capillary water retention; the water-related functions could have also promoted plant irrigation, by concentrating condensation water in capillary films around the papillae, until it dripped under the plant.

The rosette patterns formed by groups of cells in the epidermis of *Nowenia* have been documented in at least five or six other zosterophylls. They have been variously interpreted as the bases of fine trichomes based either on direct evidence (the trichome bases of *Trichopherophyton*; Lyon and Edwards, 1991 ) or on similarity with such structures from extant plants (Edwards *et al.*, 1982; Gensel, 1992); as structures with secretory or storage functions (Hueber and Banks, 1979); or as structures concerned with water relations (Edwards *et al.*, 1982; Gensel *et al.*, 2025). If the epidermal cell rosettes of *Nowenia* mark the location of trichome bases, the trichomes have yet to be found, although if they were as fine as those of *Trichopherophyton* (which is likely considering the similar cell sizes), they may not have withstood the taphonomic conditions that affected the *Nowenia* fossils.

The preservation of the cuticle in *Nowenia* conceals the cellular patterns around the stomatal guard cells. Considering that zosterophyll stomata are typically anomocytic (Guo and Wang, 2016), the stomata of *Nowenia* may also be anomocytic. Their guard cells ∼95 µm long surpass slightly and extend the range of previously measured Early Devonian guard cells, which ranged from 22 to 86 µm (Lomax *et al.*, 2014), with the longest guard cells previously documented in *Horneophyton lignieri* (Edwards *et al.*, 1998).

*Scarcity of sporangia.* Sporangia are rare in the *Nowenia* material; only 21 of the >600 specimens bear sporangia and of these only one specimen preserves two sporangia (Fig. 10B) and another one three sporangia (Fig. 10C). The paucity of sporangia in *Nowenia* fits the general pattern observed in the Cottonwood Canyon assemblages: a survey of 6939 Cottonwood Canyon plant fossils recorded only 50 specimens that bore sporangia (A. C. Bippus, Save the Redwoods League, San Francisco, USA, pers. comm.). Among these, in the lycophyte *Sengelia*Matsunaga and Tomescu (2017) reported sporangia in only 20 stems out of more than 400 surveyed; they hypothesized that this was due to a growth dynamic that emphasized clonal propagation in a floodplain environment where frequent floods buried the *Sengelia* populations, reducing significantly the effectiveness of propagation by spores. The taphonomy of the *Nowenia* material (see Taphonomy below) indicates that populations of this plant did not occupy the same environments as *Sengelia*. Nevertheless, *Nowenia* plants maintained dormant branch meristems, which are present with higher abundances than the sporangia (42 specimens are preserved with one or more dormant branch meristems) and are believed to indicate unstable growth environments (Edwards and Kenrick, 1986; Gensel, 1992), in which they allowed resprouting and renewed propagation following adverse events. Thus, the scarcity of sporangia in *Nowenia* may still be attributable to instability in the growth environment. Additionally, the samples consisting of monotypic mats of *Nowenia* axes suggest that this plant formed monodominant stands, a growth mode thought to be favoured especially in unstable environments by a life history that emphasizes rapid vegetative growth with limited sporulation events (Hotton *et al.*, 2001). Monodominant stands and limited interspecific interactions are believed to have been common in Early Devonian plant communities (Gensel and Andrews, 1984; DiMichele and Hook, 1992) and the overall body plan of *Nowenia* supports this interpretation, as the decumbent parts of the plant are consistent with rapid propagative growth.

*Spores.* A palynological study of the Cottonwood Canyon layers that yielded the *Nowenia* material (Noetinger et al., 2021) has documented 37 types of dispersed palynomorphs. Of these, *Ambitisporites*, *Aneurospora* and *Retusotriletes* are similar to the spores of *Nowenia* in their lack of surface sculpturing. Among these, only *Retusotriletes* includes species thought to have been produced by zosterophylls. Of the four *Retusotriletes* species recognized at Cottonwood Canyon, only *R. actinomorphus* is thought to have been produced by zosterophylls (Noetinger *et al.*, 2021), whereas the other three are either associated with trimerophytes or have unknown macrofossil affiliations. While the *in situ* spores of *Nowenia* are similar in size to those of *R. actinomorphus*, because they are also comparable to other spore types from the palynomorph assemblage and because *Nowenia* is not the only zosterophyll at Cottonwood Canyon, they cannot be unequivocally assigned to *R. actinomorphus*.

### Taphonomy, growth environment and interactions of *Nowenia*

The fossiliferous strata of the Beartooth Butte Formation at Cottonwood Canyon were deposited in river floodplain areas with an oscillating water table that experienced periodic floods (Matsunaga and Tomescu, 2017; Noetinger *et al.*, 2021). Represented by several hundred specimens, *Nowenia* is one of the most abundant plants in the Cottonwood Canyon assemblages, higher in abundance by two orders of magnitude compared with the next best-represented plant type. However, its abundance is one order of magnitude lower than that of the most abundant species, *Sengelia radicans*, which forms autochthonous fossil associations or is preserved *in situ* in the floodplain sediments (Matsunaga and Tomescu, 2017). Importantly, the *Nowenia* material is preserved in beds interpreted as flood deposits and many of the specimens show relatively high fragmentation. Together, these observations suggest that rather than growing in the floodplain, *Nowenia* populations occupied areas adjacent to the floodplain, and thus their fossils are parautochthonous, having undergone some transport over relatively short distances. This interpretation is supported by the relatively large size of some of the *Nowenia* specimens (low degree of fragmentation) and the occasional occurrence of *Nowenia* axes in monotypic associations on bedding planes.

Also consistent with the parautochthonous interpretation of the *Nowenia* fossils is the presence of microconchids (encrusting aquatic lophophorates) on some of the *Nowenia* axes. The frequency and distribution of these encrusting invertebrates on stems of the lycopsid *Sengelia* have been shown to indicate that they colonized populations of this plant while they were submerged during periodic floods (Dorn *et al.*, 2017; Matsunaga and Tomescu, 2017). If the presence of microconchids on *Nowenia* axes has the same causes (colonization while the plants were alive), the low frequency of microconchids would indicate that *Nowenia* populations did not grow close enough to the floodplain environment to become submerged at the same frequency or with the same durations as *Sengelia*. However, we cannot exclude that the microconchids may have colonized *Nowenia* axes *post mortem*, during transport and prior to burial.

The *Nowenia* axes forming dense mats, in which axes have uneven thickness and bear more marked longitudinal folds, could indicate drying-related shrinking. If so, it is hard to ascertain whether the axes experienced the shrinkage prior to uprooting and transport or after transport and prior to burial. In the first scenario, transport of drying–shrunken axes would have had to be brief, otherwise the axes would have had time to rehydrate prior to deposition and burial. This interpretation is supported by the fact that the axes form mats, which would have become disentangled and fragmented over longer transport distances and times. Under this scenario, the co-occurrence on the same bedding planes and the close vicinity of shrivelled and non-shrivelled specimens would reject the alternative scenario – shrinkage after transport and prior to burial –, under which all specimens preserved in the same spot would have been subjected to drying and shrinkage. However, none of the samples containing mats of shrivelled axes are large enough to allow unequivocal rejection of this alternative scenario.

The marked darkening (and possibly thickening) of the cuticular material associated with the surface features of *Nowenia* interpreted as traces of herbivory is consistent with responses of living tissues and would, thus, indicate that they are *in vivo* lesions.

### Place of *Nowenia* in zosterophyll diversity and the Early Devonian fossil record

The Beartooth Butte Formation hosts the only diverse Early Devonian flora known in western North America. However, its diversity has been surveyed thoroughly only once, more than 40 years ago (Tanner, 1983), and the results of that survey have not been published formally. The publications that preceded Tanner’s survey were focused on the other locality of this unit, Beartooth Butte, which may have a different age and represent a different depositional environment (Elliott and Ilyes, 1996; Elliott and Johnson, 1997; Fiorillo, 2000). Only two taxa are documented in significant detail in these prior publications (Hueber, 1972; Steenbock and Tomescu, 2013), with a few other taxa based only on small numbers of fragmentary or sterile specimens, some with questionable taxonomic assignments (Dorf, 1933, 1934). Since the discovery of the Cottonwood Canyon locality (Blackstone and McGrew, 1954; Sandberg, 1961) and Tanner’s (1983) survey, continued collecting by several teams has added large numbers of specimens and diversity to the flora of this locality. However, to date only one plant species has been characterized in detail from these rich collections – *Sengelia radicans* (Matsunaga and Tomescu, 2017). *Nowenia* is thus the second species described in detail from the Cottonwood Canyon locality and one of the very few thoroughly characterized species from the Beartooth Butte Formation. Moreover, *Nowenia* is, alongside *Sengelia*, one of the few Early Devonian species for which empirically based whole-plant concepts constructed using extensive morphometric data exist, and the only zosterophyll in this category.

*Nowenia* is one of three distinct zosterophyll morphotypes currently identified in the Beartooth Butte Formation, which, given its age and geographic location, marks a key data point in Early Devonian phytogeography. In the context of the overall Early Devonian plant diversity, *Nowenia* adds a new member to the 37 or more currently recognized zosterophyll genera, a group that accounted for the bulk of plant diversity and biomass in early tracheophyte floras, particularly in the first half of the Early Devonian. The relatively subtle yet significant characters that differentiate *Nowenia* from similar zosterophyll genera (1) demonstrate that even the simple body plans of early tracheophytes allow room for much morphological disparity; (2) throw additional light on the levels of morphological detail that are relevant from a taxonomic standpoint; and (3) demonstrate the importance of empirically based, accurate whole-plant concepts for documenting past plant diversity. Future studies including all three zosterophylls identified in the Cottonwood Canyon flora (when fully characterized) will update the picture of zosterophyll biodiversity and biostratigraphy.

### A morphometrics-informed whole-plant concept

*The three morphologies represent the same species. Nowenia matsunagae* is represented in the fossil assemblage by fragmentary specimens with three different morphologies. The ideal type of evidence for demonstrating conspecificity of fragmentary plant fossils with different morphologies or representing different organs is provided by instances of physical connection between the different morphologies (e.g. the archaeopterid progymnosperm concept – *Archaeopteris* and *Callixylon* – of Beck, 1960). In the absence of physical connection, conspecificity has been demonstrated based on shared features with characteristic morphology (e.g. the capitate glands of lyginopterid pteridosperms; Oliver and Scott, 1905). This second type of evidence can be bolstered by consistent co-occurrence of the different morphologies or plant parts (Stewart and Rothwell, 1993).

The three morphologies of *Nowenia* have not been found in physical connection, thus far. However, the decumbent and tufted morphologies are recognized as conspecific based on a set of shared features: naked axes devoid of any protrusions, with more or less sinuous habit; branches of the same thickness as their subtending axes that run parallel to the subtending axes; absence of subaxillary tubercles; vascular strand, if visible, relatively thin compared with the width of the axis. Additionally, axes of the two morphologies often occur in monotypic or monodominant associations on bedding planes and the two morphologies are not entirely distinct but, rather, they intergrade. Axes with these two morphologies can be distinguished from superficially similar specimens that represent other taxa, based on discrete features and morphometric comparisons (see Systematics and Remarks sections, above).

Given the several hundred specimens of *Nowenia* examined in this study, the fact that only one has been documented unequivocally with K-branching may raise doubts about its attribution to *Nowenia*. This attribution is supported by the lack of any protrusions on the axes of the K-branched specimen and the presence of the fine sinuous longitudinal creases that characterize *Nowenia* axes. Additionally, this specimen has yielded cuticle that, although preserved suboptimally, presents cellular patterns of size and morphology closely similar to those of the other *Nowenia* axes, including cells grouped in a rosette pattern. Furthermore, only two other plants are known to possess K-branching in the Beartooth Butte Formation and the specimen assigned here to *Nowenia* is different from either of these two. One of the two is *Sengelia radicans* (Matsunaga and Tomescu, 2017), which has K-branching in the leafy stems. The other one is a new plant morphotype referred to here as Zosterophyll 1 (see Systematics and Remarks sections, above), which bears spine-like projections and exhibits a peculiar type of K-branching. In this context, it is also important to note that in *Nowenia* dormant branch meristems are often present on lateral branches close to their bases (at 10 mm from the base, on average; Figs 6C and 9F, G); such dormant branch meristems could represent K-branching points with one incompletely developed branch.

*Morphometric evidence for growth patterns and growth habit of* Nowenia. The increase in CBS between immature (dormant branch meristems, branch croziers) and well-developed (mature) branches (Fig. 6F) implies that young branches underwent some thickening (at the base) as they grew in length. The subunitary values of the CBS for immature branches are consistent with branching by unequal dichotomies of the apical meristem. Such branching typically results in anisotomous branching systems that can range all the way to extreme anisotomy (i.e. pseudomonopodial branching). Indeed, *Nowenia* axes of decumbent morphology appear pseudomonopodial (e.g. Figs 4L–O and 5L–O). However, their mature branches have CBS ≈1, which is typical of isotomous branching patterns produced by equal apical dichotomies. *Nowenia* axes of tufted morphology that approach isotomous branching patterns (e.g. Figs 4B, D, F, H and 5B, D, F, H) and, predictably, have CBS ≈1 for their mature branches, also possess immature branches with CBS <1, suggestive of origin from unequal apical dichotomies. Thus, the morphology of *Nowenia* provides an interesting example of how a developmental programme that combines unequal apical dichotomy followed by increase in branch thickness can produce, paradoxically, two branching architectures, depending on the amount of internode elongation: isotomous architectures derived from unequal apical dichotomies (in the tufted morphologies); and pseudomonopodial architectures in which branches are equal in thickness to the subtending axes (in the decumbent morphologies).

Specimens that are too fragmentary to allow measurement of internode lengths or calculation of tuft coefficients – comprising the majority of *Nowenia* specimens – cannot be assigned directly to one of the two main morphologies that make up *Nowenia* plants. However, the distribution of taper coefficients (Fig. 7D, E) suggests that, for *Nowenia*, this metric may provide a proxy assisting with morphotype assignment. This is because the taper coefficients calculated for all available specimens fall within two disjunct ranges and, among these, specimens that can be assigned unequivocally to the two morphologies are separated between the two ranges of taper coefficients (Fig. 7E). Taper coefficients also suggest different growth patterns between the two morphotypes. The higher taper coefficients of the tufted morphotype suggest that they may have had somewhat limited growth, probably by decrease in apical meristem size associated with successive apical dichotomies, in contrast to the decumbent morphotype, which has virtually no taper.

The frequency distribution of internode lengths indicates that shorter internodes are more numerous than long and intermediate size internodes (Fig. 6B). Indeed, when comparing the ranges of internode lengths between the degree of development of the stages of branch development they separate (Fig. 6C, D), all three categories have comparable minima. This is due, in part, to the fact that specimens with decumbent morphologies have some shorter internodes that are comparable to the typically shorter internodes of the specimens with tufted morphologies (Fig. 6D). Independently of this, the internodes separating fully developed branches are longer than internodes adjacent to less developed branches, on average and in terms of the range maxima (Fig. 6C). These data have two implications for *Nowenia* development. One is that axis segments immediately below a branching point continued to elongate for a little while after the branching event. Because the developmental stage of a branch is a proxy for the age of the internode that subtends it, if internodes were not elongating after the branching event, we would see the same length ranges for internodes separating branches in all developmental stages. If axes underwent some elongation below branching points, this implies that rather than growing only by cell elongation immediately behind apical meristems, *Nowenia* axes were also growing to some extent by diffuse cell growth (elongation) throughout the internodes. The second developmental implication of fully developed branches being separated by longer internodes than developing branches is that branch meristems did not experience extended dormancy and probably started growing out only a few nodes below the growing axis tips (otherwise we would see branches in different developmental stages separated by internodes of similar lengths).

The correlation between the orientation of the sporangia and the size of their subtending axes (Fig. 7F) indicates that sporangium orientation changed during development, a feature described here for the first time in a zosterophyll. Sporangia started out with their mediolateral plane parallel to the subtending axis, as seen in the youngest sporangia found on circinate axis tips (Fig. 10E). As the sporangia matured and the subtending axes continued to elongated and gained in thickness, the sporangia became oriented obliquely with respect to the axes, until the sporangia found on the thickest axes were oriented with their mediolateral plane perpendicular to the subtending axis (Fig. 10F).

Our interpretation of the sparser-branched morphotype as representing decumbent portions of the plant is based on three types of consideration. First, the slender nature of the axes, compared with their extent, suggests a non-self-supporting habit: the longest specimens, one of them >19 cm long, show very little taper, which indicates that they were much longer than that, with thicknesses of no more than 6.5 mm. Second, the axes show no evidence of any significant presence of mechanical support tissues, which would be required in significant amounts to hold upright such slender axes: none of the specimens consist of excessively thick coalified material and some (e.g. Figs 2C, D and 5J) clearly show around the central vascular strand lighter-coloured material indicative of thinner-walled or larger-celled tissue forming the cortex. Third, studies of the biomechanics of stele shape and of the types and proportions of tissues surrounding the stele have indicated that the vast majority of early tracheophyte axes were turgor systems, i.e. turgidity of parenchymatous tissues contributed an overwhelming proportion (>90 %) of their flexural stiffness (Speck and Vogellehner, 1988), so they could not have maintained an upright posture under even small reductions in turgor pressure; axes with the dimensions and inferred histology of those of the sparsely branched *Nowenia* axes are unlikely to have been able to maintain an upright posture under such mechanical constraints, given even modest changes in water availability. Although we have not tested the mechanics of *Nowenia* axes, so that these considerations may sound speculative when applied to it, together they make a reasonable case for our interpretation of decumbent axes. An argument could be made that, given zosterophyll axes of similar properties, previous authors have reconstructed them as upright portions of plants. However, we are not aware of any such previous reconstruction in which the interpretation of slender axes with upright posture was backed with arguments based on their morphology. Another argument that could be made is that the U-patterned branching with the branches and subtending axis growing in the same direction would not make sense for decumbent, rhizomatous axes that could be expected to branch and grow more variably, in all directions, as opposed to unidirectionally, for most effective ‘foraging’ for resources; and that the U-patterned branching makes more sense for branching systems growing upright and exhibiting geotropic or phototropic responses in all their segments. However, this argument is weakened by the several zosterophyll taxa that exhibit consistently U-patterned branching, which suggests that this branching type reflects a well-entrenched (generalized) developmental programme in the group (or at least in a subset of taxa), rather than being associated with a specific posture. Additionally, it is not unusual for the branching rhizomatous portions of the same plant, such as the extant *Lycopodium clavatum*, to grow in the same general direction or even parallel to each other. For all these reasons, we think that although alternative interpretations cannot be ruled out definitively, our interpretation of the sparsely branched axes of *Nowenia* as representing decumbent portions of the plant is the best supported.

The decumbent portions of *Nowenia*, thus, represent parts of the plant that allocated resources primarily to elongation, probably in a creeping habit, to explore the environment for resources or, possibly, to avoid competition. The decumbent axes would have been produced by K-branching of rhizomatous portions, which may have grown partially below ground, and been consequently less prone to enter taphonomic processes, consistent with their rare occurrence in the allochthonous fossil assemblage. The decumbent axes possibly bore rhizoid-type appendages that were not preserved (we were unable to identify any). This would not be surprising, given that despite an abundance of Early Devonian plant fossils, many of which had rhizomatous axes, the only such fossils that preserve unequivocal rhizoids are the plants preserved exquisitely by permineralization in the Rhynie chert (e.g. Lyon and Edwards, 1991; Edwards, 2003).

Conversely, tufted specimens would correspond to parts of the plant that allocated resources primarily to branching and production of sporangia, and were probably growing upright. Indeed, with two exceptions, sporangia were observed only on tufted specimens; the two exceptions – sporangia attached to decumbent specimens – may represent instances of sporangium production on young, upright bending apical regions that became subsequently decumbent following further elongation along the respective axes. The fact that specimens of tufted morphology, thought to have grown upright, are on average smaller in overall size that those with decumbent morphologies (Figs 4, 5 and 7E) probably reflects (1) a taphonomic difference (upright plant parts are more likely to enter taphonomic pathways that lead to their fragmentation); and (2) different developmental constraints (given the thickness of their axes, the tufted parts could not grow too tall and remain self-supporting). These and the other implications of the morphometric data discussed above form the basis for reconciling and integrating the different morphologies into a whole-plant concept for *Nowenia*.

**Fig. 17.**
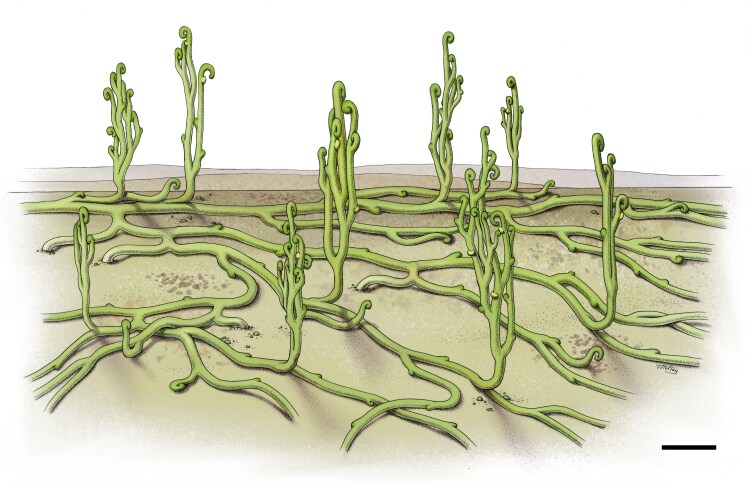
*Nowenia matsunagae* gen. et sp. nov. Whole-plant concept; reconstruction rendered by Jamie Hefley (https://mbiportfolio.com/JamieHefley/). This whole-plant concept includes all the metrics developed using the data collected from the numerous specimens analysed: the proportional contribution of the two branching morphologies (decumbent and upright) to the architecture of sporophytes, for a given cumulative length of axes considered; the ranges of thickness, taper and branching densities of axes in the two morphotypes; the proportion of branches in different developmental stages (dormant branch buds, undeveloped circinate branches, fully developed branches); the thickness ratios between subtending axes and branches; and the proportional number of sporangia present for a given cumulative length of axes considered. The growth habit of *N. matsunagae* favoured propagation by horizontal vegetative growth. The sporophytes consisted primarily of decumbent axes with rhizomatous growth that branched apically, sometimes producing K-branches, and bore branching systems with upright growth. Branches, produced roughly in an alternate pattern, were similar in thickness to the subtending axes. They diverged at acute angles, curving in an apical direction and running parallel to the main axes. Some branches had delayed development, appearing as lateral branch buds or circinate lateral protrusions. Small, isolated reniform sporangia (shown in yellow) were borne primarily on the upright portions in relatively small numbers. Scale bar = 3 cm.

*Building the* Nowenia *whole-plant concept.* The whole-plant concept we present here (Fig. 17) is based on, and true to, all the metrics developed using the data collected from the numerous specimens analysed. It takes into account the proportional contribution of the two branching morphologies (decumbent and upright tufted) to the architecture of sporophytes, for a given cumulative length of axes considered; the ranges of thickness, taper and branching densities of axes in the two morphotypes; the proportion of branches in different developmental stages (branch primordia, undeveloped circinate branches, fully developed branches); the relative thickness of branches and subtending axes; and the proportional number of sporangia present for a given cumulative length of axes considered.

The steps we followed in building the whole-plant reconstruction are as follows.

(1) We started out by sketching a rhizomatous branching system based on the morphology (slightly sinuous axes with very low taper and U-patterned branching) and metrics of the decumbent morphotype: range and mean of axis thickness; percentage of branch meristems vs developed branches; sizes of branch meristems and developed branches with respect to sizes of their subtending axes; and ranges and means of distances between branches at different developmental stages along the main decumbent axes.

(2) The frequency of K-branching points with respect to the total length of decumbent axes depicted is speculative, higher than their proportion in our material; their axis sizes and branching metrics are based on the specimen in our material, which is also an instance of reiterative K-branching, as depicted at one place in the reconstruction. Because they typically represent points of attachment to the substrate, K-branches would only be found with frequencies approaching their real frequency of occurrence in fossil assemblages preserved *in situ*, but they are less likely than other parts of the plants to enter taphonomic processes and become fossilized in transported assemblages; thus, they are likely under-represented in our material, hence our decision to show them in higher numbers in the reconstruction.

(3) The branching points that did not involve branch meristems of K-branching were split between three categories: those producing other decumbent axes, those bearing developing decumbent branches with circinate tips, and those bearing tufted branching systems.

(4) The number of tufted branching systems depicted as originating from the decumbent system of axes was chosen to reflect roughly the ratio between the total cumulative lengths of decumbent vs tufted specimens, as calculated for specimens that we could place with certainty in each of the two categories.

(5) The remaining branching points of the decumbent system (i.e. those not bearing branch meristems, tufted branching systems or K-branching) were split arbitrarily between developing decumbent branches with circinate tips and fully developed decumbent branch axes.

(6) We used the three most extensive tufted branching systems in our material and some combinations thereof, with little modification of morphology and reflecting their measured range of axis thickness, as models for the tufted parts of the reconstruction; the circinate tips of their branches, not preserved in our material, were added based on specimens in our material that demonstrate that *Nowenia* axes had circinate tips. Growing axis tips are among the most delicate parts of the plants and, as such, the least likely to withstand taphonomic processes and become fossilized in transported assemblages like the fossil assemblage that yielded the *Nowenia* material.

(7) All the circinate tips depicted in the reconstruction reflect the size range of such tips measured in our material.

(8) The sizes of sporangia reflect the sporangium metrics in our dataset; the distribution of sporangia along axes reflects their distribution (isolated) in the material; the number of sporangia shown in the reconstruction matches roughly their frequency with respect to the cumulative axis length observed; the orientation of sporangia on axes reflects our direct observations, with those located close to or in circinate tips oriented with the mediolateral plane parallel to the subtending axis, whereas those located farther from growing tips are oriented with their mediolateral plane perpendicular to the subtending axis.

(9) The completed final sketch was traced and coloured by the artist Jamie Hefley.

The resulting whole-plant concept shows *Nowenia matsunagae* plants with an overall growth habit that favoured propagation by horizontal vegetative growth (Fig. 17). A great proportion of the sporophyte (∼70 %) consisted of decumbent axes with rhizomatous growth that branched apically, sometimes producing K-branches, and bore branching systems with upright growth. The axes of *Nowenia*, typically 3–4 mm thick, could reach 6.5 mm in thickness and had circinate apices. Branches, produced roughly in an alternate pattern, were similar in thickness to the subtending axes. They diverged at acute angles, curving in the apical direction to become parallel to the main axes. Some branches had delayed development, appearing as lateral branch primordia and subsequently as circinate lateral protrusions that unfurled as they developed. The decumbent portions branched at 3-cm intervals, on average, while the upright portions had a bushy aspect, with denser branching (every 1.7 cm, on average) and with limited growth. *Nowenia* plants would have been similar in branching architecture to a number of lycopsid species, such as *Lycopodium clavatum* and *Selaginella willdenowii*. In these plants, a main rhizomatous stem with indeterminate growth and sparser branching produces densely branched lateral branching systems with limited growth. In *Nowenia*, small isolated reniform sporangia were borne in relatively small numbers, primarily on the upright tufted portions. The sporangia, conspicuous in early developmental stages in the circinate apices of the axes, were initially oriented with their mediolateral axis parallel to the subtending axes; as they matured, they became oriented with their mediolateral axis perpendicular to the subtending axes.

### Reconstructing whole-plant concepts using large morphometric datasets

Among the plethora of whole-plant reconstructions produced by palaeobotany since the 19th century, those that are supported empirically by comprehensive morphometric datasets obtained from large numbers of specimens are rare. One of these is Blanco-Moreno and Buscalioni’s (2023) reconstruction of the Cretaceous fern *Coniopteris laciniata*. Based on morphometric data from 66 specimens, these authors demonstrated a continuum of pinnule variation between two extreme morphologies previously assigned to two different genera, supporting their conspecificity. This further solidified interpretations of homology between frond segments, reconstruction of the growth habit and architecture of the plant, understanding of the growth environments, and developmental and morphological adaptations. Although different in scope, the study of frond architecture of the Cretaceous fern *Weichselia reticulata* by Blanco-Moreno *et al.* (2019) also illustrates the power of morphometric studies of large fossil samples. In their study, the authors devised a protocol for characterizing frond architecture using quantitative data and used it to compare frond architecture in different genera and to map the location of isolated frond fragments on the architecture of complex fronds.

For the Early Devonian, *Hsüa deflexa* was reconstructed by Wang *et al.* (2003) based on observations of more than 200 specimens and measurements of subsets of these specimens. The whole-plant reconstruction revealed a rhizomatous growth habit bearing slender upright fertile branches. Considered in the context of the depositional environments of the fossiliferous layers, the reconstruction led to a hypothesis correlating the growth habit of the plant with its growth environment. In another example, working on the Cottonwood Canyon assemblage that yielded *Nowenia*, Matsunaga and Tomescu (2017) produced a whole-plant concept for the early lycopsid *Sengelia radicans*. Their *Sengelia* reconstruction reflects accurately the range of morphological variables recorded in the >600 specimens examined and represents the best supported whole-plant concept of a drepanophycalean lycopsid, which allowed refining the taxonomy of several members of the group. Combined with observations of sedimentology and plant taphonomy, the whole-plant reconstruction led to an in-depth understanding of the growth environments and growth dynamics of *Sengelia*.

Among zosterophylls, in fortuitous cases such as that of *Zosterophyllum shengfengense* (Hao *et al.*, 2010), preservation of whole plants leaves no doubt about any aspect of the gross morphology of the species. However, among the other published zosterophyll reconstructions few make extensive use of quantitative data. For those that do (e.g. Edwards, 1970; Gerrienne, 1988; Berry and Gensel, 2019), the numbers of specimens used in data collection or the approaches taken to integrating the quantitative data into the reconstructions are rarely documented, so it is hard to know exactly how the whole-plant concepts took shape.

In the context of existing whole-plant concepts, *Nowenia*’s is the only zosterophyll reconstruction that makes explicit and extensive use of quantitative data, and one of the rare such reconstructions of fossil plants, in general. Along with the whole-plant concept of *Sengelia radicans* (Matsunaga and Tomescu, 2017), these are currently the best empirically supported reconstructions of Devonian plants, from a quantitative standpoint. Their insightful implications for different aspects of the life histories of these plants – growth architectures, living environments, propagation dynamics – demonstrate the value of whole-plant concepts informed directly by large morphometric data sets.

### The importance of reliable and detailed whole-plant concepts in evolutionary biology

Fossils are the only direct evidence for the deep history of life and, as such, they provide the only independent way to test hypotheses on biological evolution constructed based on the study of living life forms. To effectively integrate fossils in these approaches, one of the twin overarching goals of palaeobotany is to discover and understand extinct plant species as biological entities in their environments, on a par with their extant counterparts (the other goal being to document the past history of living species). Understanding extinct species as biological entities is key to reconstructing the evolution of any group of organisms, in all respects, from the evolution of morphology, development and physiology, by comparing their traits in a phylogenetic framework, to ecological roles and changes, by documenting their temporal and spatial patterns of association and their sedimentary and taphonomic contexts.

Like most complex organisms, plants typically become disaggregated during fossilization as a result of taphonomic processes (uprooting, transport, decomposition). Additionally, in many plants the indeterminately growing plant body sheds parts in programmed ontogenetic processes. The combined result of these conditions is that individual fossil specimens rarely represent the whole plant and most of them are just fragments of it. This precludes a complete understanding of the biological entities – plant species – that most fossil specimens represent. Because of this, one of the immediate goals of palaeobotanical studies is to reconstruct plant species as whole organisms, with as much of their external and internal, vegetative and reproductive traits as possible, based on their fragmentary fossil record. This undertaking is facilitated in plants (unlike in animals), to some extent, by the modular, iterative nature of their construction, which makes it possible to use quantitative descriptors of morphology obtained from direct measurements, calculations and statistics for both reconciling conspecific fragmentary specimens into whole-plant concepts, and ensuring that these concepts encompass the morphological plasticity inherent of natural species, i.e. the whole ranges of variation of the different morphological features observed in the material studied.

Considering their importance, it is not surprising that during the past two centuries palaeobotany has produced a plethora of whole-plant reconstructions. These many reconstructions encompass a very broad range of reliability and detail, as determined by the type and amount of evidence used (the more specimens analysed and the larger and better preserved they are, the more detailed and reliable the reconstruction) and the approaches by which they were produced, from highly speculative ones to those well supported by empirical data and, thus, more objective. The broad variability has several sources that are related primarily to the numbers of fossil specimens available, the mode and quality of their preservation (degree of fragmentation, amount of decay, presence/absence of anatomy) and the way in which they are analysed, but also to the expertise, experience, attention to detail and objectivity of those who analyse them. This combination of factors has resulted, in several cases, in reconstructions being revisited (e.g. *Medullosa*; Andrews, 1945; Pfefferkorn *et al.*, 1984; Wnuk and Pfefferkorn, 1984), sometimes multiple times (e.g. the case of *Prototaxites*; reviewed by Retallack and Landing, 2014).

The reliability (degree of empirical support) and level of detail of whole-plant reconstructions is an important issue that has been rarely discussed. The importance of this issue stems from the fact that, beyond their aesthetic aspect and impact in terms of science communication, whole-plant reconstructions are the most comprehensive representations of extinct species that one can obtain, short of examining live specimens. If they are reliable and detailed, such constructs provide a picture of the species closest to what the organisms looked like while alive, thus bringing that species as close as possible to a biological entity that is directly comparable to living species. In turn, this allows the most accurate and insightful comparisons with living or extinct relatives for taxonomic treatments or for scoring with confidence as many characters as possible for phylogenetic studies; the results of such approaches are crucial for obtaining highly resolved and accurately dated phylogenies, which are, in turn, key for understanding evolutionary patterns (patterns of relationships, ancestral character states, character evolution, evolution of development, etc.). Nowhere are all these more important, perhaps, than in the case of early vascular plants whose sporophytes have simple body plans that provide fewer morphological and anatomical characters than the more derived tracheophytes, for use in taxonomic treatments and phylogenetic studies. A detailed picture of the organism provided by a reliable whole-plant reconstruction brings together characters that cannot all be documented in individual fossils of that species, thus providing an empirical framework for recognizing fragments of a species that lack some of the diagnostic features of that species (Blanco-Moreno *et al.*, 2019). At the same time, the quantitative morphometric descriptors of the species that underpin its whole-plant concept provide a reference benchmark against which to compare specimens of unknown identity, to make empirically supported decisions on their taxonomic affinity (i.e. whether or not they belong to the reconstructed species). Additionally, a well-supported whole-plant concept is also key for a better understanding of different aspects of the plant’s life history, such as physiology or ecology, especially when anatomy is also well documented and in combination with the taphonomy of the fossils.

For all the above reasons, detailed empirically supported whole-plant reconstructions are crucial for palaeobotany (often regarded, along with its animal counterpart, palaeontology, as little more than ‘stamp collecting’ fossils (see also Tomescu, 2016)) to contribute meaningfully, as an equal participant, to understanding of multiple aspects of plant evolution. Thus, it is surprising how few of the studies that introduce whole-plant concepts provide information on the numbers of specimens studied and the methods used to analyse them, on the specifics of the process that led to the published reconstructions or on those aspects of the reconstructions that are better supported by the data vs those aspects that are more speculative. Consequently, in closing, we provide the following tentative set of guidelines for reporting the data and procedures used in assembling empirically supported whole-plant reconstructions.

Report the total number of specimens studied for the reconstruction, as well as specific numbers measured for each variable or type of observation, as applicable.
Provide detailed explanations of how each variable was measured, as needed, including explanations of any approximations that were implemented for incomplete specimens.
Report the raw data for each variable or type of observation.
Explain any calculations used on the raw data to produce derived variables.
Explain the analysis methods used to assess the data for each variable, ideally accompanying them with justification for the choice of method and explanation of the biological significance of the variable, as applicable.
List the concrete steps of the process by which the data were used to construct the whole-plant concept.
Explain how each variable was incorporated, and is reflected, in the whole-plant reconstruction, with as many specifics as possible, as applicable.
Point out the aspects of the whole-plant concept that are incompletely supported by data, that reflect less accurately or more roughly the data, or that are purely speculative, and explain why you chose to depict them the way they are depicted in the whole-plant concept.

The approach we present here to the collection, analysis and interpretation of the *Nowenia* data follows these guidelines, introducing a method for utilizing morphometric data from large numbers of specimens in the construction of a whole-plant concept. We hope that this can provide a model and template for similar future studies addressing early tracheophytes with simple body plans.

## CONCLUSIONS

*Nowenia matsunagae* represents a new genus that is now added to the list of 36 or more known zosterophylls. *Nowenia* plants combined decumbent smooth axes with sparse branching and occasional K-branches, and upright portions with more densely branching smooth axes that bore solitary bivalvate sporangia; dormant branch meristem were frequent along the axes. This morphology reinforces previous knowledge of the characteristic features and body plans of zosterophylls. Parsimony-based phylogenetics places *Nowenia* as sister to *Forania plegiospinosa* (Jensen and Gensel, 2013), from which it differs primarily in the absence of external emergences on the axes. *Nowenia* formed monodominant stands that expanded primarily by vegetative rhizomatous growth in relatively close vicinity to the floodplain depositional environments that preserved the fossil assemblages.

*Nowenia* is only the second plant type characterized in detail from the rich late Lochkovian–Early Pragian fossil assemblages of the Cottonwood Canyon locality in the Early Devonian Beartooth Butte Formation. Alongside another species documented from Cottonwood Canyon, the lycopsid *Sengelia radicans* (Matsunaga and Tomescu, 2017), *Nowenia* joins a very short list of Early Devonian plants that have been characterized based on ample and detailed morphometric analyses of numerous specimens. Among these, *Nowenia* is the only zosterophyll for which an empirically based whole-plant concept that makes explicit and extensive use of quantitative data is available.

The approach we used to reconstruct the *Nowenia* plant introduces a method for utilizing morphometric data to construct whole-plant concepts of early tracheophytes with simple body plans. Future studies applying or expanding on this method could produce data and reconstructions at equivalent levels of detail and accuracy. Such levels of detail and accuracy are sorely needed for as many Early Devonian plants as possible, as they can expand significantly the range of characters that can be compared in plants with relatively simple body plans. Such expanded character lists, which broaden the range of qualitative morphological characters (e.g. variations in branching architecture, growth habit) and allow the inclusion of quantitative characters in both strictly comparative and phylogenetic approaches, are the requisite for conclusive analyses aimed at understanding the relationships of early vascular plants.

## Supplementary Material

mcag040_Supplementary_Data

## SUPPLEMENTARY DATA

Supplementary data are available at *Annals of Botany* online and consist of the following. Table S1: character matrix used to construct the phylogeny in Fig. 15. Table S2: morphometric data on *Nowenia* sporangia. Table S3: data on *Nowenia* axes used to calculate the coefficient of branch subordination (CBS). Table S4: data matrix used in the PCA of *Nowenia* morphology (Fig. 8). Table S5: summary of results of the PCA (Fig. 8): eigenvalues and percentage variance on each PC axis. Table S6: variable loadings on each of the PC axes. Figure S1: *Nowenia wyomingense* gen. et sp. nov., types of preservation. Figure S2: *Nowenia wyomingense* gen. et. sp. nov. colonized by microconchids and showing possible traces of herbivory. Figure S3: two unnamed zosterophyll types from the Cottonwood Canyon locality of the Beartooth Butte Formation (Wyoming). Supplementary Note: description of characters used in the phylogenetic analysis; extended version of Systematics: Remarks: Taxonomic placement – comparison with similar taxa and justification of a new genus; extended version of Discussion: Cuticular anatomy comparisons. Supplementary Data Sheet. Measurements and other data recorded on *Nowenia* specimens.

## References

[mcag040-B1] Andrews HN . 1945. Contributions to our knowledge of American carboniferous floras. VII. Some pteridosperm stems from Iowa. Annals of the Missouri Botanical Garden 32: 323–360. doi:10.2307/2394379

[mcag040-B2] Banks HP . 1968. The early history of land plants. In: Drake ET. ed. Evolution and environment. New Haven: Yale University Press, 73–107.

[mcag040-B3] Banks HP , Davis MR. 1969. *Crenaticaulis*, a new genus of Devonian plants allied to *Zosterophyllum*, and its bearing on the classification of early land plants. American Journal of Botany 56: 436–449. doi:10.1002/j.1537-2197.1969.tb07555.x

[mcag040-B4] Beck CB . 1960. The identity of *Archaeopteris* and *Callixylon*. Brittonia 12: 351–368. doi:10.2307/2805124

[mcag040-B5] Berry CM , Edwards D. 1994. New data on the morphology and anatomy of the Devonian zosterophyll *Serrulacaulis* Hueber and Banks from Venezuela. Review of Palaeobotany and Palynology 81: 141–150. doi:10.1016/0034-6667(94)90104-X

[mcag040-B6] Berry CM , Gensel PG. 2019. Late mid Devonian *Sawdonia* (Zosterophyllopsida) from Venezuela. International Journal of Plant Sciences 180: 540–557. doi:10.1086/702940

[mcag040-B7] Bippus AC , Tomescu AMF. **2017.** Characterizing the Early Devonian plant communities of western North America: the Lochkovian-Pragian Cottonwood Canyon flora of Wyoming. In: *Botany 2017 - Botanical Society of America Annual Meeting Abstracts*. Botanical Society of America. http://2017.botanyconference.org/engine/search/index.php?func=detail&aid=118 (9 March 2026).

[mcag040-B8] Blackstone DL Jr , McGrew PO. **1954.** New occurrence of Devonian rocks in north central Wyoming. In: Richards PW. ed. *Guidebook, Billings Geological Society, 5th Annual Field Conference, September 9–11, 1954*, 38–43.

[mcag040-B9] Blanco-Moreno C , Buscalioni ÁD. 2023. Revision of the Barremian fern *Coniopteris laciniata* from Las Hoyas and El Montsec (Spain): highlighting its importance in the evolution of vegetation during the Early Cretaceous. Taxon 72: 625–637. doi:10.1002/tax.12888

[mcag040-B10] Blanco-Moreno C , Gomez B, Marugán-Lobón J, Daviero-Gomez V, Buscalioni ÁD. 2019. A novel approach for the metric analysis of fern fronds: growth and architecture of the Mesozoic fern *Weichselia reticulata* in the light of modern ferns. PLoS One 14: e0219192. doi:10.1371/journal.pone.0219192 31247026 PMC6597107

[mcag040-B11] Caruso JA , Tomescu AMF. 2012. Microconchid encrusters colonizing land plants: the earliest North American record from the Early Devonian of Wyoming, USA. Lethaia 45: 490–494. doi:10.1111/j.1502-3931.2012.00305.x

[mcag040-B12] Cascales-Miñana B , Meyer-Berthaud B. 2014. Diversity dynamics of Zosterophyllopsida. Lethaia 47: 205–215. doi:10.1111/let.12051

[mcag040-B13] Cavalier-Smith T . 1998. A revised six-kingdom system of life. Biological Reviews 73: 203–266. doi:10.1017/S0006323198005167 9809012

[mcag040-B14] Claisse P , Cascales-Miñana B, Capel E, Tomescu AMF. 2026. Reevaluating the phylogenetic relationships of zosterophylls with a comprehensively sampled dataset and a combination of traditional and new alternative methods. Annals of Botany 137: 1624–1645. doi:10.1093/aob/mcaf146 PMC13274996 40638794

[mcag040-B15] Crepet WL , Niklas KJ. 2019. The evolution of early vascular plant complexity. International Journal of Plant Sciences 180: 800–810. doi:10.1086/705001

[mcag040-B16] DiMichele WA , Hook RW. 1992. Paleozoic terrestrial ecosystems. In: Behrensmeyer AK, Damuth JD, DiMichele WA, Potts R, Sues HD, Wing SL. eds. Terrestrial ecosystems through time. Chicago: University of Chicago Press, 205–325.

[mcag040-B18] Dorf E . 1933. A new occurrence of the oldest known terrestrial vegetation, from Beartooth Butte, Wyoming. Botanical Gazette 95: 240–257. doi:10.1086/334384

[mcag040-B19] Dorf E . 1934. Lower Devonian flora from Beartooth Butte, Wyoming. Geological Society of America Bulletin 45: 425–440. doi:10.1130/GSAB-45-425

[mcag040-B20] Dorn S , Abidi S, Bippus AC, Matsunaga KKS, Tomescu AMF. **2017.** Microconchid-plant interactions in the Early Devonian wetlands of Wyoming (Beartooth Butte Formation, Lochkovian-Pragian). In: *Botany 2017 - Botanical Society of America Annual Meeting Abstracts*. Botanical Society of America. http://2017.botanyconference.org/engine/search/index.php?func=detail&aid=172 (9 March 2026).

[mcag040-B21] Edwards D . 1969a. Further observations on *Zosterophyllum llanoveranum* from the Lower Devonian of South Wales. American Journal of Botany 56: 201–210. doi:10.1002/j.1537-2197.1969.tb07524.x

[mcag040-B22] Edwards D . 1969b. *Zosterophyllum* from the Lower Old Red Sandstone of South Wales. New Phytologist 68: 923–931. doi:10.1111/j.1469-8137.1969.tb06491.x

[mcag040-B23] Edwards D . 1970. Observations on the Lower Devonian plant, *Gosslingia breconensis* Heard. Philosophical Transactions of the Royal Society of London 258: 225–243. doi:10.1098/rstb.1970.0034 22408827

[mcag040-B24] Edwards D . 1975. Some observations on the fertile parts of *Zosterophyllum myretonianum* Penhallow from the Lower Old Red Sandstone of Scotland. Earth and Environmental Science Transactions of The Royal Society of Edinburgh 69: 251–265. doi:10.1017/S0080456800015209

[mcag040-B25] Edwards D . 2003. Embryophytic sporophytes in the Rhynie and Windyfield cherts. Transactions of the Royal Society of Edinburgh: Earth Sciences 94: 397–410. doi:10.1017/S0263593300000778

[mcag040-B26] Edwards D , Kenrick P. 1986. A new zosterophyll from the Lower Devonian of Wales. Botanical Journal of the Linnean Society 92: 269–283. doi:10.1111/j.1095-8339.1986.tb01432.x

[mcag040-B27] Edwards D , Edwards DS, Rayner R. 1982. The cuticle of early vascular plants and its evolutionary significance. In: Cutler DF, Alvin KL, Price CE. eds. The plant cuticle. Cambridge, MA: Academic Press, 341–361.

[mcag040-B28] Edwards D , Kenrick P, Carluccio LM. 1989. A reconsideration of cf. *Psilophyton princeps* (Croft and Lang, 1942), a zosterophyll widespread in the Lower Old Red Sandstone of South Wales. Botanical Journal of the Linnean Society 100: 293–318. doi:10.1111/j.1095-8339.1989.tb01723.x

[mcag040-B29] Edwards D , Kerp H, Hass H. 1998. Stomata in early land plants: an anatomical and ecophysiological approach. Journal of Experimental Botany 49: 255–278. doi:10.1093/jxb/49.Special_Issue.255

[mcag040-B30] Elliott DK , Ilyes RR. 1996. Lower Devonian vertebrate biostratigraphy of the western United States. Modern Geology 20: 253–262.

[mcag040-B31] Elliot DK , Johnson HG. 1997. Use of vertebrates to solve biostratigraphic problems: examples from the Lower and Middle Devonian of Western North America. Geological Society of America Special Paper 321: 179–188. doi:10.1130/0-8137-2321-3.179

[mcag040-B32] Fiorillo AR . **2000.** The ancient environment of the Beartooth Butte Formation (Devonian) in Wyoming and Montana: combining paleontological inquiry with federal management needs. In: McCool SF, Cole DN, Borrie WT, O’Loughlin J, eds. *Wilderness science in a time of change conference, Vol. 3: Wilderness as a place for scientific inquiry, 1999, May 23–27. Missoula, MT.* Washington, DC: USDA Forest Service Proceedings MRS-P-15, 3, 160–167.

[mcag040-B33] Gensel PG , Andrews HN, Forbes WH. 1975. A new species of *Sawdonia* with notes on the origin of microphylls and lateral sporangia. Botanical Gazette 136: 50–62. doi:10.1086/336782

[mcag040-B34] Gensel PG . 1982. *Oricilla*, a new genus referable to the zosterophyllophytes from the late Early Devonian of northern New Brunswick. Review of Palaeobotany and Palynology 37: 345–359. doi:10.1016/0034-6667(82)90007-0

[mcag040-B35] Gensel PG . 1992. Phylogenetic relationships of the zosterophylls and lycopsids: evidence from morphology, paleoecology, and cladistic methods of inference. Annals of the Missouri Botanic Garden 79: 450–473. doi:10.2307/2399750

[mcag040-B36] Gensel PG , Andrews HN. 1984. Plant life in the Devonian. New York: Praeger.

[mcag040-B37] Gensel PG , Berry CM. 2016. Sporangial morphology of the Early Devonian zosterophyll *Sawdonia ornata* from the type locality (Gaspé). International Journal of Plant Sciences 177: 618–632. doi:10.1086/687301

[mcag040-B38] Gensel PG , Milano A, Willoughby A, Belcher J. 2025. A new zosterophyll with novel emergence and cuticle features from the Early Devonian of New Brunswick, Canada. International Journal of Plant Sciences 186: 152–166. doi:10.1086/734304

[mcag040-B39] Gerrienne P . 1988. Early Devonian plant remains from Marchin (north of Dinant Synclinorium, Belgium), I. *Zosterophyllum deciduum* sp. nov. Review of Palaeobotany and Palynology 55: 317–335. doi:10.1016/0034-6667(88)90091-7

[mcag040-B40] Gerrienne P . 1996. Lower Devonian plant remains from Marchin (northern margin of Dinant Synclinorium, Belgium). IV. *Odonax borealis* gen. et sp. nov. Review of Palaeobotany and Palynology 93: 89–106. doi:10.1016/0034-6667(95)00121-2

[mcag040-B41] Goloboff P , Catalano S. 2016. TNT, version 1.5, with a full implementation of phylogenetic morphometrics. Cladistics 32: 221–238. doi:10.1111/cla.12160 34727670

[mcag040-B42] Guo Y , Wang D. 2016. Studies on plant cuticles from the lower–middle Devonian of China. Review of Palaeobotany and Palynology 227: 42–51. doi:10.1016/j.revpalbo.2015.11.007

[mcag040-B43] Hammer O . 2001. PAST: paleontological statistics software package for education and data analysis. Palaeontologia Electronica 4: 4. http://palaeo-electronica.org/2001_1/past/issue1_01.htm

[mcag040-B44] Hao S , Xue J, Guo D, Wang D. 2010. Earliest rooting system and root: shoot ratio from a new *Zosterophyllum* plant. New Phytologist 185: 217–225. doi:10.1111/j.1469-8137.2009.03056.x 19825018

[mcag040-B45] Hotton CL , Hueber FM, Griffing DH, Bridge JS. 2001. Early terrestrial plant environments: an example from the Emsian of Gaspé, Canada. In: Gensel PG, Edwards D, eds. Plants invade the land: evolutionary and environmental perspectives. New York: Columbia University Press, 179–212.

[mcag040-B46] Hueber FM . 1971. Early Devonian land plants from Bathurst Island, District of Franklin. Geological Survey of Canada Paper 71-28: 1–11. doi:10.4095/100691

[mcag040-B47] Hueber FM. 1972. *Rebuchia ovata*, its vegetative morphology and classification with the Zosterophyllophytina. Review of Palaeobotany and Palynology 14: 113–127 doi:10.1016/0034-6667(72)90012-7

[mcag040-B48] Hueber FM , Banks HP. 1979. *Serrulacaulis furcatus* gen. sp. nov., a new zosterophyll from the lower Upper Devonian of New York state. Review of Palaeobotany and Palynology 28: 169–189. doi:10.1016/0034-6667(79)90008-3

[mcag040-B49] Jensen D , Gensel PG. 2013. *Forania plegiospinosa*, gen. et sp. nov.: a zosterophyll from the Early Devonian of New Brunswick, Canada, with a novel emergence type. International Journal of Plant Sciences 174: 687–701. doi:10.1086/669914

[mcag040-B50] Kenrick P , Edwards D. 1988. A new zosterophyll from a recently discovered exposure of the Lower Devonian Senni beds in Dyfed, Wales. Botanical Journal of the Linnean Society 98: 97–115. doi:10.1111/j.1095-8339.1988.tb01698.x

[mcag040-B51] Kenrick P , Crane PR. 1991. Water-conducting cells in early fossil land plants: implications for the early evolution of tracheophytes. Botanical Gazette 152: 335–356. doi:10.1086/337897

[mcag040-B52] Kenrick P , Crane PR. 1997. The origin and diversification of land plants: a cladistics study. Washington, DC: Smithsonian Institution Press.

[mcag040-B53] Kotyk ME . **1998.** *Late Silurian and Early Devonian fossil plants of Bathurst Island, arctic Canada*. MSc Thesis, University of Saskatchewan, Canada.

[mcag040-B54] Lamsdell JC , Legg DA. 2010. An isolated pterygotid ramus (Chelicerata: Eurypterida) from the Devonian Beartooth Butte Formation, Wyoming. Journal of Paleontology 84: 1206–1208. doi:10.1666/10-040.1

[mcag040-B55] Lamsdell JC , Selden PA. 2013. Babes in the wood – a unique window into sea scorpion ontogeny. BMC Evolutionary Biology 13: 98. doi:10.1186/1471-2148-13-98 23663507 PMC3679797

[mcag040-B56] Lomax BH , Hilton J, Bateman RM, et al 2014. Reconstructing the relative genome size of vascular plants through geological time. New Phytologist 201: 636–644. doi:10.1111/nph.12523 24117890

[mcag040-B57] Lyon AG , Edwards D. 1991. The first zosterophyll from the lower Devonian Rhynie Chert, Aberdeenshire. Transactions of the Royal Society of Edinburgh: Earth Sciences 82: 324–332. doi:10.1017/S0263593300004193

[mcag040-B58] Matsunaga KKS , Tomescu AMF. 2016. Root evolution at the base of the lycophyte clade: insights from an Early Devonian lycophyte. Annals of Botany 117: 585–598. doi:10.1093/aob/mcw006 PMC4817433 26921730

[mcag040-B59] Matsunaga KKS , Tomescu AMF. 2017. An organismal concept for *Sengelia radicans* gen. et sp. nov. – morphology and natural history of an Early Devonian lycophyte. Annals of Botany 119: 1097–1113. doi:10.1093/aob/mcw277 28334100 PMC5604611

[mcag040-B60] Nibbelink M , Tomescu AMF. 2022. Exploring zosterophyll relationships within a more broadly sampled character space: a focus on anatomy. International Journal of Plant Sciences 183: 535–547. doi:10.1086/720384

[mcag040-B61] Noetinger S , Bippus AC, Tomescu AMF. 2021. Palynology of a short sequence of the lower Devonian Beartooth Butte Formation at Cottonwood Canyon (Wyoming): age, depositional environments and plant diversity. Papers in Palaeontology 7: 2183–2204. doi:10.1002/spp2.1395

[mcag040-B62] Oliver FW , Scott DH. 1905. On the structure of the palaeozoic seed *Lagenostoma lomaxi*, with the statement of evidence upon which it is referred to *Lygninodendron*. Philosophical Transactions of the Royal Society of London B: Biological Sciences 197: 193–247. doi:10.1098/rstb.1905.0008

[mcag040-B63] Penhallow DP . 1892. Additional notes on Devonian plants from Scotland. Canadian Record of Science 5: I. http://biodiversitylibrary.org/page/33900903

[mcag040-B64] Pfefferkorn HW , Gillespie WH, Resnick DA, Scheihing MH. 1984. Reconstruction and architecture of medullosan pteridosperms (Pennsylvanian). The Mosasaur 2: 1–8. https://repository.upenn.edu/entities/publication/2cd9dd3a-98c1-4c77-a019-3e8f58e9db71

[mcag040-B65] Poschmann M , Gossmann R, Matsunaga KKS, Tomescu AMF. 2020. Characterizing the branching architecture of drepanophycalean lycophytes (Lycopsida): an exceptional specimen from the Early Devonian Hunsrück Slate, southwest Germany, and its paleobiological implications. Palaontologische Zeitschrift 94: 1–16. doi:10.1007/s12542-018-00443-w

[mcag040-B66] Rayner RJ . 1983. New observations on *Sawdonia ornata* from Scotland. Transactions of the Royal Society of Edinburgh: Earth Sciences 74: 79–93. doi:10.1017/S026359330001018X

[mcag040-B67] Retallack GJ , Landing E. 2014. Affinities and architecture of Devonian trunks of *Prototaxites loganii*. Mycologia 106: 1143–1158. doi:10.3852/13-390 24990121

[mcag040-B68] Rex GM , Chaloner WG. 1983. The experimental formation of plant compression fossils. Palaeontology 26: 231–252. https://www.biodiversitylibrary.org/page/49741184#page/255/mode/1up

[mcag040-B69] Sandberg CA . 1961. Widespread Beartooth Butte Formation of Early Devonian age in Montana and Wyoming and its paleogeographic significance. Bulletin of the American Association of Petroleum Geologists 45: 1301–1309. doi:10.1306/BC7436E1-16BE-11D7-8645000102C1865D

[mcag040-B70] Sandberg CA. 1967. Measured sections of Devonian rocks in northern Wyoming. Geological Survey of Wyoming Bulletin No. 52. Laramie:University of Wyoming.

[mcag040-B71] Speck T , Vogellehner D. 1988. Biophysical examinations of the bending stability of various stele types and the upright axes of early “vascular” land plants. Botanica Acta 101: 262–268. doi:10.1111/j.1438-8677.1988.tb00042.x

[mcag040-B72] Steenbock CM , Tomescu AM. 2013. Resurrecting *Sphondylophyton* as a rhodophyte alga from the Early Devonian. International Journal of Plant Sciences 174: 1171–1181. doi:10.1086/671806

[mcag040-B73] Stewart WN , Rothwell GW. 1993. Paleobotany and the evolution of plants. Cambridge: Cambridge University Press.

[mcag040-B74] Sun TX , Edwards D, Li CS. 2005. The stomatal apparatus of *Lycopodium japonicum* and its bearing on the stomata of the Devonian lycophyte *Drepanophycus spinaeformis*. Botanical Journal of the Linnean Society 149: 209–216. doi:10.1111/j.1095-8339.2005.00434.x

[mcag040-B75] Surapaneni VA , Bold G, Speck T, Thielen M. 2020. Spatio-temporal development of cuticular ridges on leaf surfaces of *Hevea brasiliensis* alters insect attachment. Royal Society Open Science 7: 201319. doi:10.1098/rsos.201319 33391807 PMC7735362

[mcag040-B76] Tanner WR . 1982. A new species of *Gosslingia* (Zosterophyllophytina) from the lower Devonian Beartooth Butte Formation of northern Wyoming. Third North American Paleontological Convention 2: 541–546.

[mcag040-B77] Tanner WR . **1983.** *A fossil flora from the Beartooth Butte Formation of Wyoming*. PhD Thesis, Southern Illinois University, USA.

[mcag040-B78] Taylor PD , Vinn O. 2006. Convergent morphology in small spiral worm tubes (*‘Spirorbis’*) and its palaeoenvironmental implications. Journal of the Geological Society 163: 225–228. doi:10.1144/0016-764905-145

[mcag040-B79] Tomescu AMF . 2016. Development: paleobotany at the high table of evo-devo. Current Biology 26: R505–R508. doi:10.1016/j.cub.2016.05.001 27326713

[mcag040-B80] Vinn O . 2006. Two new microconchid (Tentaculita Boucek, 1964) genera from the Early Palaeozoic of Baltoscandia and England. Neues Jahrbuch Fur Geologie und Paläontologie Monatshefte 2006: 89–100. doi:10.1127/njgpm/2006/2006/89

[mcag040-B81] Wand MP . 1997. Data-based choice of histogram bin width. The American Statistician 51: 59–64. doi:10.1080/00031305.1997.10473591

[mcag040-B82] Wang DM , Hao SG, Wang Q. 2003. *Hsüa deflexa* sp. nov. from the Xujiachong Formation (Lower Devonian) of eastern Yunnan, China. Botanical Journal of the Linnean Society 142: 255–271. doi:10.1046/j.1095-8339.2003.00187.x

[mcag040-B83] Wnuk C , Pfefferkorn HW. 1984. The life habits and paleoecology of middle Pennsylvanian medullosan pteridosperms based on an *in situ* assemblage from the Bernice Basin (Sullivan County, Pennsylvania, U.S.A.). Review of Palaeobotany and Palynology 41: 329–351. doi:10.1016/0034-6667(84)90053-8

[mcag040-B84] Xu H-H . 2011. Re-examination of specimens attributed to *Sawdonia curstipa* Wang and Hao (zosterophyll) from the Middle Devonian of Xinjiang, China. Palaeoworld 20: 357–361. doi:10.1016/j.palwor.2011.06.003

[mcag040-B85] Zdebska D . 1982. A new zosterophyll from the Lower Devonian of Poland. Palaeontology 25: 247–263. https://www.biodiversitylibrary.org/page/49707967#page/265/mode/1up

