# Supplementary material for: A morphometrics-informed reconstruction of the Early Devonian zosterophyll Nowenia matsunagae gen. et sp. nov. as a template for building detailed empirically supported whole-plant concepts of early tracheophytes with simple body plans
Source: Ann Bot. 2026 Feb 25;137(6):1658–88. doi: 10.1093/aob/mcag040 (PMC13274987; doi:10.1093/aob/mcag040)
Supplement: mcag040_Supplementary_Data [file mcag040_supplementary_data.zip › ElAbdallahEtAl2025_R1_SupplementaryNote.docx]

**Building detailed and accurate whole-plant concepts: a morphometrics-informed reconstruction of the zosterophyll *Nowenia matsunagae* gen. et sp. nov. from the Lower Devonian of Wyoming**

Samar R. El-Abdallah^1^, Penelope Claisse^2^, Candela Blanco-Moreno^3^, Alexandru M.F. Tomescu^1^*

^1^ Department of Biological Sciences, California State Polytechnic University, Humboldt, Arcata, California 95521, USA

^2^ Evo-Eco-Paléo, École doctorale Sciences de la matière, du rayonnement et de l'environnement, Université de Lille, 59000 Lille, France

^3^ Departamento de Biología, Facultad de Ciencias, Universidad Autónoma de Madrid, 28049 Madrid, Spain

* Author for correspondence: [mihai@humboldt.edu](mailto:mihai@humboldt.edu)

SUPPLEMENTARY NOTE

**Characters used in the phylogenetic analysis**

1. Thallus holding sporophyte axes

This refers to instances when sporophytes are attached to a thallus of presumed gametophytic nature.

(0) Absence

(1) Presence

2. Rhizomatous portions of the sporophyte: shape [New]

This character distinguished horneophytopsids, which have a cormose base, from taxa with axial rhizomes.

(0) Axial

(1) Lobed

3. Rooting system: positively geotropic axes

(0) Absence

(1) Presence

4. K-branching or H-branching

(0) Absence

(1) Presence

5. Branching type of erect axes

(0) Isotomous

(1) Pseudomonopodial

(2) Not branching

6. Branching geometry

(0) Planar

(1) Three-dimensional

(2) Branching absent

7. Branching pattern

(0) Planar-alternate

(1) Helical or irregular

(2) Branching absent

8. Branching angle

(0) Narrow to wide

(1) Branches parallel to subtending axis (U-patterned branching)

9. Circinate axis tips

(0) Absence

(1) Presence

10. Subaxillary branching (and subaxillary tubercles)

(0) Absence

(1) Presence

11. Emergences

Emergences are recognized as unvascularized outgrowths on the surface of the plant.

(0) Absence

(1) Presence

12. Emergences: localization

(0) Vegetative parts only

(1) Vegetative and fertile parts

(2) Fertile parts only (sporangium and stalk)

13. Emergences: biseriate arrangement

(0) Absence

(1) Presence

14. Glandular structures on emergences

Refers to emergences with rounded, darkened or missing tips, which are interpreted as glandular in nature.

(0) Absence

(1) Presence

15. Sporangium emergences: position

(0) Emergences absent on sporangium

(1) On abaxial valve

(2) On both valves

16. Emergences: shape

(0) Rounded

(1) Conical

(2) Shelf-like

17. Lateral sporangia: attachment: cauline

(0) On axis

(1) On specialized appendage

18. Sporangium: position on sporophyte axis.

(0) Terminal

(1) Lateral

19. Sporangium stalk size: relative to sporangium size

(0) Sessile

(1) Short (stalk length < sporangium size)

(2) Long (stalk length ≥ sporangium size)

20. Sporangium stalk stoutness

(0) Sporangium length/width < 1

(1) Sporangium length/width > 1

21. Vascular bundle suppling sporangium ("-" if sessile sporangium)

(0) Absence

(1) Presence

22. Stalk or sporangium (if sessile): orientation [New]

(0) At acute angle to subtending axis

(1) At right angle to subtending axis

23. Sporangium: shape

(0) Approximately rounded

(1) Approximately ellipsoidal

(2) Reniform

(3) Trumpet-shaped (*Cooksonia*)

(4) Lenticular vertically flattened

(5) Cylindrical

24. Sporangium: symmetry

(0) Radially symmetrical

(1) Bilaterally symmetrical

25. Sporangium: dorsiventral flattening

(0) Absence

(1) Presence

26. Sporangium: dehiscence

(0) Terminal

(1) Along distal margin

(2) By a lateral line

(3) Basal

27. Sporangium: dehiscence line thickening

(0) Absence

(1) Presence

28. Sporangium: relative valve size (scored "-" if not bivalvate/dehiscence along distal margin)

(0) Isovalvate

(1) Anisovalvate, abaxial valve bigger than adaxial valve

(2) Anisovalvate, valve smaller than adaxial valve

29. Sporangium: orientation with respect to subtending axis

(0) Lateral (pointing outwards)

(1) Pointing more or less apically

(2) Curved toward the axis

30. Sporangium: paired

This character distinguishes euphyllophytes like *Psilophyton* from the other groups.

(0) Absence

(1) Presence

31. Sporangium: distribution by order of branching

(0) Sporangia only on the highest order branches

(1) Sporangia on highest order and lower orders of branching (or if occurring between branching points

32. Sporangium: grouping

Absence refers to solitary sporangia scattered along axes. Character not applicable for taxa with terminal sporangia.

(0) Absence

(1) Presence

33. Grouped sporangia: distribution

(0) Terminal, forming a strobilus

(1) Intercalary

34. Grouped sporangia: density in groups

(0) Lax

(1) Compact

35. Strobilus: position (scores "-" if no strobilus)

Scored "?" if only strobili are known for the species.

(0) On main axis only

(1) On lateral branches only

36. Sporangium: taxis

(0) Helical or irregular

(1) In rows

37. Rowed sporangia: position of rows (scored "-" if sporangia have helical taxis)

(0) Opposite

(1) Alternate

38. Rowed sporangia: number of rows

(0) One or two

(1) More than two

(2) Always two

39. Leaves

(0) Presence

(1) Absence

40. Spore size

(0) Homosporous

(1) Heterosporous/anisosporous

41. Protostele: shape

(0) Terete

(1) Elliptical

(2) Lobed

42. Pattern of primary xylem maturation

(0) Xylem absent

(1) Centrarch

(2) Exarch

(3) No conspicuous pattern of maturation

(4) Mesarch

43. Metaxylem secondary wall thickening

(0) Absent

(1) Scalariform

(2) Pitted

44. Metaxylem pitting (only applicable for metaxylem with pitted tracheids)

(0) S-type

(1) C-type

(2) G-type

(3) P-type

45. Sterome

Refers to outermost layer(s) of cells with thickened walls.

(0) Absence

(1) Presence

**Extended version of Systematics: Remarks:** *Taxonomic placement – comparison with similar taxa and justification of a new genus*

Taken together, the phylogenetic position of the new plant and the morphological comparisons discussed below justify the erection of a new zosterophyll genus.

An exarch protostele, a diagnostic character of zosterophylls (Banks, 1968), cannot be ascertained in *Nowenia*, due to its preservation as adpressions. However, *Nowenia* possesses reniform sporangia attached laterally on axes and dehiscing along their distal margin, two other diagnostic features of zosterophylls (Banks, 1968), which support its placement in class Zosterophyllopsida (*sensu* Kenrick and Crane, 1997). *Nowenia* possesses additional features documented in zosterophylls and not usually seen in other groups of early tracheophytes: K- or H-branching and rosette cellular patterns in the epidermis (Edwards *et al*., 1982; Gensel and Andrews, 1984; Gensel, 1992; Kenrick and Crane, 1997).

Phylogenetic analyses place *Nowenia* in a clade with 13 other zosterophyll genera (Fig. 15, Supplementary Table 1). Of these, *Trichopherophyton*, known from permineralizations (Lyon and Edwards, 1991), lacks information on many characters that would allow in-depth comparisons to *Nowenia*. Five of the genera – *Crenaticaulis* (Banks and Davis, 1969), *Deheubarthia* (Edwards *et al.*, 1989), *Gosslingia* (Edwards, 1970), *Odonax* (Gerrienne, 1996) and *Thrinkophyton* (Kenrick and Edwards, 1988) – possess two features that are key in zosterophyll taxonomy: subaxillary tubercles and sporangia grouped in fertile zones (which form terminal spikes in *Odonax*) (Supplementary Table 1), both of which are absent in *Nowenia*, thus excluding these genera from taxonomic discussions. Additionally, four of them – *Crenaticaulis*, *Gosslingia*, *Thrinkophyton* and *Deheubarthia* – lack the U-patterned branching seen in *Nowenia* (also present in *Odonax*).

*Sawdonia* (Hueber, 1971b; Gensel *et al*., 1975; Rayner, 1983; Gensel and Berry, 2016) and *Serrulacaulis* (Hueber and Banks, 1979; Berry and Edwards, 1994; Xu *et al*., 2011) differ from *Nowenia* in possessing grouped sporangia and in the morphology of their sporangia, with abaxial valves larger than the adaxial ones (Supplementary Table 1). Additionally, *Serrulacaulis* lacks the U-patterned branching present in *Nowenia* and has a thickened line of sporangial dehiscence, unlike *Nowenia*.

*Konioria* (Zdebska, 1982) shares the highest number of characters with *Nowenia*, alongside *Forania*, *Zosterophyllum* and *Odonax* (Supplementary Table 1). However, *Konioria* lacks the U-patterned branching and has a distinctive placement of sporangia – exclusively at branching points of axes. Additionally, the phylogenetic analysis does not support a close relationship of *Konioria* to *Nowenia* (Fig. 15).

The close relationships supported by the phylogenetic analysis between *Nowenia* and *Oricilla*, *Tarella*, *Zosterophyllum* and *Forania* (Fig. 15) require closer scrutiny. *Oricilla* (Gensel, 1982) and *Tarella* (Edwards and Kenrick, 1986) share many characters with *Nowenia* (Table 2). However, unlike *Nowenia* they have sporangia arranged in fertile zones and they also differ in the orientation of sporangia and the sporangial stalks. Additionally, *Oricilla* lacks dormant branch meristems and, because its basal portions are not known, it is unknown whether it possessed K-branching. All these diagnostic differences belie the apparent strong morphological similarity between *Oricilla* and *Tarella*, and *Nowenia*. Interestingly, *Oricilla* is similar to *Nowenia* in the patterning of epidermal cells, which form rosettes (Gensel, 1982), but this feature is also shared by *Sawdonia* (Edwards *et al.*, 1982). The genus *Zosterophyllum* includes many species, all of which differ from *Nowenia* in having sporangia grouped into fertile zones (Table 2), in one or more rows (e.g., Edwards, 1969b; Gensel, 1982b) and some forming strobili (Edwards, 1969a; 1975).

A zosterophyll genus that does not possess grouped sporangia and is placed as sister to *Nowenia* in the phylogeny (Fig. 15) is *Forania* (Jensen and Gensel, 2013). *Forania* shares many features with the *Nowenia* (Table 2), including dormant branch meristems and U-patterned branching. Like *Nowenia*, *Forania* lacks subaxillary tubercles and may have had K-branching, and its architecture is interpreted to include both decumbent and upright axes. A fine point of difference between *Nowenia* and *Forania* concerns the sporangial dehiscence line, which is described as thickened in the latter (and scored as such in the phylogenetic matrix). The line of dehiscence observed in cuticular material of *Nowenia* is very similar to that illustrated in *Forania* cuticular material. However, based on our examination of both the cuticular material of *Nowenia* sporangia (Fig. 11A. B) and the sporangia exposed on bedding planes (Fig. 10D, F), we exclude the possibility of a thickened dehiscence line being present. A more significant difference is that unlike *Nowenia*, *Forania* has anisotomous branching. Additionally, in *Forania* the undeveloped dormant branches are coiled abaxially rather than adaxially and *Forania* axes bear two rows of large multicellular spinescent projections with putative secretory function (Jensen and Gensel, 2013). These differences, and especially the presence in *Forania* of large spinescent projections that are diagnostic at the generic level in zosterophylls (Jensen and Gensel, 2013), exclude *Forania* from consideration as a possible taxonomic placement of the new zosterophyll.

**Extended version of Discussion:** *Cuticular anatomy comparisons*.

Three types of epidermal features documented in cuticular material of *Nowenia* merit discussion: the surface sculpturing of epidermal cells consisting of a central papilla and radiating ridges; the groups of cells forming rosette patterns; and the stomata.

The epidermal cells of *Nowenia* that bear surface sculpturing are similar to those of *Sawdonia ornata*. In *Sawdonia*, Edwards *et al*. (1982) and Rayner (1983) demonstrated that both the darker central area (papilla) and the ridges that radiate from it consist of thickened cuticle and, thus, represent features of the living plant (Edwards *et al*., 1982). Given their strong similarity, it is reasonable to assume that the surface features of *Nowenia* cells are also cuticle thickenings.

In *Sawdonia*, Rayner (1983), referencing the findings of Haberlandt (1914) and Kay *et al*. (1981), hypothesized that the papillae may have had optical roles – either reflecting light away from the plant or refracting it into the epidermal cells in specific ways. However, since illumination conditions were the same for all the plants in the communities that included *Nowenia*, we would expect to see such optical adaptations in other plant types of the Beartooth Butte Formation flora – something that has yet to be tested.

An alternative adaptive explanation for the papillae that includes the papillae and thickened radiating ridges is that they served as water retention enhancers. In bryophytes, Dilks and Proctor (1979) and Glime (2017) suggested that papillae and other structural intricacies of the epidermal cell surface are useful for capillary water storage, as every little groove can hold a water droplet. Along the same lines, Glime (2017) further demonstrated that capillary water layers retained around the papillae enhance light transmission through the cuticle, thus serving both optical and water retention functions. It is possible that the papillae and radiating ridges of *Nowenia* (and *Sawdonia*) had similar functions. Furthermore, it is possible that these asperities enhanced water condensation on the surface of the plant. Elaborating on this observation, one could speculate that the ridges may have functioned in the plants’ self-irrigation, by guiding capillary water to concentrate it around the papillae, where it would form larger drops that would humidify the substrate immediately beneath the plant.

Another possible explanation proposed by Rayner (1983) for the radiating ridges of cuticle is that they are taphonomic products – the results of shriveling of the epidermal cells that originally had convex outer periclinal walls. In this case, the presence of a central papilla would constrain the convex cuticle around it to generate radially arranged folds. This hypothesis could be tested by accurate measurements of the ridges that should, in this case, be thicker centrally and taper toward the cell periphery. A third adaptive explanation of the ridges is that they worked, along with the papillae, in hindering potential herbivorous arthropods from walking along, or attaching their eggs to, the epidermal surface, as has been demonstrated for *Hevea brasiliensis* by Surapaneni *et al*. (2020).

The fact that the cells bearing papillae and ridges are not found on all cuticular fragments recovered from *Nowenia* specimens and, thus, seem to be restricted to some parts of the plant (as also noted in *Sawdonia ornata*; Edwards *et al*., 1982), would be consistent with a function of these structures in enhancing the plants’ irrigation, especially if they were present preferentially on downward-facing sides of the axes. Conversely, if their role was mainly to enhance light transmission, then they would be expected to be absent from downward-facing sides of the axes. However, exploring these hypotheses will necessitate future studies of the detailed distribution patterns of cells bearing papillae and ridges on the plants.

Some of the cells of the epidermis of *Nowenia* form rosette patterns, a feature also encountered in at least five or six other zosterophylls: *Serrulacaulis* (Hueber and Banks, 1979), *Sawdonia ornata* (Edwards *et al*., 1982; Rayner, 1983), *Deheubarthia* (Edwards *et al*., 1989), *Oricilla* (Gensel, 1982), *Trichopherophyton* (Lyon and Edwards, 1991), and *Staphylophyton* (Gensel *et al.*, 2025). Several interpretations have been proposed for these rosettes based on their structure. The central cell was first deemed to represent remnants of a trichome because of similarity with such structures from extant plants (Edwards *et a*l., 1982; Gensel, 1992). Hueber and Banks (1979), in their description of *Serrulacaulis furcatus*, proposed that the central cell may have had secretory or storage functions. Based on SEM observations in *Sawdonia ornata*, which demonstrated the cuticle associated with the central cell to be thinner than the cuticle of the surrounding cells, Edwards *et al*. (1982) suggested that the former may have been concerned with water relations. However, Lyon and Edwards (1991) provided direct evidence of small trichomes whose bases occupied the central location in the epidermal cell rosettes of *Trichopherophyton*. If the epidermal cell rosettes of *Nowenia* also mark the location of trichome bases, the trichomes have yet to be found, although if they were as fine as those of *Trichopherophyton* (which is likely considering the similar cell sizes), they may not have withstood the taphonomic conditions that affected the *Nowenia* fossils.

The preservation of the cuticle around the stomata of *Nowenia* (Fig. 13A, 13B) does not allow for observing the organization of the epidermal cells around the guard cells. Considering that zosterophyll stomata are typically anomocytic (Guo and Wang, 2016), the stomata of *Nowenia* may also be anomocytic. Early Devonian plants have exceptionally large stomatal guard cells (22–86 µm; Lomax *et al*., 2014). *Nowenia* fits this pattern; in fact, with guard cells ca. 95 µm long, its stomata surpass slightly and extend the range of previously measured Early Devonian stomata (previously the longest guard cells – 86 µm – had been documented in *Horneophyton lignieri*; Edwards *et al*., 1998).

LITERATURE CITED

Banks HP. 1968. The early history of land plants. In: Drake ET (ed.) *Evolution and environment.* New Haven, Connecticut: Yale University Press, 73–107.

Banks HP, Davis MR. 1969. *Crenaticaulis*, a new genus of Devonian plants allied to *Zosterophyllum*, and its bearing on the classification of early land plants. *American Journal of Botany* 56: 436–449.

Beck CB. 1960. The identity of *Archaeopteris* and *Callixylon*. *Brittonia* 12: 351–368*.*

Beaulieu JM, Leitch IJ, Patel S, Pendharkar A, Knight CA. 2008. Genome size is a strong predictor of cell size and stomatal density in angiosperms. *New Phytologist* 179: 975–986.

Berry CM, Edwards D. 1994. New data on the morphology and anatomy of the Devonian zosterophyll *Serrulacaulis* Hueber and Banks from Venezuela. *Review of Palaeobotany and Palynology* 81: 141–150.

Berry CM, Gensel PG. 2019. Late Mid Devonian *Sawdonia* (Zosterophyllopsida) from Venezuela.*International Journal of Plant Sciences* 180: 540-557.

Bippus AC, Tomescu AMF. 2017. Characterizing the Early Devonian plant communities of western North America: the Lochkovian-Pragian Cottonwood Canyon flora of Wyoming*. Botanical Society of America annual meeting abstracts*: http://2017.botanyconference.org/engine/search/index.php?func=detail&aid=118

Blackstone DL Jr., McGrew PO. 1954. *New occurrence of Devonian rocks in north central Wyoming.* Billings, Geological Society, 5th Annual Field conference guidebook.

Blanco‐Moreno C, Buscalioni, ÁD.2023. Revision of the Barremian fern *Coniopteris* *laciniata* from Las Hoyas and El Montsec (Spain): Highlighting its importance in the evolution of vegetation during the Early Cretaceous. *Taxon*. 72: 624 – 637.

Blanco-Moreno C, Gomez B, Marugán-Lobón J, Daviero-Gomez V, Buscalioni ÁD 2019. A novel approach for the metric analysis of fern fronds: growth and architecture of the Mesozoic fern *Weichselia reticulata* in the light of modern ferns. *PLoS One* 14: e0219192.

Caruso JA, Tomescu AMF. 2012. Microconchid encrusters colonizing land plants: the earliest North American record from the Early Devonian of Wyoming, USA. *Lethaia* 45: 490–494.

Cascales-Miñana B, Meyer-Berthaud B. 2014. Diversity dynamics of Zosterophyllopsida. *Lethaia* 47: 205–215.

Cavalier-Smith T. 1998. A revised six-kingdom system of life. *Biological Reviews* 73: 203–266.

Claisse P, Cascales-Miñana B, Capel E, Tomescu AMF, 2025. Reevaluating the phylogenetic relationships of zosterophylls with a comprehensively sampled dataset and a combination of traditional and new alternative methods. *Annals of Botany* (in press).

Crepet WL, Niklas KJ. 2019. The evolution of early vascular plant complexity. *International Journal of Plant Sciences* 180: 800–810.

Dilks TJK, Proctor, MCF. 1979. Photosynthesis, respiration and water content in bryophytes. *New Phytologist* 82: 97–114.

DiMichele WA, Hook RW. 1992. Paleozoic terrestrial ecosystems. In: Behrensmeyer AK, Damuth JD, DiMichele WA, Potts R, Sues HD, Wing SL, eds. *Terrestrial ecosystems through time*. Chicago, USA: University of Chicago Press, 205–325.

Dorf E. 1933. A new occurrence of the oldest known terrestrial vegetation, from Beartooth Butte, Wyoming. *Botanical Gazette* 95: 240–57.

Dorf E. 1934. Lower Devonian flora from Beartooth Butte, Wyoming. G*eological Society of America Bulletin* 45: 425–440.

Doran JB. 1980. A new species of *Psilophyton* from the Lower Devonian of northern New Brunswick, Canada. *Canadian Journal of Botany* 58: 2241–2262.

Dorn S, Abidi S, Bippus AC, Matsunaga KKS, Tomescu AMF. 2017. Microconchid-plant interactions in the Early Devonian wetlands of Wyoming (Beartooth Butte Formation, Lochkovian-Pragian). *Botanical Society of America annual meeting abstracts*: http://2017.botanyconference.org/engine/search/index.php?func=detail&aid=172

Edwards D. 1969a. Further observations on *Zosterophyllum llanoveranum* from the Lower Devonian of South Wales. *American Journal of Botany* 56: 201–210.

Edwards D. 1969b. *Zosterophyllum* from the lower Old Red Sandstone of South Wales. *New Phytologist* 68: 923–931.

Edwards D. 1970. Observations on the Lower Devonian Plant, *Gosslingia breconensis* Heard. *Philosophical Transactions of the Royal Society of London* 258: 225–243.

Edwards D. 1975. Some observations on the fertile parts of *Zosterophyllum myretonianum* Penhallow from the lower Old Red Sandstone of Scotland. *Earth and Environmental Science Transactions of The Royal Society of Edinburgh* 69: 251–265.

Edwards D. 2004. Embryophytic sporophytes in the Rhynie and Windyfield cherts. *Transactions of the Royal Society of Edinburgh: Earth Sciences* 94: 397–410.

Edwards D, Kenrick P. 1986. A new zosterophyll from the Lower Devonian of Wales. *Botanical Journal of the Linnean Society* 92: 269–283.

Edwards D, Richardson JB. 2000. Progress in reconstructing vegetation on the Old Red Sandstone continent: two *Emphanisporites* producers from the Lochkovian sequence of the Welsh Borderland. *Geological Society, London, Special Publication* 180: 355–370.

Edwards D, Edwards DS, Rayner R. 1982. The cuticle of early vascular plants and its evolutionary significance. In: Cutler DF, Alvin KL, Price CE, eds. *The plant cuticle*. Cambridge, MA: Academic Press. 341–361.

Edwards D, Kenrick P, Carluccio LM. 1989 A reconsideration of cf. *Psilophyton princeps* (Croft and Lang, 1942), a zosterophyll widespread in the lower Old Red Sandstone of South Wales. *Botanical Journal of the Linnean Society* 100: 293–318.

Edwards D, Kerp H, Hass H. 1998. Stomata in early land plants: an anatomical and ecophysiological approach. *Journal of Experimental Botany* 49: 255–278.

Elliott DK, Ilyes RR. 1996. Lower Devonian vertebrate biostratigraphy of the western United States. *Modern Geology* 20: 253–262.

Elliot DK, Johnson HG. 1997. Use of vertebrates to solve biostratigraphic problems: examples from the Lower and Middle Devonian of Western North America. *Geological Society of America Special Paper* 321: 179–188.

Fiorillo AR. 2000. The ancient environment of the Beartooth Butte Formation (Devonian) in Wyoming and Montana: combining paleontological inquiry with federal management needs. In: McCool SF, Cole DN, Borrie WT, O’Loughlin J, eds. *Wilderness science in a time of change conference, Vol. 3: Wilderness as a place for scientific inquiry; 1999 May 23–27; Missoula, MT. USDA Forest Service Proceedings MRS-P-15* 3: 160–167.

Gensel PG. 1982. *Oricilla*, a new genus referable to the zosterophyllophytes from the late Early Devonian of northern New Brunswick. *Review of Palaeobotany and Palynology* 37: 345–359.

Gensel PG. 1992. Phylogenetic relationships of the zosterophylls and lycopsids: evidence from morphology, paleoecology, and cladistic methods of inference. *Annals of the Missouri Botanic Garden* 79: 450–473.

Gensel PG, Andrews HN. 1984. *Plant life in the Devonian*. New York: Praeger.

Gensel PG, Berry CM. 2016. Sporangial morphology of the early Devonian zosterophyll *Sawdonia ornata* from the type locality (Gaspé). *International Journal of Plant Sciences* 177: 618–632.

Gensel PG, Andrews HN, Forbes W.H. 1975. A new species of *Sawdonia* with notes on the origin of microphylls and lateral sporangia. *Botanical Gazette* 136: 50–62.

Gensel PG, Milano A, Willoughby A, Belcher J. 2025. A new zosterophyll with novel emergence and cuticle features from the Early Devonian of New Brunswick, Canada. *International Journal of Plant Sciences* 186: 152-166.

Gerrienne P. 1996. Lower Devonian plant remains from Marchin (northern margin of Dinant Synclinorium, Belgium). IV. *Odonax borealis* gen. et sp. nov.. *Review of Palaeobotany and Palynology* 93: 89–106.

Gerrienne P. 1988. Early Devonian plant remains from Marchin (north of Dinant Synclinorium, Belgium), I. *Zosterophyllum* *deciduum* sp. nov.. *Review of Palaeobotany and Palynology*. *55*: 317-335.

Glime JM. 2017. Water relations: leaf strategies – structural. In: Glime JM. *Bryophyte ecology. Volume 1. 7-4a-1 Physiological ecology*. Houghton, MI: Michigan Technological University and International Association of Bryologists.

Goloboff P, S Catalano 2016 TNT, version 1.5, with a full implementation of phylogenetic morphometrics. Cladistics.

Guo Y, Wang D. 2016. Studies on plant cuticles from the Lower–Middle Devonian of China. *Review of Palaeobotany and Palynology* 227: 42–51.

Hao S. Xue J, Guo D, Wang D. 2010. Earliest rooting system and root: shoot ratio from a new Zosterophyllum plant. *New Phytologist* *185*: 217-225.

Haberlandt G. 1914. *Physiological plant anatomy*. London: MacMillan.

Hammer O. 2001. PAST: Paleontological statistics software package for education and data analysis. *Palaeontologia Electronica* 4: 9.

Hotton CL, Hueber FM, Griffing DH, Bridge JS. 2001. Early terrestrial plant environments: an example from the Emsian of Gaspé, Canada. In: Gensel PG, Edwards D, eds. *Plants invade the land: evolutionary and environmental perspectives*. New York: Columbia University Press, 179–212.

Hueber FM. 1971. Early Devonian land plants from Bathurst Island, District of Franklin. *Geological Survey of Canada Paper* 71-28: 1–11.

Hueber FM. 1972. *Rebuchia* *ovata*, its vegetative morphology and classification with the Zosterophyllophytina. *Review of Palaeobotany and Palynology* *14:* 113–127

Hueber FM, HP Banks 1979 *Serrulacaulis furcatus* gen. sp. nov., a new zosterophyll from the lower Upper Devonian of New York State. *Review of Palaeobotany and Palynology* 28: 169–189.

Jensen D, Gensel PG. 2013. *Forania plegiospinosa*, gen. et sp. nov.: a zosterophyll from the Early Devonian of New Brunswick, Canada, with a novel emergence type. *International Journal of Plant Sciences* 174: 687–701.

Kay QON, Daoud HS, Stirton CH. 1981. Pigment distribution, light reflection and cell structure in petals. *Botanical journal of the Linnean Society* 83: 57–83.
