# Supplementary material for: A morphometrics-informed reconstruction of the Early Devonian zosterophyll Nowenia matsunagae gen. et sp. nov. as a template for building detailed empirically supported whole-plant concepts of early tracheophytes with simple body plans
Source: Ann Bot. 2026 Feb 25;137(6):1658–88. doi: 10.1093/aob/mcag040 (PMC13274987; doi:10.1093/aob/mcag040)
Supplement: mcag040_Supplementary_Data [file mcag040_supplementary_data.zip › ElAbdallahEtAl2025_SupplementaryFigures.docx]

**Building detailed and accurate whole-plant concepts: a morphometrics-informed reconstruction of the zosterophyll *Nowenia matsunagae* gen. et sp. nov. from the Lower Devonian of Wyoming**

Samar R. El-Abdallah^1^, Penelope Claisse^2^, Candela Blanco-Moreno^3^, Alexandru M.F. Tomescu^1^*

^1^ Department of Biological Sciences, California State Polytechnic University, Humboldt, Arcata, California 95521, USA

^2^ Evo-Eco-Paléo, École doctorale Sciences de la matière, du rayonnement et de l'environnement, Université de Lille, 59000 Lille, France

^3^ Departamento de Biología, Facultad de Ciencias, Universidad Autónoma de Madrid, 28049 Madrid, Spain

* Author for correspondence: [mihai@humboldt.edu](mailto:mihai@humboldt.edu)

SUPPLEMENTARY FIGURES


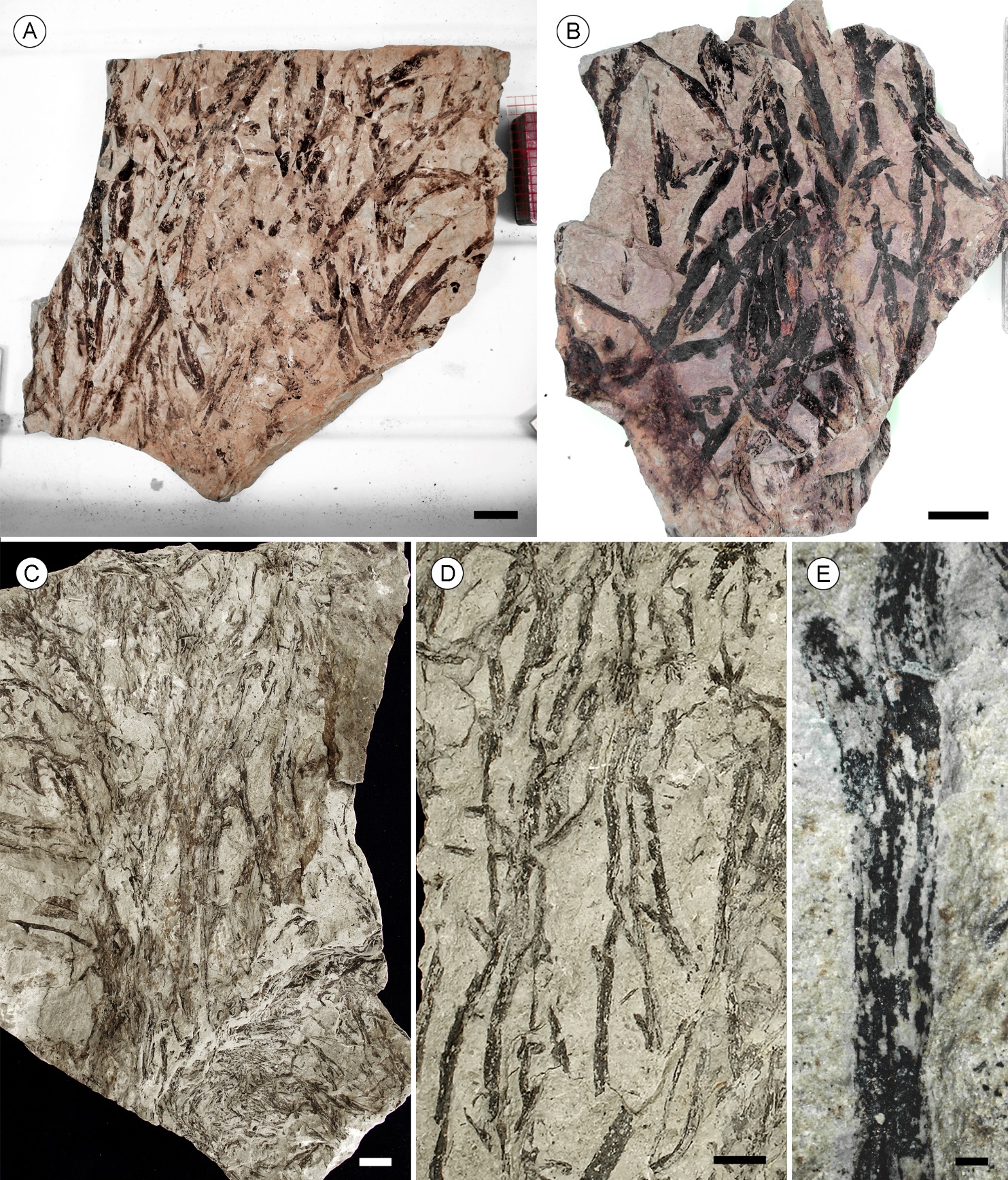


**Supplementary Figure 1.** *Nowenia wyomingense* gen. et sp. nov. (**A-D**) Mats of intertangled axes. Scale bars = 1 cm. (**A**) HPH541. (**B**) HPH581. (**C**) KS D1588c. (**D**) Axes exhibiting drying-induced shrinkage. KS D1588b. (**E**) Axis with elongated knob-like dormant branch (at top left) and preserved with fine longitudinal lines. Scale bar = 1 mm. KS D1588b.


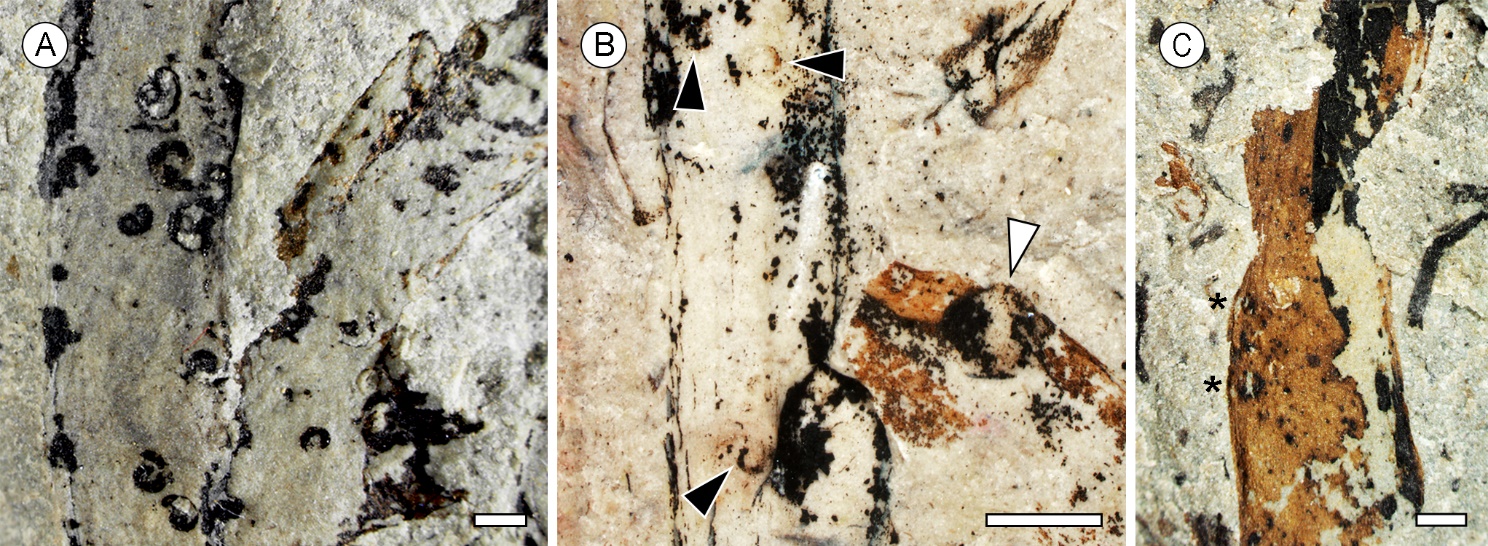


**Supplementary Figure 2.** *Nowenia wyomingense* gen. et. sp. nov. (**A**) Specimen colonized by numerous microconchid invertebrate encrusters. Scale bar = 1 mm. KS D1541b. (**B**) A sterile axis (vertical, at left) bearing microconchids (black arrowheads) and a circinate underdeveloped branch diverging to the right (bottom); and a fragment of fertile axis (oblique orientation, at bottom right) showing a coaly vascular strand and bearing a sporangium (white arrowhead). Scale bar = 2 mm. HPH317. (**C**) Axis preserving cuticular material with darkened areas (e.g., next to the asterisks) interpreted as traces of herbivory. Scale bar = 1 mm. KS D1541b.


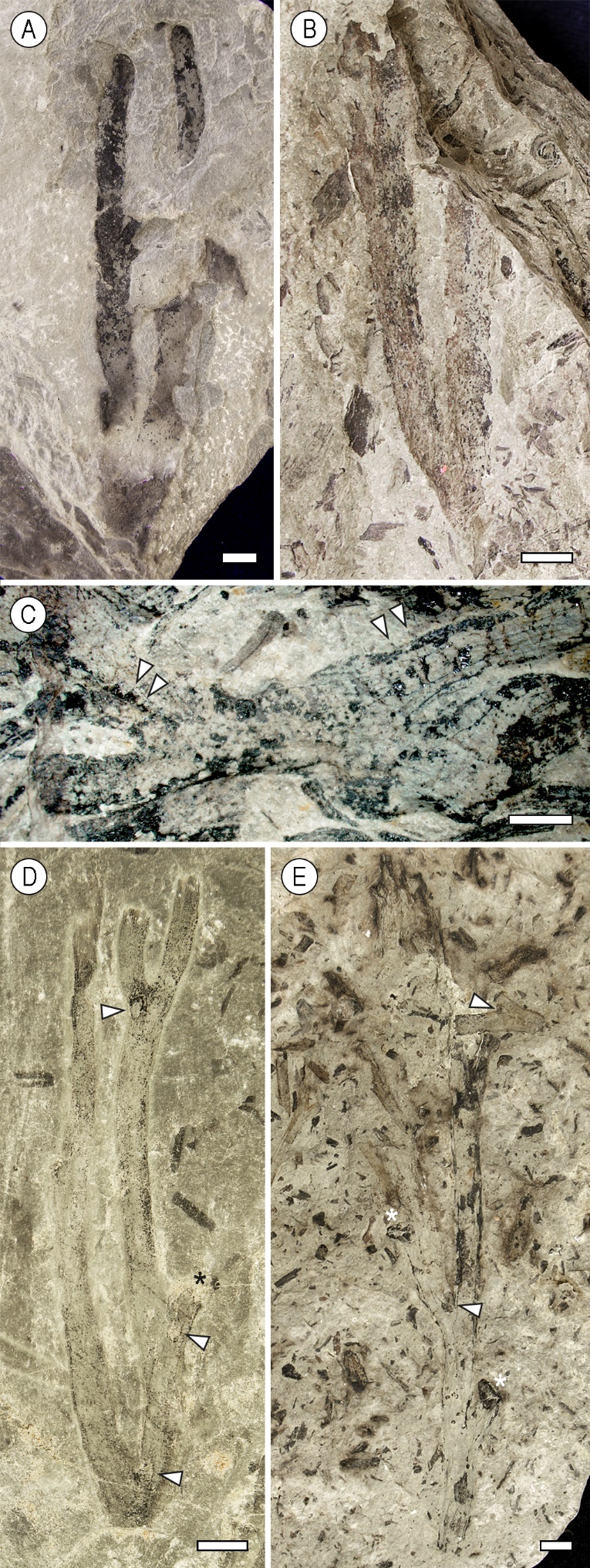


**Supplementary Figure 3.** Two unnamed zosterophyll types from the Cottonwood Canyon locality. (**A**-**C**) “Zosterophyll 1” has thick axes that exhibit U-shaped branching (A, B) similar to *Nowenia*; axes are covered in numerous fine, spine-like projections sometimes preserved as coaly dots on their surface. Scale bars = 5 mm. A: HPH366; B: HPH361. (**C**) K-branching axis covered with fine spine-like projections (arrowheads); branching morphology is more similar to an X, different from that of *Nowenia* (compare with Fig. 5). Scale bar = 2 mm. KS D1434. (**D**-**E**) “Zosterophyll 2”, also exhibiting U-shaped branching, has large subaxillary tubercles (arrowheads) and dormant branch meristems or underdeveloped branches (next to asterisks); some of the axes are colonized by microconchid encrusters (e.g., next to the base of the lower circinate branch in E). Scale bars = 5 mm. D: KS D1181; E: KS D1586.
