# Supplementary material for: A morphometrics-informed reconstruction of the Early Devonian zosterophyll Nowenia matsunagae gen. et sp. nov. as a template for building detailed empirically supported whole-plant concepts of early tracheophytes with simple body plans
Source: Ann Bot. 2026 Feb 25;137(6):1658–88. doi: 10.1093/aob/mcag040 (PMC13274987; doi:10.1093/aob/mcag040)
Supplement: mcag040_Supplementary_Data [file mcag040_supplementary_data.zip › ElAbdallahEtAl2025_SupplementaryTables_R1.docx]

**Building detailed and accurate whole-plant concepts: a morphometrics-informed reconstruction of the zosterophyll *Nowenia matsunagae* gen. et sp. nov. from the Lower Devonian of Wyoming**

Samar R. El-Abdallah^1^, Penelope Claisse^2^, Candela Blanco-Moreno^3^, Alexandru M.F. Tomescu^1^*

^1^ Department of Biological Sciences, California State Polytechnic University, Humboldt, Arcata, California 95521, USA

^2^ Evo-Eco-Paléo, École doctorale Sciences de la matière, du rayonnement et de l'environnement, Université de Lille, 59000 Lille, France

^3^ Departamento de Biología, Facultad de Ciencias, Universidad Autónoma de Madrid, 28049 Madrid, Spain

* Author for correspondence: [mihai@humboldt.edu](mailto:mihai@humboldt.edu)

SUPPLEMENTARY TABLES

**Supplementary Table 1.** Character matrix extracted from the analysis by Claisse *et al.* (2025), used to construct the phylogeny in Fig. 15. For the list of characters, see Supplementary Note; *Zosterophyllum* is scored based on *Z. myretonianum*.

| **Character** | **1** | **2** | **3** | **4** | **5** | **6** | **7** | **8** | **9** | **10** | **11** | **12** | **13** | **14** | **15** |
| --- | --- | --- | --- | --- | --- | --- | --- | --- | --- | --- | --- | --- | --- | --- | --- |
| *Trichopherophyton* | 0 | ? | ? | ? | ? | ? | ? | ? | 1 | ? | 0 | - | - | - | - |
| *Crenaticaulis* | 0 | 0 | 1 | 0 | 1 | ? | ? | 0 | 1 | 1 | 1 | 1 | 1 | 0 | 0 |
| *Deheubarthia* | 0 | 0 | 1 | 0 | 1 | 1 | 0 | 0 | 1 | 1 | 1 | 0 | 0 | 0 | 0 |
| *Forania* | 0 | 0 | 0 | 1 | 1 | 0 | 0 | 1 | 1 | 0 | 1 | 0 | 1 | 1 | 0 |
| *Gosslingia* | 0 | 0 | ? | 0 | 1 | 1 | 1 | 0 | 1 | 1 | 0 | - | - | - | - |
| *Konioria* | 0 | ? | ? | ? | 0 | ? | - | 0 | 1 | 0 | 1 | 1 | 0 | 0 | 2 |
| *Odonax* | 0 | 0 | ? | 1 | 0 | 1 | - | 1 | 1 | 1 | 1 | 1 | 0 | 0 | 1 |
| *Oricilla* | 0 | ? | ? | ? | ? | 0 | ? | 1 | 1 | 0 | 0 | - | - | - | - |
| *Sawdonia* | 0 | 0 | 1 | 1 | 1 | 0 | 0 | 1 | 1 | 0 | 1 | 1 | 0 | 1 | 1 |
| *Serrulacaulis* | 0 | 0 | 0 | ? | 0 | 0 | - | 0 | 1 | 0 | 1 | 0 | 1 | 0 | 0 |
| *Tarella* | 0 | 0 | ? | 1 | ? | ? | ? | ? | 1 | 0 | 0 | - | - | - | - |
| *Thrinkophyton* | 0 | ? | ? | ? | 1 | 0 | 0 | 0 | 1 | 1 | 0 | - | - | - | - |
| *Zosterophyllum* | 0 | 0 | 0 | 1 | 1 | 0 | 0 | 1 | 0 | 0 | 0 | - | - | - | - |
| *Nowenia* | 0 | 0 | ? | 1 | 1 | 1 | 1 | 1 | 1 | 0 | 0 | - | - | - | - |

**Supplementary Table 1.** (continued)

| **Character** | **16** | **17** | **18** | **19** | **21** | **21** | **22** | **23** | **24** | **25** | **26** | **27** | **28** | **29** | **30** |
| --- | --- | --- | --- | --- | --- | --- | --- | --- | --- | --- | --- | --- | --- | --- | --- |
| *Trichopherophyton* | - | 0 | 1 | 0 | - | ? | - | 2 | 1 | ? | 1 | 0 | 1 | 1 | 0 |
| *Crenaticaulis* | 1 | 0 | 1 | 1 | 1 | ? | 0 | 2 | 1 | 0 | 1 | 0 | 1 | 1 | 0 |
| *Deheubarthia* | 1 | 0 | 1 | 1 | 0 | ? | 0 | 2 | 1 | 1 | 1 | 0 | 0 | 1 | 0 |
| *Forania* | 1 | 0 | 1 | 1 | ? | ? | 0 | 2 | 1 | 1 | 1 | 1 | 0 | 1 | 0 |
| *Gosslingia* | - | 0 | 1 | 1 | 1 | ? | 1 | 2 | 1 | 1 | 1 | 1 | 0 | 0 | 0 |
| *Konioria* | 1 | 0 | 1 | 1 | 0 | ? | 0 | 2 | 1 | 1 | 1 | 0 | 0 | 1 | 0 |
| *Odonax* | 1 | 0 | 1 | 1 | 0 | ? | 0 | 2 | 1 | 1 | 1 | 0 | 0 | 1 | 0 |
| *Oricilla* | - | 0 | 1 | 1 | 0 | ? | 1 | 2 | 1 | 1 | 1 | 1 | 0 | 0 | 0 |
| *Sawdonia* | 1 | 0 | 1 | 1 | 0&1 | ? | 1 | 0 | 1 | 1 | 1 | ? | 1 | 1 | 0 |
| *Serrulacaulis* | 1 | 0 | 1 | 1 | 0 | ? | 1 | 2 | 1 | 1 | 1 | 1 | 1 | 1 | 0 |
| *Tarella* | - | 0 | 1 | 1 | 0 | ? | 1 | 2 | 1 | 1 | 1 | 0 | 0 | 0 | 0 |
| *Thrinkophyton* | - | 0 | 1 | 1 | 1 | ? | 1 | 2 | 1 | 1 | 1 | 1 | 0 | 1 | 0 |
| *Zosterophyllum* | - | 0 | 1 | 1 | 0&1 | 1 | 0 | 2 | 1 | 1 | 1 | 0 | 0 | 1 | 0 |
| *Nowenia* | - | 0 | 1 | 0&1 | 0 | ? | 0 | 2 | 1 | 1 | 1 | 0 | 0 | 1 | 0 |

**Supplementary Table 1.** (continued)

| **Character** | **31** | **32** | **33** | **34** | **35** | **36** | **37** | **38** | **39** | **40** | **41** | **42** | **43** | **44** | **45** |
| --- | --- | --- | --- | --- | --- | --- | --- | --- | --- | --- | --- | --- | --- | --- | --- |
| *Trichopherophyton* | ? | ? | ? | ? | ? | ? | ? | ? | 0 | 0 | 0 | 2 | 2 | 2 | 0 |
| *Crenaticaulis* | 1 | 1 | 1 | 0 | - | 1 | 0&1 | 0 | 0 | ? | 1 | 2 | 2 | 2 | 1 |
| *Deheubarthia* | 1 | 1 | ? | 0 | - | 1 | 0&1 | 0 | 0 | ? | 1 | 2 | 2 | 2 | 0 |
| *Forania* | 1 | 0 | - | - | - | 0 | - | - | 0 | ? | ? | ? | 2 | 2 | ? |
| *Gosslingia* | 1 | 1 | 1 | 0 | - | 1 | 0&1 | 0 | 0 | 0 | 1 | 2 | 2 | 2 | 1 |
| *Konioria* | 1 | 0 | - | - | - | - | - | - | 0 | ? | 1 | 2 | 2 | 2 | 1 |
| *Odonax* | 1 | 1 | 1 | 1 | 1 | 1 | 0&1 | 2 | 0 | ? | ? | ? | ? | ? | ? |
| *Oricilla* | 1 | 1 | 1 | 0 | - | 1 | 0 | 0 | 0 | 0 | ? | ? | ? | ? | ? |
| *Sawdonia* | 1 | 1 | 1 | 0 | - | 1 | 0&1 | 0 | 0 | 0 | 1 | 2 | 2 | 2 | 0 |
| *Serrulacaulis* | ? | 1 | 1 | 0 | - | 1 | 1 | 0 | 0 | 0 | ? | ? | 2 | 2 | ? |
| *Tarella* | 1 | 1 | 1 | 0 | - | 1 | 1 | 0 | 0 | 0 | ? | ? | ? | ? | 0 |
| *Thrinkophyton* | 1 | 1 | 1 | 0 | - | 1 | 1 | 0 | 0 | ? | 1 | 2 | 2 | 2 | 0 |
| *Zosterophyllum* | 0 | 1 | 0 | 0&1 | 0&1 | 0 | - | - | 0 | 0 | ? | 2 | 2 | 2 | ? |
| *Nowenia* | 1 | 0 | - | - | - | - | - | - | 0 | ? | ? | ? | ? | ? | ? |

**Supplementary Table 2.** Raw morphometric data of sporangia. Note: measurements of incompletely preserved sporangia are marked "+" to reflect the fact that the respective size was larger than that measured.

| Collection number | Plant specimen | Sporangium width (mm) | Sporangium height (mm) | Width of axis subtending sporangium (mm) | Sporangium orientation w/respect to subtending axis |  |
| --- | --- | --- | --- | --- | --- | --- |
| HPH 317 | A | 1.682 | 1.648 | 3.878 | transverse-oblique |  |
| HPH 317 Back | C | 3.7+ | 2.1 | 4.15 | transverse |  |
| HPH 328 | A | 2.45 | 1.8 | 3.3 | longitudinal-oblique |  |
| HPH 334 | B1 | behind axis | 3.7 | 3.472 | longitudinal-oblique |  |
| HPH 334 | B2 | 3.05 | 3.25 | 3.472 | longitudinal-oblique |  |
| HPH 359 | 4 | 3.05 | 2.3 | 2.45 | longitudinal |  |
| HPH 359 | 5 | 3.2 | 2.35 | 2.7 | longitudinal |  |
| HPH 359 | A1 | 3+ | 1.7 | 3.7 | transverse oblique |  |
| HPH 359 | G2 | 2+ | 1.25+ | 4.15 | transverse |  |
| HPH 359 | G3 | 1.5 | 0.9+ | 3.5 | transverse oblique |  |
| HPH 360 | D | 3.7+ | 3.55 | 3.92 | transverse |  |
| HPH 362 | 3 | 2.0+ | not preserved | 2.452 | longitudinal |  |
| HPH 362 | B1 | 2.0+ | not preserved | 2.929 | longitudinal-oblique |  |
| HPH 362 | A2 | 2.0+ | not preserved | 3 | longitudinal-oblique |  |
| HPH 366 | 8 | 1.6 | 1.4 | 1.865 | longitudinal |  |
| HPH 407 | B2 | 3.6 | 2.3 | 4.1 | transverse |  |
| HPH 465 | 1 | 2.5 | 1.5 | 1.15 | longitudinal | |
| HPH 639 | A1 | 2.45 | 1.8 | 1.75 | longitudinal | |
| HPH 671 | A1 | 1.8 | 1.35 | 3.35 | longitudinal | |
| KS D1480a/b CC79-721a/b | B | 2.555+ | 1.714+ | 3.6 | longitudinal-oblique | |
| KS D1515a/b CC81-105a/b | 1 | 2.674+ | 2.55+ | 2.44 | longitudinal | |
| KS D1515a/b CC81-105a/b | 2 | 2.746 | 1.793+ | 2.44 | longitudinal | |
| KS D1515a/b CC81-105a/b | 3 | 1.609+ | 1.301 | 3.8 | longitudinal-oblique | |
| KS D1547 CC81-123a/b | 1 | 2.69 | 1.456+ | 2.79 | longitudinal-oblique | |

**Supplementary Table 3.** Data of axes used to calculate coefficient of branch subordination (CBS).

| Collection number | Branch developmental stage | Subtending axis width above branching point (mm) - A | | Branch width at base (mm) - B | | Coefficient of branch subordination = B/A | |
| --- | --- | --- | --- | --- | --- | --- | --- |
| HPH 317Back | developed branch | 3.496 | | 3.279 | | 0.937929062 | |
| HPH 328 | dormant meristem | 2.985 | | 1.935 | | 0.648241206 | |
| HPH 328 | circinate branch | 3.107 | | 2.178 | | 0.700997747 | |
| HPH 328a/b | developed branch | 3.31 | | 3.28 | | 0.990936556 | |
| HPH 328a/b | developed branch | 3.502 | | 3.316 | | 0.946887493 | |
| HPH 359 | dormant meristem | 4.357 | | 2.899 | | 0.665366078 | |
| HPH 359 | developed branch | 3.89 | | 3.886 | | 0.998971722 | |
| HPH 359 | developed branch | 4.778 | | 4.633 | | 0.969652574 | |
| HPH 360 | dormant meristem | 5.995 | | 3.724 | | 0.62118432 | |
| HPH 360 | developed branch | 3.89 | | 3.886 | | 0.998971722 | |
| HPH 361 | developed branch | 5.736 | | 5.739 | | 1.000523013 | |
| HPH 362/366 | developed branch | 2.836 | | 2.597 | | 0.915726375 | |
| HPH 362/366 | developed branch | 3.115 | | 2.746 | | 0.881540931 | |
| HPH 385 | developed branch | 3.271 | | 3.117 | | 0.952919596 | |
| HPH 386 | developed branch | 4.3 | | 4.15 | | 0.965116279 | |
| HPH 387 | developed branch | 4.65 | | 4.115 | | 0.884946237 | |
| HPH 388 | dormant meristem | 4.527 | | 2.944 | | 0.6503203 | |
| HPH 388 | circinate branch | 3.443 | | 2.289 | | 0.664827186 | |
| HPH 388 | developed branch | 4.903 | | 4.309 | | 0.878849684 | |
| HPH 541 | dormant meristem | 2.795 | | 1.788 | | 0.639713775 | |
| HPH 541 | circinate branch | 3.551 | | 2.013 | | 0.566882568 | |
| HPH 541 | developed branch | 2.716 | | 2.244 | | 0.826215022 | |
| HPH 638 | circinate branch | 3.737 | | 2.728 | | 0.729997324 | |
| HPH 638 | developed branch | 3.614 | | 3.438 | | 0.951300498 | |
| HPH 640 Front | developed branch | 3.378 | | 3.378 | | 1 | |
| HPH 662 | circinate branch | 4.059 | | 2.717 | | 0.669376694 | |
| HPH 671 | circinate branch | 5.346 | | 3.402 | | 0.636363636 | |
| HPH 671/662 | developed branch | 3.175 | | 3.011 | | 0.948346457 | |
| HPH 671/662 | developed branch | 3.959 | | 3.512 | | 0.8870927 | |
| HPH 690 | dormant meristem | 4.007 | | 2.176 | | 0.543049663 | |
| HPH 769 | developed branch | 4.1 | | 3.78 | | 0.92195122 | |
| KS 1526/1546a (CC81-124a) | developed branch | 3.669 | | 3.086 | | 0.841101117 | |
| KS D1515b (CC81-105b) | developed branch | 4.906 | | 4.35 | | 0.886669384 | |
| KS D1526 | dormant meristem | 4.613 | | 2.683 | | 0.581617169 | |
| KS D1526 | dormant meristem | 3.927 | | 2.129 | | 0.54214413 | |
| PP 15956 | dormant meristem | 4.984 | | 3.377 | | 0.677568218 | |
| PP 15956 | dormant meristem | 4.393 | 3.526 | | 0.802640565 | |  |
| PP 15956 | dormant meristem | 5.034 | 3.713 | | 0.737584426 | |  |
| PP 15956 | circinate branch | 5.13 | 3.494 | | 0.681091618 | |  |
| PP 15966 | dormant meristem | 5.853 | 4.325 | | 0.738937297 | |  |
| PP 15966 | developed branch | 5.566 | 5.556 | | 0.998203378 | |  |
| PP 16097 | dormant meristem | 4.507 | 2.918 | | 0.64743732 | |  |
| PP 49078 | developed branch | 3.603 | 3.427 | | 0.951151818 | |  |
| PP 49079 | circinate branch | 4.582 | 2.984 | | 0.651243998 | |  |
| USNM 598348 | developed branch | 4.718 | 3.832 | | 0.812208563 | |  |

**Supplementary Table 4.** Data matrix used in the Principal Component Analysis (Fig. 8). “Min internode length” refers to the length of shortest internode between two successive mature (fully developed) branches; sporangia were scored as present (1) or absent (0).

| **Fossil specimen** | **Taper coefficient** | **Min internode length (mm)** | **Tuft coefficient** | **Sporangia** |
| --- | --- | --- | --- | --- |
| HPH 328 | 0.018418827 | 6.845 | 0.06433516 | 1 |
| HPH 359 | 0.002672617 | 27.889 | 0.030499997 | 1 |
| HPH 366/362 | 0.020858404 | 8.225 | 0.064358347 | 1 |
| HPH 361 | 0.005971241 | 25.67 | 0.022849135 | 0 |
| HPH 386 | 0.017158223 | 7.483 | 0.048406811 | 0 |
| HPH662/671 | ? | 18.395 | 0.056954095 | 1 |
| KS D1515 | 0.016240421 | 38.022 | 0.034511717 | 1 |
| KS D1526/1546 | 0.002057807 | 7.358 | 0.05279924 | 0 |
| PP 15956 | 0.004397799 | 24.711 | 0.036247644 | 0 |
| PP 15966 | 0.004342442 | 20.559 | 0.038017192 | 0 |
| PP 49074 | 0.002021283 | 30.82 | 0.012405063 | 0 |
| PP 49078 | 0.015171814 | 9.862 | 0.046992481 | 0 |
| USNM 598350 | 0.022134652 | 18.043 | 0.052047705 | 0 |
| PP 49070 | 0.00578676 | 18.043 | 0.026838741 | 0 |
| PP 16097 | 0.01485329 | 24.809 | 0.02825178 | 0 |
| HPH 388 | 0.014983178 | 25.562 | 0.059090305 | 0 |

**Supplementary Table 5.** Summary of results of the PCA (Fig. 8) - eigenvalues and percent variance on each PC axis. For PC1 coordinates of each specimen see Fig. 4 caption.

| **PC** | **Eigenvalue** | **% variance** |
| --- | --- | --- |
| 1 | 2.19349 | 54.837 |
| 2 | 1.03486 | 25.872 |
| 3 | 0.584249 | 14.606 |
| 4 | 0.187396 | 4.6849 |

**Supplementary Table 6.** Variable loadings on each of the PC axes.

| **Variable** | **PC 1** | **PC 2** | **PC 3** | **PC 4** |
| --- | --- | --- | --- | --- |
| Taper coefficient | 0.5248 | 0.13042 | 0.78682 | -0.29748 |
| Min internode length | -0.48739 | 0.55726 | 0.42854 | 0.51794 |
| Tuft coefficient | 0.63288 | -0.05908 | -0.12426 | 0.76193 |
| Sporangia | 0.2941 | 0.8179 | -0.42641 | -0.25042 |
